# Benefits of statistical molecular design, covariance analysis, and reference models in QSAR: a case study on acetylcholinesterase

**DOI:** 10.1007/s10822-014-9808-1

**Published:** 2014-10-29

**Authors:** C. David Andersson, J. Mikael Hillgren, Cecilia Lindgren, Weixing Qian, Christine Akfur, Lotta Berg, Fredrik Ekström, Anna Linusson

**Affiliations:** 1 Department of Chemistry, Umeå University, 90187 Umeå, Sweden; 2 Present Address: Department of Chemistry and Molecular Biology - Medicinal Chemistry, University of Gothenburg, 41296 Göteborg, Sweden; 3 Laboratories for Chemical Biology Umeå, Umeå University, 90187 Umeå, Sweden; 4 Swedish Defense Research Agency, CBRN Defense and Security, 90621 Umeå, Sweden

**Keywords:** Acetylcholinesterase, AChE, Quantitative structure–activity relationship, QSAR, Statistical molecular design, SMD, Covariance matrix, Descriptors, Correlation

## Abstract

Scientific disciplines such as medicinal- and environmental chemistry, pharmacology, and toxicology deal with the questions related to the effects small organic compounds exhort on biological targets and the compounds’ physicochemical properties responsible for these effects. A common strategy in this endeavor is to establish structure–activity relationships (SARs). The aim of this work was to illustrate benefits of performing a statistical molecular design (SMD) and proper statistical analysis of the molecules’ properties before SAR and quantitative structure–activity relationship (QSAR) analysis. Our SMD followed by synthesis yielded a set of inhibitors of the enzyme acetylcholinesterase (AChE) that had very few inherent dependencies between the substructures in the molecules. If such dependencies exist, they cause severe errors in SAR interpretation and predictions by QSAR-models, and leave a set of molecules less suitable for future decision-making. In our study, SAR- and QSAR models could show which molecular sub-structures and physicochemical features that were advantageous for the AChE inhibition. Finally, the QSAR model was used for the prediction of the inhibition of AChE by an external prediction set of molecules. The accuracy of these predictions was asserted by statistical significance tests and by comparisons to simple but relevant reference models.

**Electronic supplementary material:**

The online version of this article (doi:10.1007/s10822-014-9808-1) contains supplementary material, which is available to authorized users.

## Introduction

Many scientific disciplines including medicinal- and environmental chemistry, pharmacology, and toxicology address questions related to the effects of small organic compounds on biological targets, and the relation between the molecules’ physicochemical properties and the observed response. To investigate the chemical structural reasons behind a specific effect and to predict what chemical features an even more (or less) potent compound should have, it is crucial to define a structure–activity relationship (SAR). A SAR establishes a link between the molecular chemical features and a particular measured effect. In this paper, we focus on the importance of careful considerations of the molecules that are used for SAR and quantitative structure–activity relationship (QSAR) studies. The molecules used to establish a QSAR dictate the quality and usefulness of the model, as it is the properties of the molecules that lead to the biological effect we want to model. A prerequisite for (Q)SAR modelling is that the set of included molecules show substantial and statistically significant differences in the measured (biological) effect. The chance of differences in response likely increases if the molecules’ structures are sufficiently diverse—although the statistical significance is dependent on the underlying SAR and the experimental errors of the effect measurements. Furthermore, the chemical features of investigated molecules need to be varied in such a way that their effects can be resolved in the subsequent SAR/QSAR studies. Therefore, we recommend careful selections and investigations of the sets of molecules used for SAR/QSAR in order to improve the usefulness of generated models. Here, we have designed and synthesized a set of inhibitors of the enzyme acetylcholinesterase (AChE) to illustrate the benefits of performing a statistical molecular design (SMD) [1] to create a solid molecular base for SAR and QSAR investigations. We also show the benefits of a careful analysis of the molecules’ properties before modeling, and the assessments of the resulting QSAR in relation to simpler models, here called reference models.

In medicinal chemistry projects, chemists commonly have to select compounds to synthesize, typically less than 100, from a substantially larger theoretical pool of potentially interesting molecules. These selected molecules may be designed and synthesized on a linear time scale (one by one) based on medicinal chemistry experience, which may lead to improved compounds in some cases, but this is not a suitable strategy if the objective is to construct a SAR/QSAR. In such cases, the preferred approach is to design and select sets of molecules that later can be used to investigate the biological effects. In SMD, subsets of molecules are designed based on the principles of design of experiments (DoEs) [2] where chemical features hypothesized to be important for biological effect are varied in a systematic way. SMD offers a way to select subsets of molecules in a sound way from a synthetic- and mathematical point of view, thus aiding chemists to make “smart” subset selections. Selecting compounds based on, for example, D-optimality [3] or by factorial designs [1, 2], effectively reduces the physicochemical overlap between the molecules keeping the number to a minimum. Simultaneously, the design makes sure that the subset is representative of the full set of conceivable molecules, and that chemical features (“synthons” or “building blocks”) return in several molecules to yield a basis for statistically supported conclusions regarding biological effect. More specifically, SMD in SAR analysis makes it possible to investigate non-additive effects of molecule structural or physicochemical features. By designing the molecules through simply combining synthons (building blocks) in a clever way, it can be ensured that structural fragments systematically reappear several times in different combinations among the final molecules. This gives a more robust basis for identifying combination effects and constructing regression models (QSAR). This is achieved because SMD inherently reduces the co-variation of the investigated chemical features increasing the possibility to resolve the impact of each investigated property on the measured biological effect. If two or more chemical features covary, their effects will be confounded and it will be difficult to distinguish what feature that is responsible for the effect. For example, if all flexible molecules are lipophilic, the effect of these two features will be confounded, and it will not be possible to resolve whether the biological effect is dependent mainly on flexibility, lipophilicity, or both. We recommend careful investigations of the correlation patterns of the descriptor-matrices of a set of molecules (i.e., investigation of the covariance of the **X**-matrix) aimed for SAR and QSAR studies. Unfortunately, this is rarely done today even though it is a simple procedure that can be performed for any data (i.e., also non-designed data). Neglecting correlations can result in significant errors in interpretations and wrongful predictions.

There are a large number of techniques for correlating chemical and biological data and perhaps the most common ones are linear methods, such as partial least squares to latent structures (PLS) regression, non-linear regression methods such as neural networks, and decision trees such as random forests [4]. Regardless of method, all models should be properly evaluated for quality and usefulness [5–7], by assessing the covariance of the descriptor matrix, quality of the experimental data, model fit, applicability domain, prediction capability, and interpretation of the resulting relationship. The Organization for Economic Co-operation and Development (OECD) has developed principles for the creation and validation of QSAR models [8] that we encourage modelers to follow. This will allow for an assessment of the quality of the QSAR models, but, although important and necessary, this will not show if the obtained model will add value to the scientific community. We argue that a minimum requirement for publication of a QSAR method should be that it surpasses the performance of simpler methods (reference models, sometimes also called NULL models). These reference models can include the linear regression of biological activity using single physicochemical property of the molecules, such as logP or molecular weight. The usefulness of a more advanced QSAR model should be questioned if a reference model surpasses it in terms of fit and prediction quality.

The compounds designed and synthesized in this study were evaluated for their inhibition of AChE, which is an enzyme present in the nervous system. The enzyme is essential because it hydrolyze the transmitter substance acetylcholine. The active site consists of the entrance site (peripheral anionic site, PAS) and the catalytic site (CAS). Non-covalent inhibitors of AChE are currently used in symptomatic treatment of, for example, Alzheimer’s disease [9]. Covalent inhibitors of AChE, such as phosphorus-based nerve agents (e.g., Sarin), are potent toxins that interfere with the cholinergic signaling. Molecules (antidotes) that cleave the bond between the enzyme and the nerve agent can, assuming favorable circumstances, reactivate enzyme inhibited by a nerve agent. New AChE inhibitors are of great interest to the medical community because many of the current treatments with AChE inhibitors cause grave side effects [10], and most antidotes exhibit a limited blood–brain barrier penetration [9], together with a narrow spectrum in treatment of the intoxication caused by different nerve agents.

QSAR investigations of AChE inhibitors for medical applications started to appear in the late 1990s and among the first was a study by Hansch and co-workers [11] where QSAR-equations based on compounds such as tacrine, carbamates and physostigmine analogues were presented. Since then, many AChE QSAR studies have been presented including carbamates [12–14], analogues of tacrine [15–18], physostigmine [19], donepezil [20–24], 2,5-piperazinedione [25], 4-aryl-4-oxo-*N*-phenyl-2-aminylbutyramide [26], minaprine [27], amaryllidaceae alkaloids [28], and miscellaneous compounds [29, 30]. All of these QSAR studies were based on already existing experimental data of molecules not designed for modeling; SMD was not used in any of the studies and no assessments of the descriptor matrices were presented. Most commonly, previous studies have resulted in 3D-QSARs [13–16, 19, 21, 23, 25–27, 29]. 2D-QSARs presented have usually been based on physicochemical descriptors [17, 22, 24, 28, 30] and/or topology descriptors [18, 20]. The dominating regression method used in these studies was multiple linear regression (MLR) or PLS, but the models were not compared to any reference models and were seldom evaluated with an external test set.

In this study, we have performed SMD to design a set of molecules, included examples of covariance matrix analysis of training set molecules, and performed test set evaluations and reference model comparisons to illustrate the benefits of using these methods in QSAR modeling.

## Results and discussion

### SMD of AChE inhibitors

**Fig. 1:**
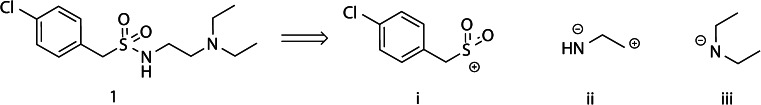
Retrosynthetic analysis of **1** resulted in synthons *i*, *ii* and *iii*

The design of molecules investigated in this study started from compound **1** (Fig. 1), which was discovered in a high throughput screening (HTS) campaign [31], and have been investigated previously for inhibition of AChE [32]. A retrosynthetic analysis of **1** resulted in synthons *i*, *ii* and *iii* (Fig. 1) suitable to form a SMD based on three sets of building blocks in positions p*I*, p*II* and p*III*.

**Fig. 2:**
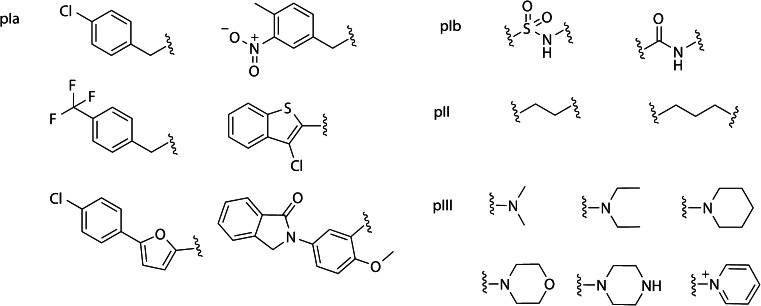
Chemical structures of the three sets of building blocks p*I*, p*II* and p*III* that were selected for the (Q)SAR study; the building blocks correspond to the synthons in Fig. 1, and synthon *i* was further disconnected to the aromatic moiety and the sulfonic amide forming two subsets (p*Ia* and p*Ib*)

The SMD was performed in two steps. The first step was a selection of building blocks to include for p*I*, p*II* and p*III* (Fig. 2) and they were selected based on a SAR analysis of substructures present in hits found in the aforementioned HTS and on commercially available reactants. The aim with the design was to investigate the inhibition effect related to the electronic properties (mainly weakly or strongly electron withdrawing substituents) and bulk of p*I*, and the basicity and bulk of p*III*, with a conservative variation of the linker p*II*. Note that building blocks at p*I* were divided into p*Ia* and p*Ib* to increase the physicochemically diversity of the designed molecules.

**Fig. 3:**
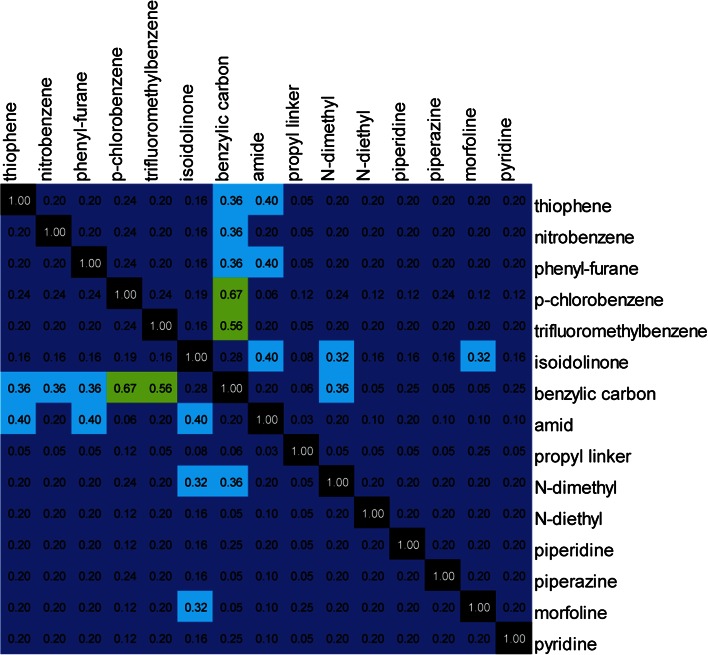
Covariance matrix of conditional descriptors of the selected subset of 18 molecules showing pairwise correlation of the descriptors ranging from minimum (0.00) to maximum (1.00) correlation; descriptor names are given on the axis and colors indicate an increasing covariance *dark blue *to* light blue*,* green*,* orange*,* red* and* black*

The second step of the SMD was a selection of a subset of 18 molecules for synthesis (Table 1). From the 144 possible combinations of the structural fragments at positions p*Ia*, p*Ib*, p*II* and p*III*, the subset was selected to represent the whole set in a balanced way, i.e., balanced with respect to repetition and representation of the structural fragments. This was achieved by applying a D-optimal design [3] on a matrix describing molecules with simple indicators of absence (0) or presence (1) of structural fragments (i.e., conditional descriptors) generated for the 144 molecules (see Online Resource 1 for statistical details and Online Resource 2 for design matrix). The D-optimality criterion assured that the selected molecules reflected the diversity of the 144 candidates. The D-optimal set had a condition number of 1.84 showing that structural fragment were varied independent of each other in the selected set (lower than 3 is preferred [33] ). Importantly, each structural fragment in Fig. 2 was represented at least twice in the subset of 18 molecules and was combined in such way that a subsequent SAR analysis would reveal the influence of each structural fragment. To elucidate possible dependencies, a covariance matrix was calculated (Eq. 1, Fig. 3) on the conditional descriptors. An inspection of the covariance matrix confirmed that there was no strong co-variation in the set. A weak correlation was identified between structural features *p*-*chlorobenzene*/*trifluoromethylbenzene* and *benzylic carbon*, which meant that molecules with a benzylic group at the same time contained a *para*-chlorophenyl or trifluoromethyl-phenyl moiety.

**Table 1:** Chemical structures and AChE inhibition of the 18 compounds in the training set evaluated for AChE inhibition

ID	Name	Structure	*IC*_50_ (µM)	CI^a^ (µM)	p*IC*_50_
**1**	AL011		13.0	11.3–15.0	4.89
**2**	AL013		445	321–616	3.35
**3**	AL012		>1,000^b^	–	2.00
**4**	AL007		69.5	54.6–88.5	4.16
**5**	AL008		101	82.7–123	4.00
**6**	AL006		12.8	11.0–15.0	4.89
**7**	AL015		4,250	1,730–10,500	2.37
**8**	AL016		12.0	10.2–14.0	4.92
**9**	AL005		67.7	57.4–79.8	4.17
**10**	AL014		323	207–504	3.49
**11**	AL009		2,430	1,240–4,790	2.61
**12**	AL010		78.0	65.3–93.1	4.11
**13**	AL017		25.0	21.2–29.6	4.60
**14**	AL021		6.6	5.6–8.0	5.18
**15**	AL022		22.2	19.7–24.9	4.65
**16**	AL018		109	87.2–137	3.96
**17**	AL020		4,204	165–107,000	2.38
**18**	AL019		136	118–156	3.87

^a^Confidence interval (95 %). ^b ^Uncertainty in *IC*_50_ determination due to poor compound solubility

### Synthesis of designed compounds

**Scheme 1:**
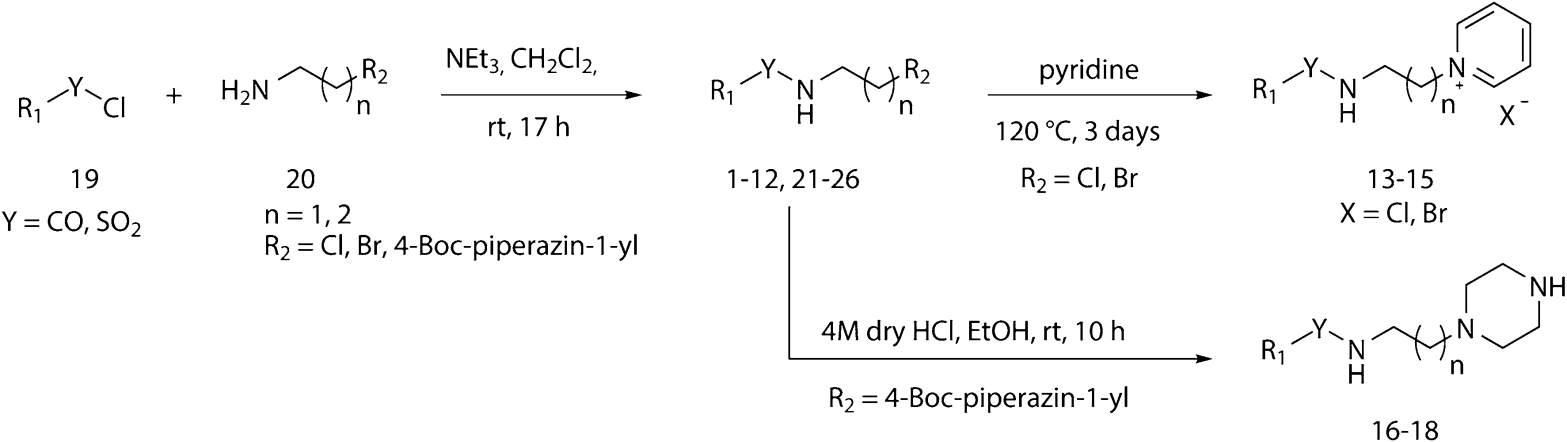
General synthetic scheme detailing synthesis of compounds **1**–**18**

Scheme 1 shows the synthesis of compounds **1**–**18**. Reaction of sulfonyl chlorides **19a**–**d** or acid chlorides **19e**–**i** with amines **20a**–**j** produced compounds **1**–**12** and **21**–**26**. The alkyl halides **21**–**23** were converted into the pyridinium salts **13**–**15** by heating in pyridine, while the piperazinyl compounds **16**–**18** were available from *tert*-butyloxycarbonyl (Boc)-protected intermediates **24**–**26** by protecting group cleavage using 4 M HCl in ethanol. The complete synthetic procedure is given in Online Resource 1 and compound characterization in Online Resource 3.

### AChE inhibition measurements

The training set of 18 compounds was experimentally evaluated for their ability to inhibit the enzymatic activity of AChE (Table 1 and Online Resource 1 for experimental details). The compounds displayed a wide range of activity spanning between a half-maximum inhibition concentration (*IC*_50_) of 6.6 μM (**14**) and 4,200 μM (**3**), with most compounds inhibiting AChE in the low- to mid-micromolar range. Thus, the measured biological response had a sufficient activity range for a SAR/QSAR evaluation, well outside the experimental and acceptable model error.

### SAR and QSAR modelling strategies and considerations

The choice of method to correlate the molecular descriptors to the biological response fell on PLS [34], which is a linear regression method that can account for some non-linearity in the modelling. PLS was selected due to its simplicity and transparency; it is no “black box” and allows for interpretation of the relationship between properties and response. The quality of the resulting PLS regression models was assessed by Pearson’s correlation coefficient (here called R^2^Y) and the root-mean-square error of estimation (RMSEE, Eq. 3), which tells us how well our numerical description of the molecules (our descriptors) could estimate the biological response of the training set. We analyzed our models for robustness (i.e., stability against small changes in the data) using cross validation. Each molecule was sequentially left out once in the model building with subsequent prediction of its p*IC*_50_ value; the correlation coefficient between the internal predictions and the experimental values (Q^2^) of the total training set was reported (Eq. 2). For a robust model there should not be large differences (preferable lower that 0.2 [33] ) between R^2^Y and Q^2^, although it should be noted that the Q^2^ value is highly dependent on the molecules included in the training set [35, 36] and the number of excluded molecules in each cross validation round [37]. The PLS regression coefficients were also robustness-tested by monitoring their variation throughout the cross validation procedure, which was important since the regression coefficients were used to established the SAR and to interpret the QSAR model. We also chose to perform a permutation test [38, 39] to make sure that our model was not a result of chance correlations. In this test, the order of the response values (p*IC*_50_) was scrambled and new models were created that should perform worse than the original model (in terms of R^2^Y and Q^2^).

The number of PLS-components to use in a model requires careful considerations. Too many PLS-components will lead to over fitting and wrongful conclusions regarding the models’ predictive capability. As a guideline, a PLS model including one response variable should not require more than one PLS-component, provided that the relationship between the descriptors and response is linear. In cases with weak non-linearity, PLS will still perform well but one or maximum two additional PLS-components may have to be calculated. The evaluation of the predictive capability of the QSAR model was done by external test sets (i.e., never included in the model building procedure) and by comparisons with reference models, which is the described in more detail in the following sections.

### Structure–activity relationships of AChE inhibitors

In the SAR analysis, the molecules’ inhibition of AChE expressed as p*IC*_50_ (the **Y** matrix) was modeled as a function of the conditional descriptors (absence or presence of structural fragments) used in the SMD (the **X** matrix, see Online Resource 2). The PLS described 79 % of the total variation in **Y** (R^2^Y), had an adjusted R^2^Y of 0.77, and an internal prediction capacity of 26 % [cross-validated Q^2^ (cum), Eq. 2]. The use of a highly reduced subset of 18 out of 144 molecules, with low redundancy in structural features, contributed to the relatively low cross validation value. In other words, predicting the response for a molecule by using the structural information of the other 17 molecules is particularly challenging here since we have designed the molecules to be as different as possible. In fact, it can be showed [35] that the value of Q^2^ as an estimator of the internal prediction capacity decreases with the size of the training set and that Q^2^ is particularly underestimated when calculated on designed data [36]. Leave-many-out would be the preferred method for determining Q^2^ instead of leave-one-out if it is applied to sets of molecules with higher redundancy. The RMSEE was 0.47, indicating an internal estimation error of a half log-unit.

**Fig. 4:**
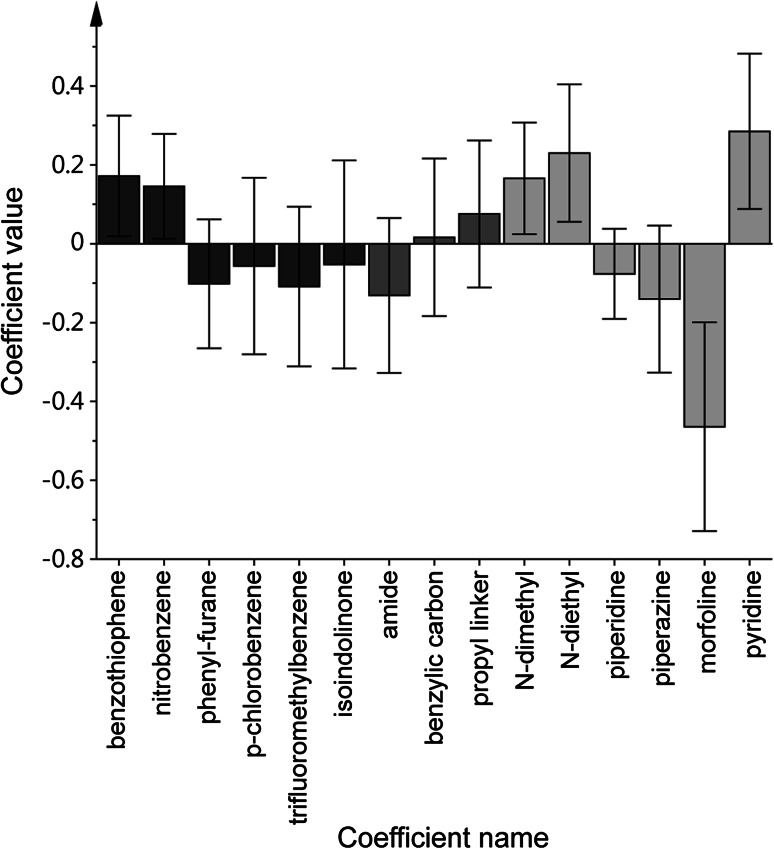
The PLS regression coefficient values showing the influence of the different structural fragments on the inhibition of AChE; aromatic PAS-binding fragments in p*I* are shown in black, linker fragments (in both p*I* and p*II*) in *dark grey*, and basic CAS-binding fragment in p*III* in *light grey*, and confidence intervals (90 %) were calculated using jack-knifing [47] on models generated in the cross-validation procedure

The PLS regression coefficients (Fig. 4) were analyzed to identify how the different structural fragments in the molecules influenced the p*IC*_50_. Most influential were fragments in p*III* binding in the CAS of AChE followed by PAS-binding fragments in p*Ia*, while the effects of changing linker length (p*II*), changing between amide and sulfonamide in p*Ib*, or adding a benzylic CH_2_ were non-significant. From the regression coefficient values, it was clear that *N*-dimethyl, *N*-diethyl or especially pyridinium in p*III*, and a benzothiophene or 4-methyl-2-nitrobenzene in p*Ia* were advantageous for the potency. A morpholine in p*III* was clearly disadvantageous. The benzothiophene and methyl-nitrobenzene substructures has been found before in AChE inhibitors [30], although not combined with the same moieties presented here, while the isoindolinone-phenyl moiety as a PAS binder is novel. The well-known fact that cationic molecules bind to the CAS region of AChE was corroborated here, since the permanent pyridinium cation was the most potent. Notably, common oxime-based antidotes for nerve agent intoxication contain a pyridinium moiety [40], for example pralidoxim and HI-6. No approved drug molecules for Alzheimer’s disease treatment, and only one myasthenia gravis drug (pyridostigmine) targeting AChE contain a pyridinium [41], possibly because of the poor gut absorption and blood–brain barrier passage associated with (permanent) cations. The morpholine as a CAS-binding moiety has been reported before, and is present in the weak AChE inhibitor minaprine and analogues [42]. Similar to our finding here, if compared to other substituents such as piperidinyl and triethylamin, morpholinyl have been shown to be less potent [42, 43]. Nevertheless, morpholinyl per se cannot be considered a poor binder of AChE since it is present in inhibitors in the nM to µM range [30, 44–46].

### Descriptor covariance and QSAR analysis

**Fig. 5:**
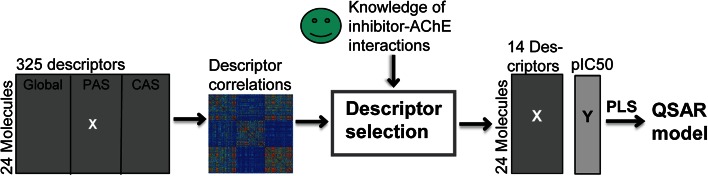
QSAR model building approach where descriptors first were filtered (descriptor selection) based on the covariance matrix and knowledge of important molecular physicochemical properties for AChE inhibition followed by PLS regression to yield the QSAR-model

The SAR analysis was extended by calculating quantitative molecular physicochemical descriptors aiming for QSAR modeling of the AChE inhibition expressed as p*IC*_50_ for the training set molecules. The training set consisted of 24 molecules, which included the original 18 molecules but with both cationic and neutral protonation states for molecules with morpholine or piperazine moieties. Descriptors were calculated for the whole molecules (global descriptors) as well as for sub-structures of the molecules corresponding to the PAS- and CAS-binding moieties, giving 325 descriptors (Fig. 5, and Online Resource 2). It is important to stress that, even though the SMD resulted in a set of molecules with systematically and independently varied structural fragments, the molecule selection was not performed in physicochemical descriptor space. Therefore, it was of particular importance to perform a careful analysis of the physicochemical descriptor matrix to detect dependencies before further modeling (Fig. 5). Accordingly, the covariance matrix of all 325 descriptors of the 24 molecules was calculated to identify descriptor correlations (Eq. 1, and see Online Resource 2). Two descriptors could correlate for three reasons. (1) The descriptors described the same molecular property (e.g., molecular weight and the number of heavy atoms both describe size). (2) The two descriptors correlated just by chance. The risk of chance correlation increase with number of pairwise comparisons, e.g., the risk of chance correlations at a 0.05 significance level is 1–0.95^K^ for K comparisons. (3) The two descriptors correlated due to co-variation of two chemical features within the molecules in the set (e.g., the benzylic fragment and *para*-substituted aromatic fragments in this set). All of this will influence the QSAR modeling in terms of model quality, including interpretation and prediction capacity.

The analysis of the covariance matrix revealed that the main part of the descriptors were related to the size and flexibility of the molecules; 35 % (40 out of 113) of the global descriptors had a correlation coefficient larger than 0.7 compared to the surface area or number of rotatable bonds. It was clear that the descriptors describing electronic properties and (partial) charge distributions, which are of particular interest here, were less redundant than for example size and lipophilicity. In cases where descriptors such as indices and binned descriptors correlated with more interpretable physicochemical descriptors, the latter were selected. We performed a careful selection (Fig. 5) of a subset of 14 descriptors (Table 2) out of the 325 to be used in the QSAR modeling aiming to keep the descriptor redundancy low and avoiding chance correlations. Regarding correlations due to co-variation of chemical features within the molecule set, it is important to include such descriptors in order to keep track of the confounding pattern later on in the modeling. We made the selection to included descriptors with (1) low internal co-variation as determined from the covariance matrix of all 325 descriptors, and (2) a rational relevance for the molecular interaction between the inhibitors and AChE based on previous published results. We emphasize that no account was taken of the correlation of descriptors to the inhibition of AChE in the selection procedure; the selection was solely focused on the **X**-matrix.

**Table 2:** Descriptor name [48] and explanation for descriptors included in the QSAR model

Global		CAS		PAS	
*b_1rotR*	Fraction of rotatable single bonds	*VSA_FPNEG*	Fractional negative polar vdW surface area	*VSA_FPPOS*	Fractional positive polar vdWs surface area
*logP(o/w)*	Log of the octanol/water partition coefficient calculated from a linear atom type model	*VSA_FPOS*	Fractional positive vdW area	*AM1_LUMO*	Energy (eV) of the lowest unoccupied molecular orbital
*TPSA*	Polar surface area (Å^2^)	*VSA_FPPOS*	Fractional positive polar vdW surface area	*npr1*	Normalized principal moment of inertia
*vdw_area*	Area of vdW surface (Å^2^)	*AM1_HOMO*	Energy (eV) of the highest occupied molecular orbital		
*rgyr*	Radius of gyration	*AM1_LUMO*	Energy (eV) of the lowest unoccupied molecular orbital		
		*dipole*	Dipole moment		

**Fig. 6:**
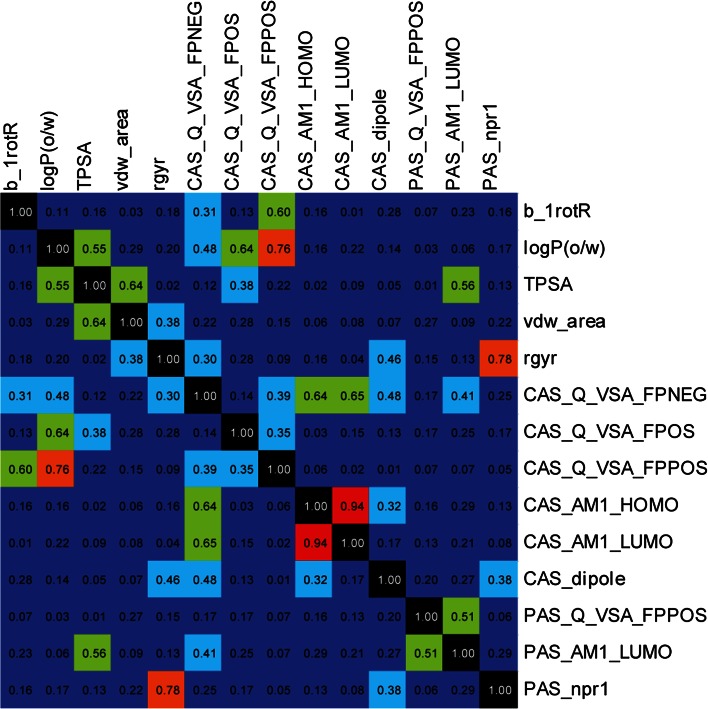
The covariance matrix of descriptors included in the QSAR model and descriptor names are given on the axis and colors indicate an increasing covariance from *dark blue* to *light blue*, *green*, *orange*, *red* and *black*

The covariance matrix of the 14 descriptors used for modeling (Fig. 6) showed, as expected due to the SMD, no correlations between the CAS and PAS descriptors. However, within the subset of CAS descriptors it is clear that the selected structural fragments (building blocks) resulted in a strong correlation (0.94) between highest occupied molecular orbital (HOMO) and lowest unoccupied molecular orbital (LUMO), indicators of polarizability, making it impossible to resolve these effects. It can also be seen that it is the structural fragments in CAS that dictate the lipophilicity of the molecules (i.e., correlation between *CAS_Q_VSA_FPPOS* and *logP* of 0.76) and the PAS structural fragments that is responsible for the variations in shape between the molecules (i.e., correlation between *PAS_npr1* and *rgyr* of 0.78).

**Fig. 7:**
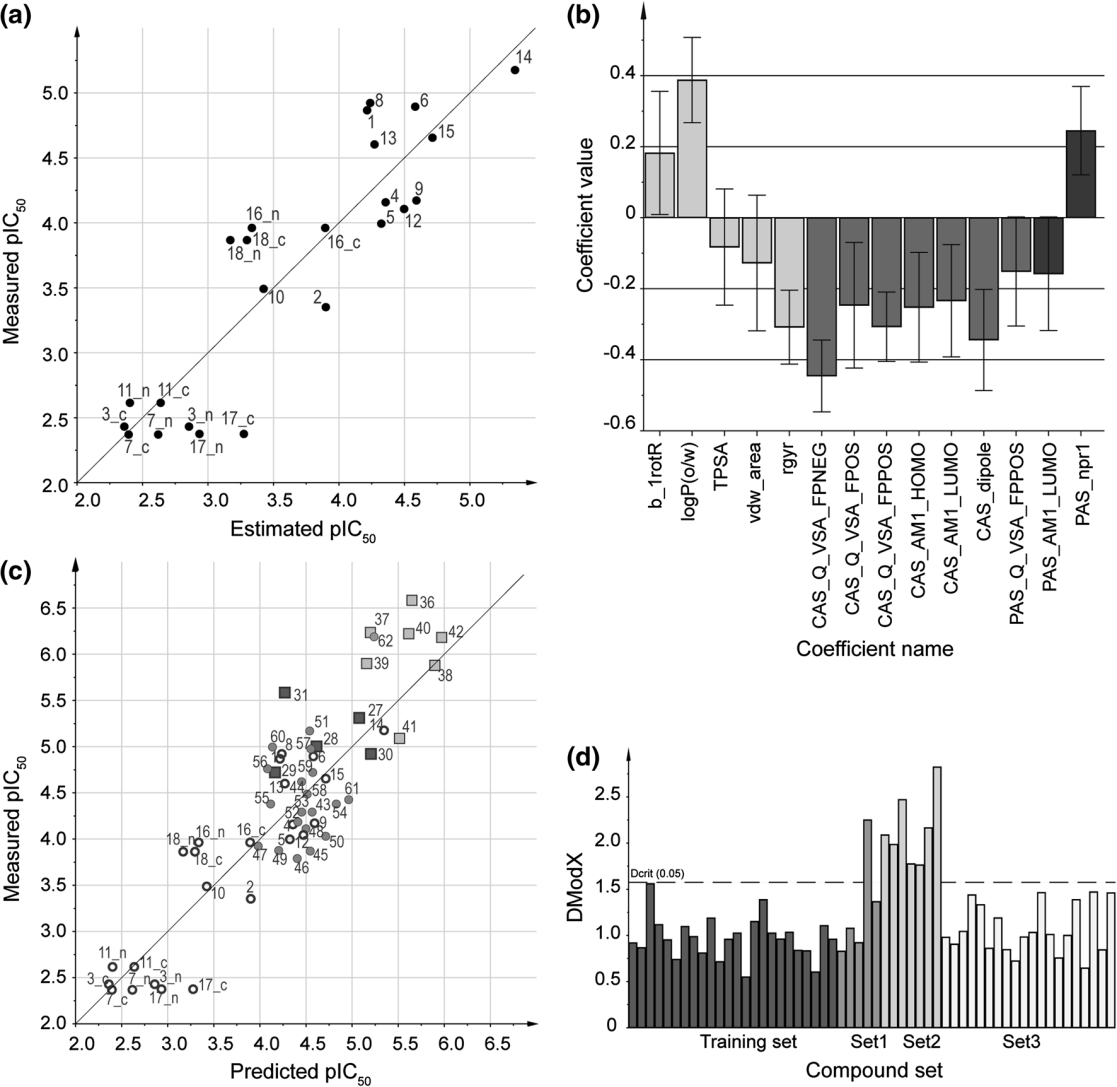
QSAR model based on PLS with **a** measured versus estimated values of p*IC*_50_ for molecules included in the model, where c and n indicates cationic and neutral molecules, respectively, **b** regression coefficients of descriptors where prefixes CAS and PAS indicates descriptors calculated for sub-structures binding in the CAS and PAS of AChE, respectively, and confidence intervals (90 %) were calculated using jack-knifing [47] on models generated in the cross-validation procedure,** c** measured versus predicted p*IC*_50_ values, including the prediction sets **Set1** (*black squares*), **Set2** (*gray squares*) and **Set3** (*gray dots*) and the training set (*black unfilled circles*) for comparison, where c and n indicates a cationic and neutral molecule, respectively, **d** applicability domain assessment using the distance to model in **X** (DModX, Eq. 4) of the prediction set and training set molecules, where DCrit 0.05 represents the 95 % confidence limit of the training set molecules

The QSAR model contained two PLS-components described 79 % of the total variation in **Y** (R^2^ **Y**(cum)), an adjusted R2Y(cum) of 0.77, and an internal prediction capacity of 60 % [Q^2^(cum), Eq. 2]. The p*IC*_50_ values estimated by the model versus the measured values show a linear relationship (Fig. 7a) with a RMSEE of 0.46. A permutation test indicated that the model was not the result of chance correlations between **X** and **Y** (see Online Resource 1).

The regression coefficient plot (Fig. 7b) of the first PLS-component (72 % of the variation) revealed that the strongest inhibitors in the set generally had a higher logP (*logP*(*o/w*)), relatively more rotational bonds (*b_1rotR*), and a smaller radius of gyration (*rgyr*) and were thus more globular. The CAS-binding moiety of the better inhibitors generally had smaller and more polar van der Waals (vdW) areas (*CAS_Q_VSA_FPNEG/CAS_Q_VSA_FPPOS*), lower dipole moments (*CAS_dipole*), and lower energy of the HOMO and/or LUMO (cannot be resolved due to confounding). Furthermore, the stronger inhibitors had an asymmetric PAS moiety in terms of principal moment of inertia (*PAS_npr1*) and lower LUMO energy of the PAS-binding moiety. The importance of a low LUMO energy of the PAS binding moiety corroborates previous findings [30, 45] indicating that an aromatic systems with a high reduction potential may be preferential in PAS. It is a known fact that a positive charge—manifested here in a small and more polar vdW area of the CAS-binding moiety—is important for AChE interactions. Cations have been shown to interact with aromatic side chains in the CAS region in numerous crystal structures, e.g., PDB code 1ACJ [49]. We included both protonation states of molecules containing piperazinyl and morpholinyl moieties with the argument that they possibly could be neutral upon binding, which could influence their inhibition of AChE (AChE preferably bind cationic ligands). Notably, these molecules were moderate inhibitors at best and little difference were seen between charged and neutral states in the model (Fig. 7a), indicating that their poor inhibitions of AChE were not related to their protonation states. The rigorous molecular design and evaluation thereof guarantees that the conclusions drawn here regarding the molecular properties’ influence on compound inhibition of AChE, are indeed certain, within the applicability domain of these molecules.

### Predictive capability of the QSAR model

**Fig. 8:**
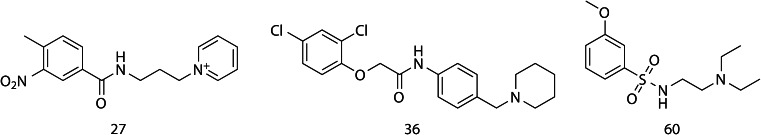
Representative molecules of prediction sets **Set1** (**27**), **Set2** (**36**), and **Set3** (**60**)

Three external test sets (never included in the QSAR model development) were used to evaluate the QSAR model, and examples of these molecules are shown in Fig. 8 (see Online resource 1 for a complete list). **Set1** included molecules **27**–**31** (5 compounds) that were synthesized as a prediction set for the original design. **27**–**29** contained new combinations of structural feature that were found to be beneficial in the SAR, e.g., **27** with 4-methyl-2-nitrobenzene in p*I* and pyridinium in p*III*. **30** and **31** contained “the medicinal chemists’ choice” of structural features, e.g., nitrobenzene in p*Ia* and a thiazole in p*III*. **Set2** included molecules **36**–**42** (7 compounds) that consisted of structural fragments not included in the original design, i.e., the same fragmentation scheme does not apply, leading to more challenging predictions. **Set3** consisted of **43**–**62** (20 compounds) [32], where only p*Ia* was structurally varied; p*Ib*, p*II* and p*III* consisted of a 1-(diethylamino)-2-(sulfonylamino)ethane moiety. Hence, only the PAS binding part has been considered in the predictions for **Set3**.

The three test sets differed in activity ranges where *IC*_50_ was in **Set1** between 2.6 and 19 µM, in **Set2** between 0.3 and 1.3 µM, and in **Set3** between 6.8 and 162 µM with one uniquely active compound (**62**) at 0.7 µM. Prediction **Set4** combined all compounds from the first three prediction sets giving 32 compounds and an overall activity range between 0.3 and 162 µM (p*IC*_50_ 3.79–6.59).

The predicted inhibition capacity of the test set versus the experimental measurements is presented in Fig. 7c. The overall root-mean-square error of the predictions [RMSEP, Eq. 5] for the test sets was 0.57 (Table 3), which is in the same magnitude as the training set RMSEE of 0.46. The test sets were different in terms of prediction errors and distributions of the predicted values (Fig. 7c; Table 3). Molecules in **Set1** that contained new combinations of structural fragments from the training set were indeed among the most active molecules in that class. Predictions to distinguish the activity within the set were, however, not successful (reflected in the RMSEP value of 0.68) due to the low resolution of the model, and the low activity span of one p*IC*_50_ unit of **Set1**. Similar trends could also be seen for **Set2** and **Set3**. Molecules of **Set2** were different in terms of physicochemical properties (DModX, Fig. 7d) and were predicted to be substantially stronger inhibitors than those included in the training set, which was also the case when testing them experimentally. It was not possible to rank **Set2** molecules within the class (p*IC*_50_ between 5.88 and 6.59). The structural changes of the aromatic moiety binding to PAS in **Set3** were predicted to have a moderate effect on the inhibition capacity and it was not possible to predict which structural changes that were more or less beneficial. The exception was **62** that was predicted to be a substantially better binder that the rest of **Set3**, and indeed it was. We concluded that the individual test sets were not appropriate as test sets due to the small activity span and/or low chemical diversity, rather, all three were needed to validate the model. Together the three sets possessed an activity span of two p*IC*_50_ units, which is similar to the training set, but with a (desired) shift in activity from p*IC*_50_ of 2.37–5.18 (the training set) to p*IC*_50_ of 3.79–6.58 for the test set.

**Table 3:** QSAR- and reference model statistics including goodness-of-fit and RMSEP (Eq. 5)

Model	R^2^	RMSEP			
		Set1	Set2	Set3	Set4
QSAR	0.77^a^	0.68	0.67	0.50	0.57
Average	–^b^	1.52	2.44	0.95	1.49
Median	–^b^	1.23	2.14	0.70	1.25
Nearest neighbor	–^c^	0.44	2.10	0.69	1.13
logP	0.38	1.32	0.88	1.03	1.05
TPSA	0.01	1.63	2.35	0.95	1.48
vdW area	0.03	1.33	2.60	0.93	1.51
PLS	0.41^a^	0.93	0.73	1.14	1.03

^a^R^2^Y adjusted. ^b ^Not relevant since the variance of the average and median y is zero. ^c ^Not relevant because this reference model only concerns the test sets

### QSAR model predictions in comparison to simple reference models

To evaluate the quality and usefulness of the QSAR model further, it was compared to seven simple reference models also based on the training set molecules. Three non-regression based methods were used to predict the response values of the molecules in the test set, the average and median of the experimental p*IC*_50_ values of the training set, and a nearest neighbor estimation, based on the assumption that similar molecules have similar biological activity. For each molecule in the test set, we let experienced synthetic- and medicinal chemists at the department, not previously involved in the project, perform unprejudiced selection of the most similar molecule in the training set (without knowing any response values). In addition, we calculated four linear regressions using each of the descriptors logP, TPSA, and vdW area, and a PLS model based on the three descriptors (Table 3). For the regression-based reference model predictions, the training set p*IC*_50_-values showed a weak correlation with logP (R^2^ of 0.38) but no correlation with TSPA, or vdW area (see Online Resource 1). The reference model “PLS” based on the descriptors logP, TPSA and vdW area as **X** and the p*IC*_50_ as **Y**, gave a two-component PLS model with R^2^Y(cum) of 0.46 (adjusted R^2^Y(cum) of 0.41) and a cross-validated Q^2^ of 0.32 (cf. the QSAR model statistics of 0.79, 0.77, and 0.60, respectively).

The p*IC*_50_-values of molecules in the external test sets **Set1**–**4** were predicted using the seven reference-models and the prediction errors from all models are presented in Table 3. The reference models’ prediction accuracy was inferior compared to the QSAR model. Generally, median, average, logP, TPSA and vdW area were poor predictors of p*IC*_50_, while the PLS and nearest neighbor reference model performed slightly better.

Analyzing individual reference-model prediction errors for each of the different test sets revealed that the nearest neighbor predictions performed well for **Set1**. This was not surprising, since **Set1** was selected to extract the best compound features out of the training set, a selection performed by chemists (although not the same chemists that created the reference model). Median and nearest neighbor values seem to be a reasonable p*IC*_50_ predictor for **Set3**; the linear regression based on logP gave reasonable predictions for **Set2**, while the PLS model performed relatively well for **Set1** and **Set2**. None of the reference models was comparable to the QSAR model in prediction capacity of the total set of all test set molecules (**Set4**).

So far, we have investigated and compared the prediction error of the QSAR and reference models, now we analyze if the predicted p*IC*_50_ values resulting from the models were significantly different from the measured p*IC*_50_-values. We assume that the predicted and measured values are equal (the null hypothesis) and tested whether this holds (with a 95 % confidence limit) or should be overruled by the alternative hypothesis (that they differ). This was tested using a parametric *F* test for equal variance and a paired student *t* test for equal mean (in case of normally distributed data (according to Anderson–Darling (AD) test, [50]) and non-parametric tests (Kolmogorov–Smirnov (KS) [51, 52] and Mann–Whitney (MW) *U* test [53, 54] ), which are less sensitive to non-normal distributions within samples. The tests showed satisfactory results; the QSAR models’ predictions are equal to the measured (they are drawn from the same distribution with a probability *p* > 0.05), that is, the prediction values of the inhibition capacity of molecules in the different test sets were not different from the experimental values (Table 4). The test results of the reference models further strengthen the usefulness of the QSAR model; the predictions of the test sets by the reference models gave values that are not significantly equal (*p* < 0.05) to the experimental data as the null hypothesis was rejected for most reference models (except for the nearest neighbor predictions of **Set3**; Table 4). Not all models and prediction sets could be tested by all statistical tests, since there are different criteria that need to be fulfilled (see the Experimental Section and Online Resource 1).

**Table 4:** Statistics test presenting *p* values including, *t* test, Kolmogorov–Smirnov and Mann–Whitney, comparing the predicted p*IC*_50_ values from the QSAR or reference models to the measured p*IC*_50_ values

Test/Model	**Set1**	**Set2**	**Set3**		**Set4**		
	*t* test^a^	*t* test^a^	*t* test^a^	KS^d^	*t* test^a^	KS^d^	MW^e^
QSAR	**0.163**	**0.073**	–^b^	**0.275**	–^c^	**0.518**	**0.330**
Nearest neighbor	–^c^	–^c^	–^c^	**0.275**	–^c^	0.007	0.013
LogP	0.000	0.005	0.000	0.000	–^c^	0.000	0.000
vdw	–^b^	–^b^	–^c^	0.000	–^c^	0.000	0.000
TPSA	–^c^	–^c^	–^c^	0.000	–^c^	0.000	0.000
PLS	0.003	0.030	–^c^	0.000	–^c^	0.000	0.000

^a^Paired (two-tailed) students *t* test where *p* < 0.05 rejects null. ^b ^Did not pass the one-tailed *F* test where calc. >crit. rejects null. ^c ^Non-normally distributed data was not used in *F*/*t* tests. ^d ^Kolmogorov–Smirnov test where *p* < 0.05 rejects null. ^e ^Mann–Whitney test where *p* < 0.05 rejects null

For the individual test sets, one or more reference models were significantly different from the measured p*IC*_50_-values. Importantly, the evaluation was dependent on the size and the composition of the prediction sets, the smaller they were the greater the uncertainty, represented with a higher F- or t critical value. **Set1** included molecules structurally similar to the training set molecules but the prediction errors were as high for this set as for the more dissimilar **Set2**, which may be somewhat surprising. Nevertheless, the statistics showed that the predictions for **Set2** was more uncertain, illustrated by higher F- and t-values compared to **Set1**. The nearest neighbor models predictions of **Set3** p*IC*_50_ was statistically equal to the measured although this model was not successful in predicting all test set molecules (**Set4**). The statistical tests in Table 4 and the prediction errors in Table 3 confirmed that predictions by the investigated reference models were significantly less successful than the QSAR model (see Online Resource 1 for *F* and *t* test details).

## Conclusions

A strategy for the design and assessments of sets of molecules and evaluation of SAR and QSAR models has been presented that showed the benefits of thinking ahead and using SMD and co-variation analysis when planning a SAR/QSAR investigation. A set of inhibitors of the enzyme AChE was designed using SMD that yielded molecules with diverse structures but with repeating structural fragments. This is very important in order to avoid confounding in the measured effects, which would lead to wrongful conclusions in subsequent SAR and QSAR modeling. Co-variation patterns were analyzed through covariance matrices simply calculated from a conditional descriptors matrix. The designed compounds were shown to inhibit AChE and had a reliable potency spanning from molar to micromolar, with the majority of compounds having an *IC*_50_ in the low micro-molar range.

A PLS-model based on conditional descriptors resulted in a clear and transparent SAR analysis, which could reveal molecule sub-structures that were advantageous for the AChE inhibition. The permanent cation pyridinium, and a benzothiophenyl or 4-methyl-2-nitrophenyl was most advantageous in CAS and PAS, respectively, and a morpholinyl in CAS was detrimental to binding. A QSAR model was calculated based on physicochemical descriptors carefully selected to include molecular properties known to be important in inhibitor-AChE binding and to avoid descriptor correlations. The model showed good statistics in terms of model fit, cross-validation and no chance correlations. The QSAR model was used to satisfactory predict the p*IC*_50_ of molecules in three prediction sets. Combinations of the most advantageous sub-structures identified in the SAR-model, i.e., 4-methyl-2-nitrophenyl and pyridinium, gave a molecule with higher p*IC*_50_ than any in the training set. The importance of the test set was highlighted by using sets with different activity spans. A set of simple albeit relevant models, reference models, were calculated and these models were proved statistically to be inferior to the QSAR model in terms of training- and test set p*IC*_50_ predictions.

Much effort has been made to encourage the SAR and QSAR community to adopt some simple benchmarks to improve the quality of models. We believe that the strategy presented here of compound design and evaluation, serves to illustrate the value of SMD, covariance analysis and statistical tests in molecular design and QSAR modeling, and hope that this will inspire to improve QSAR modelling.

## Experimental section

### Statistical molecular design and covariance matrices

D-optimality and covariance matrices based on conditional descriptors from the SMD were calculated from a binary matrix where molecules were described by the presence (1) or absence (0) of a certain structural feature (see Online Resource 2 for matrix). All combinations of the molecular fragments in Fig. 2 resulted in a set of 144 possible molecules, i.e., all possible combinations of all fragments in their respective position p*Ia*, p*Ib*, p*II* and p*III*. A subset was selected out of the 144 using D-optimal design [3]. In D-optimal design, selections are made from **X** (here, the total set of 144 possible molecules with their conditional descriptors) so that the determinant of the matrix **X**_**sel**_’**X**_**sel**_ is maximized (**X**_**sel**_ is the selected set with their conditional descriptors). By maximizing the determinant of the selections, it is assured that the diversity in the designed set is reflecting the diversity of the total set. The selected D-optimal set was evaluated by condition number values of **X**_**sel**_ to investigate whether the structural features were varied independent of each other (where a completely orthogonal design have a value of 1). All descriptors were centered and scaled to unit variance prior to D-optimality calculations and covariance matrix calculation in Matlab [55].

### Molecular descriptor calculation

The molecules’ structures were curated in terms of tautomeric forms and protonation states (MarwinView pk_a_ calculations) [56] in assay conditions pH 8 (Table 5). Note that the some amines in p*III* may be neutral or cationic and both forms were included for ambiguous molecules comprising morpholine (**3**, **7**, and **11**) and piperazine (**16**, **17**, and **18**) The calculations showed that morpholinyl and piperazinyl would be 40 and 2.5 % neutral, respectively, at pH 8. 3D-conformations of the molecules were generated by OMEGA [57, 58] with the MMFF94 s force field [59]. The values for OMEGA parameters *rms* and *ewindow* was set to 0.5 and 40, respectively, and all generated conformations were collected. ROCS [60, 61] was thereafter used to overlay the conformations against an X-ray crystal structure of **36** (AL137) in complex with AChE [31] since the ligands are assumed to bind in an outstretched conformation. The conformation with the highest TanimotoCombo score value was selected and used in calculations of 2D and i3D descriptors in MOE [48]. Descriptors were calculated for the entire molecule (global) as well as the CAS and PAS binding part, p*Ia* + p*Ib* and p*III*, respectively (Table 5).

**Table 5:** The molecular structures for which descriptors were calculated

p*Ia* + p*Ib* (PAS)	p*III* (CAS)
	
	
	
	
	
	
	
	
	

### Covariance matrix calculations

Covariance matrices on descriptors (centered and scaled to unit variance) was constructed by the calculation of correlation coefficients (*ρ*) between pairwise descriptors according to

1 $$ \rho = \mathop \sum \limits_{i = 1}^{N} \frac{{x_{i,1} x_{i,2} }}{N} $$

where x_1_ and x_2_ are values for descriptors 1 and 2 for molecule *i*, and *N* is the number of molecules. Correlation coefficients are reported as absolute values and calculations were done in Matlab [55]. The matrices including conditional- and quantitative descriptors are presented in Online Resource 2.

### Partial least-squares regression

PLS regression [34, 62] was used to correlate the training set descriptor data matrix **X** to the inhibition of AChE (matrix **Y)** using the SIMCA software [63]. The inhibition was expressed as the p*IC*_50_, which is the -log of *IC*_50_ in molar (M) concentration. All descriptors and the response were centered and scaled to unit variance before model building. The quality of the PLS models were determined from the Pearson’s correlation coefficient R^2^ **Y** (derived from the regression between **X** and **Y**, not to be confused with R^2^ **X** which describes the variation in **X** used in the regression), the adjusted R^2^ **Y** (sum-of-squares adjusted for the number of degrees of freedom), and the Q^2^ (derived from cross-validation) according to

2 $$ Q^{2} = 1.0 - {{\text{PRESS}} \mathord{\left/ {\vphantom {{\text{PRESS}} {\text{SS}}}} \right. \kern-0pt} {\text{SS}}} $$

where PRESS is the prediction error sum of squares, SS is the sum of squares. Cross validation was performed by the leave-one-out method. The internal prediction error, i.e., measured *y* versus the fitted *y* or the root-mean-square error of estimation (RMSEE) for the training set, was calculated according to

3 $$ {\text{RMSEE}} = \sqrt {\frac{{\mathop \sum \nolimits_{i = 1}^{N} \left( {y_{i,measured} \times y_{i,estimated} } \right)^{2} }}{N - 1 - A}} $$

where *N* is the number of molecules (*i*) and *A* is the number of PLS-components. The descriptors of the test set molecules were compared to training sets’ to assess the applicability domain by using the normalized distance to model in **X** (DModX) according to

4 $$ {\text{DModX}} = \frac{{\sqrt {\frac{{\mathop \sum \nolimits_{k = 1}^{K} e_{ik}^{2} }}{(K - A)} \times v} }}{{\sqrt {\frac{{\mathop \sum \nolimits_{i = 1}^{N} \mathop \sum \nolimits_{k = 1}^{K} e_{ik}^{2} }}{{\left( {N - 1 - A} \right) \times \left( {K - A} \right)}}} }} $$

where *N* is the number of molecules (*i*), *K* is the number of descriptors, *e*_*ik*_ are the **X**-residuals of molecule *i* for descriptor *k*, *A* is the number of PLS-components, and *v* is a correction factor with a value slightly higher than 1 compensating for the fact that DModX would be slightly smaller for an observation that is part of the model. Predicted molecules significantly dissimilar from the model molecules were identified using normalized DModXPS, which is the same as DModX but without the correction factor *v*. The prediction error of external test molecules i.e., measured *y* versus the predicted *y*, or the root-mean error of prediction (RMSEP) was calculated according to

5 $$ {\text{RMSEP}} = \sqrt {\frac{{\mathop \sum \nolimits_{i = 1}^{N} \left( {y_{i,measured} \times y_{i,predicted} } \right)^{2} }}{N}} $$

where *N* is the number of molecules (*i*). Model validity and chance-correlations between **X** and **Y** were quantified with permutation experiments in SIMCA [63] where the order of p*IC*_50_-values in **Y** was scrambled 200 times and new PLS-models were created and compared to the original model [38, 39]. Two kinds of regression models were derived by PLS calculations between the p*IC*_50_ and (1) the conditional descriptor set and (2) the quantitative descriptors describing the 24 molecules, in the training set. Confidence intervals (90 % confidence limit) for regression coefficients were calculated using jack-knifing [47] on the multiple set of models resulting from the cross validation procedure. The coefficients (centered and scaled to unite variance) were used to interpret the relative importance of the descriptors and the underlying chemical property. A large positive coefficient for a structural feature or descriptor indicated that that feature/descriptor was positively correlated with the p*IC*_50_. Conversely, a negative coefficient indicated that a feature/descriptor was negatively correlated to p*IC*_50_.

### Compound sets for p*IC*_50_ predictions

Three test sets of molecules were used for p*IC*_50_ predictions in the QSAR model. Note that none of these molecules had been part of the QSAR model building. **Set1** included five molecules **27**–**31**. **Set2** included seven analogues **36**–**42** to the molecule C7653, another hit from the HTS [31], and these compounds were synthesized and biologically evaluated here. **Set3** included 20 molecules **43**–**62** that were close analogous to molecule **1** discovered in a HTS [31] and these molecules are reported to be AChE inhibitors [32]. **Set4** included all molecules from **Set1**–**3**. Prediction set molecule structures, synthesis-, and biological data are presented in Online Resource 1.

### Reference model building

Seven simple reference models were calculated for the evaluation and comparison to the prediction power of the QSAR model. Two of the reference models were based on the average or the median of the p*IC*_50_ values for the molecules in the training set. These averages and medians were assumed predicted values of p*IC*_50_ for all 24 molecules, and were compared to the measured p*IC*_50_ in RMSEP calculations according to Eq. (5). Three reference models were linear regressions based on one descriptor—logP, TPSA, or vdW area—and the p*IC*_50_ values. Predicted p*IC*_50_ values from the regression were calculated from the straight-line equations for each individual regression model (see Online Resource 1 for plots and equations), and the RMSEP value from Eq. (5). A PLS-regression model was calculated containing three descriptors logP, TPSA and vdW area and the predicted p*IC*_50_ values from this model were compared to the measured according to Eq. (5). Finally, we let six experienced synthetic/medicinal chemists note for each molecule in the test sets which molecule in the training set they found it most similar to (see Online Resource 1). By consensus, each molecule in the test set was predicted to have the activity of the most similar molecule in the test set. These predictions were called the “nearest neighbor” model.

### Statistical tests of predicted p*IC*_50_ values

QSAR model and reference models predicted p*IC*_50_ for test **Sets 1**–**4** were tested for the probability that they were drawn from a normal distribution using the AD test [50], at a confidence limit of 95 % (*p* = 0.05), implemented in Excel [64, 65]. Alternatives to *F* and *t* test when facing non-normal data are non-parametric tests such as the KS [51, 52] and MW *U* test [53, 54] when the aim is to compare the sample distributions of two sets of data. The number of data points in each set needed to exceed ten and seven in KS and MW, respectively. More details are given in Online Resource 1.

## Electronic supplementary material

Below is the link to the electronic supplementary material.

Online Resource 1: statistical molecular design; synthetic chemistry: methods and results; AChE inhibition assay; QSAR PLS permutation tests; test sets **Set1**, **Set2,** and **Set3** molecules, inhibition data and nearest neighbors; reference model regressions for logP, TPSA and vdW area; Statistical analysis of QSAR model and reference models. (PDF 289 kb)

Online Resource 2: SAR PLS model X-matrix; full QSAR descriptor matrix; covariance matrix for the full QSAR descriptor matrix. (XLSX 1488 kb)

Online Resource 3: NMR and HPLC plots for final compounds **1**–**18**, **27**–**31**, and **36**–**42**. (PDF 4013 kb)
