# Supplementary material for: Benefits of statistical molecular design, covariance analysis, and reference models in QSAR: a case study on acetylcholinesterase
Source: J Comput Aided Mol Des. 2014 Oct 29;29(3):199–215. doi: 10.1007/s10822-014-9808-1 (PMC4330465; doi:10.1007/s10822-014-9808-1)
Supplement: Supplementary file 1 — Online Resource 1: statistical molecular design; synthetic chemistry: methods and results; AChE inhibition assay; QSAR PLS permutation tests; test sets Set1, Set2, and Set3 molecules, inhibition data and nearest neighbors; reference model regressions for logP, TPSA and vdW area; Statistical analysis of QSAR model and reference models. (PDF 289 kb) [file 10822_2014_9808_MOESM1_ESM.pdf]

Benefits of Statistical Molecular Design, Covariance Analysis, and Reference Models in QSAR: a Case Study on Acetylcholinesterase

C. David Andersson,[1] J. Mikael Hillgren,[1,2] Cecilia Lindgren,[1] Weixing Qian,[1,3] Christine Akfur,[4] Lotta Berg,[1] Fredrik Ekström,[4] and Anna Linusson.[1]*

[1] Department of Chemistry, Umeå University, SE-90187 Umeå, Sweden. [2] Current address: Department of Chemistry & Molecular Biology - Medicinal Chemistry, University of Gothenburg SE-41296 Göteborg, Sweden. [3] Laboratories for Chemical Biology Umeå, Umeå University, SE-90187 Umeå, Sweden. [4] Swedish Defense Research Agency, CBRN Defense and Security, SE-90621 Umeå, Sweden.

Table of Contents

**S1**	Contents
**S2**	Statistical molecular design
**S3-S12**	Synthetic chemistry: methods and results
**S13**	AChE inhibition assay
**S14**	QSAR PLS permutation tests
**S15-S17**	Test sets **Set1**, **Set2,** and **Set3** molecules, inhibition data and nearest neighbors
**S18**	Reference model regressions for logP, TPSA and vdW area
**S19**	Statistical analysis of QSAR model and reference models
**S20**	References

Statistical molecular design

SMD [1] may be performed by using a statistical selection method such as D-optimal design, which is especially suitable for molecule selection compared to, for example, factorial designs [1]. Imagine a set of molecules spanning a property space of x dimension, where x corresponds to some descriptors or structural features giving the multidimensional matrix **X**. Molecule property spaces are often irregular in shape, which is a problem for factorial designs but less so for D-optimal selections. A subset of molecules selected for D-optimality would span the multidimensional space with a volume as large as possible (showing in a high log(determinant **X'X**)) and with a spherical geometry (shown by a low condition number). Hence, a D-optimal selection would span an irregular space equally well as a spherical space, but it would show in a higher condition number. The SMD included a selection of a subset of 18 compounds by applying a D-optimal design [2] on a matrix of qualitative descriptors generated for the 144 possible combinations of the structural fragments at positions pIa, pIb, pII and pIII.

Synthetic chemistry: methods and results

Chemicals were purchased from Aldrich Chemical Co. and used without further purification. Reactions were conducted under a nitrogen atmosphere, in dried glassware, using anhydrous solvents unless stated otherwise. Reagent grade CH_2Cl_2 was distilled from calcium hydride. Reagent grade dimethylformamide was distilled under reduced pressure and stored over 4 Å molecular sieves. Column chromatography was performed on silica gel (Merck, 60 Å, 40-63 μm) or using a Biotage SNAP cartridge KP-Sil (10 g) on a Biotage Isolera system. Preparative reverse-phase HPLC for **compounds AL017, AL021, AL022, AL053-AL055, AL048 and AL129** was performed on a Gilson GX-271 instrument fitted with a Machery-Nagel C_{18} HTEC, 5 µm particle size column, using a water/acetonitrile eluent system with a flow rate of 20 mL/min. Analytical reverse phase HPLC for **compounds AL005-AL009, AL011-AL015, and AL017-AL022** was performed with a flowrate of 1 mL/min, a Gilson® 172 diode array detector (detection at 210-360 nm), a Sedere Sedex 85 low-temperature evaporative light-scattering detector (ELSD), and an EC 150/4.6 Nucleodor (C_{18} HTec, 5 μm) column using a water/acetonitrile/formic acid eluent system. Analytical reversed-phased HPLC for **compounds AL010, AL016, AL053-AL055, AL058, AL129, AL137-AL140, AL142, AL150 and AL152** was performed on a Shimadzu® LC-30AD pump (flowrate 1 mL/min) with a Shimadzu SPD-M20A diode array detector (detection at 210-360 nm) an EC 150/4.6 Nucleodor C_{18} HTec, 5 μm column using a water/acetonitrile/formic acid eluent system. ^{1}H NMR and ^{13}C NMR spectra were recorded on a Bruker DRX-400 at 298 K with chemical shift values (δ) being reported in ppm relative to residual protic solvent ($CHCl_3$, δ_H 7.26 ppm, δ_C 77.16 ppm; CD_3OH, δ_H 3.31 ppm, δ_C 49.00 ppm; $CDCl_2$, δ_H 5.32 ppm, δ_C 53.84 ppm). All intermediates were characterized with ^{1}H NMR, and final products were characterized with ^{1}H and ^{13}C NMR and purity of final products was determined from HPLC UV traces.

General procedure 1: formation of amides and sulfonamides. The amine coupling partner (1.2 equiv) followed by triethylamine (1.2 equiv) was added slowly to the relevant acid chloride or sulfonyl chloride (0.45–1.59 mmol, 1 equiv) in CH_2Cl_2 (10 mL) cooled to 0 °C. The solution was stirred at rt for 17 h before being concentrated under reduced pressure. The residue was dissolved in ethyl acetate (15 mL), washed with sat. aq. $NaHCO_3$ (3×2 mL) and brine (2 mL), dried (Na_2SO_4) and concentrated under reduced pressure. The resulting crude material was purified either by precipitation of the hydrochloride salt from a solution of the material in a mixture of CH_2Cl_2, diethyl ether and HCl (1 M in diethyl ether), or by chromatography on silica gel eluting first with 1:9 ethyl acetate/heptane with 0.5% triethylamine added, then with 1:99 MeOH:CH_2Cl_2 with 0.5% triethylamine. In the cases where the acid chloride was not commercially available it was pre-generated as follows: Oxalyl chloride (10 equiv) and a few drops of dimethylformamide were added to the corresponding carboxylic acid (1 equiv) dissolved in CH_2Cl_2 (10 mL) cooled to 0

°C. The resulting mixture was stirred for 40 min before being evaporated to dryness under reduced pressure and used immediately as described above without purification.

General procedure 2: reaction of amines and alkyl halides. K_2CO_3 (2.50 equiv) was added to a solution of the relevant amine (2.50-2.60 mmol, 1.00 equiv) and alkyl halide (1.05 equiv) in acetonitrile (10 mL) and the resulting suspension was refluxed for 24 h. After being allowed to attain rt, sat. aq. $NaHCO_3$ (5 mL) was added to the mixture, the layers were separated, and the water phase was extracted with CH_2Cl_2 (2×10 mL). The combined organic phases were dried (Na_2SO_4) and concentrated under reduced pressure. The crude residue was purified by column chromatography on silica gel (EtOAc:MeOH 5:1).

General procedure 3: removal of Boc groups.

A HCl solution (4.0 M in diethyl ether, 6 ml) was added to the relevant carbamate (0.22-0.40 mmol, 1 equiv) dissolved in EtOH (4 mL). The mixture was stirred at rt for 10 h before being made basic by addition of sat. aq. $NaHCO_3$, and extracted with CHCl3 (3×10 mL). The combined organic layers were dried (Na_2SO_4) and concentrated under reduced pressure. The residue was dissolved in MeOH, adjusted to pH 5 using 10% aqueous HCl solution, and the resulting solution was purified by preparative HPLC (MeCN/water 10 → 60%, 20 mL/min over 30 min).

1-(4-Chloro-phenyl)-*N*-[2-(diethylamino)-ethyl]-methanesulfonamide (1, AL011): using general procedure 1; yield 40%; ^{1}H NMR ($CDCl_3$): δ 7.33 (appt. s, 4H), 4.21 (s, 2H), 2.95 (t, *J* = 5.8 Hz, 2H), 2.50-2.42 (m, 6H), 0.95 (t, *J* = 7.1 Hz, 6H); ^{13}C NMR ($CDCl_3$): δ 134.9, 132.0, 129.0, 128.2, 58.1, 52.0, 46.5, 40.9, 11.7.

1-(4-Chloro-phenyl)-*N*-[3-(piperidin-1-yl)-propyl]-methanesulfonamide (2, AL013): using general procedure 1; yield 44%; ^{1}H NMR ($CDCl_3$): δ 7.35 (appt. s, 4H), 4.18 (s, 2H), 3.11 (t, *J* = 5.6 Hz, 2H), 2.39 (t, *J* = 5.6 Hz, 2H), 2.32-2.22 (m, 4H), 1.64 (tt, *J* = 5.6, 5.6 Hz, 2H), 1.41-1.34 (m, 6H); ^{13}C NMR ($CDCl_3$): δ 134.8, 132.0, 129.0, 128.8, 58.9, 57.3, 54.5, 44.9, 25.8, 24.4, 24.2.

2-(4-Chloro-phenyl)-*N*-[3-(morpholino-4-yl)-propyl]-acetamide (3, AL012): using general procedure 1; yield 99%; ^{1}H NMR ($CDCl_3$): δ 7.30 (d, *J* = 8.4 Hz, 2H), 7.20 (d, *J* = 8.4 Hz, 2H), 6.57 (br s, 1H), 3.63 (t, *J* = 5.1 Hz, 2H), 3.48 (s, 2H), 3.31 (q, *J* = 6.3 Hz, 2H), 2.40-2.36 (m, 6H), 1.64 (tt, *J* = 6.3, 6.3 Hz, 2H); ^{13}C NMR ($CDCl_3$): δ 170.4, 133.8, 133.3, 130.7, 129.1, 66.9, 57.4, 53.7, 43.3, 39.2, 25.0.

***N*-(3-[Dimethylamino]-ethyl)-4-methyl-3-nitrobenzamide hydrochloride (4, AL007):** using general procedure 1; yield 65%; ^{1}H NMR (CD_3OD): δ 8.42 (d, *J* = 1.7 Hz, 1H), 8.07 (dd, *J* = 8.1, 1.7 Hz, 1H), 7.72 (d, *J* = 8.1 Hz, 1H), 3.35-3.25 (m, 4H), 2.96 (s, 6H), 2.65 (s, 3H); ^{13}C NMR (CD_3OD): δ 150.7, 139.9, 139.8, 135.4, 132.0, 124.4, 57.8, 43.8, 39.1, 20.2.

4-Methyl-3-nitro-*N*-[3-(piperidin-1-yl)-propyl]-benzenesulfonamide (5, AL008): using general procedure 1; yield 40%; ^{1}H NMR ($CDCl_3$): δ 8.39 (d, *J* = 1.8 Hz, 1H), 7.95 (dd, *J* = 8.0, 1.8 Hz, 1H), 7.51 (d, *J* = 8.0 Hz, 1H), 3.07 (t, *J* = 5.6 Hz, 2H), 2.66 (s, 3H), 2.42-2.31 (m, 4H), 2.39 (t, *J* = 5.6 Hz, 2H), 1.65-1.59 (m, 6H), 1.49-1.42 (m, 2H); ^{13}C NMR (CD_3OD): δ 149.2, 140.1, 137.9, 133.8, 130.9, 123.3, 59.1, 54.5, 44.8, 26.1, 24.2, 23.4, 20.6.

***N*-(3-Diethylamino-propyl)-4-methyl-3-nitrobenzamide hydrochloride (6, AL006):** using general procedure 1; yield 74%; ^{1}H NMR (CD_3OD): δ 8.44 (d, *J* = 1.9 Hz, 1H), 8.06 (dd, *J* = 8.0, 1.9 Hz, 1H), 7.57 (d, *J* = 8.0 Hz, 1H), 3.52 (t, *J* = 6.6 Hz, 2H), 3.31-3.21 (m, 6H), 2.61 (s, 3H), 2.10-2.03 (m, 2H), 1.34 (t, *J* = 7.3 Hz, 6H); ^{13}C NMR (CD_3OD): δ 168.0, 150.7, 138.1, 134.3, 132.4, 124.5, 50.8, 48.4, 38.0, 25.4, 20.1, 9.1.

2-Methoxy-*N*-[2-(morpholin-4-yl)-ethyl]-5-(1-oxo-1,3-dihydro-isoindol-2-yl)-benzenesulfonamide (7, AL015): using general procedure 1; yield 94%; ^{1}H NMR ($CDCl_3$): δ 8.53 (dd, *J* = 7.5, 2.8 Hz, 1H), 7.92 (d, *J* = 2.8 Hz, 1H), 7.90 (d, *J* = 7.5 Hz, 1H), 7.64-7.58 (m, 1H), 7.60-7.47 (m, 2H), 7.10 (d, *J* = 9.1 Hz, 1H), 5.71 (t, *J* = 5.1 Hz, 1H), 4.87 (s, 2H), 4.00 (s, 3H), 3.74-3.59 (m, 4H), 3.05-2.98 (m, 2H), 2.46 (t, *J* = 5.6 Hz, 2H), 2.40-2.33 (4H, m); ^{13}C NMR ($CDCl_3$): δ 167.7, 152.9, 140.1, 133.1, 132.8, 132.5, 128.7, 127.4, 126.5, 124.3, 122.9, 120.7, 113.2, 66.9, 56.9, 56.6, 53.2, 51.0, 39.6.

***N*-(3-Dimethylamino-propyl)-2-methoxy-5-(1-oxo-1,3-dihydro-isoindol-2-yl)-benzenesulfonamide (8, AL016):** using general procedure 1; yield 90%; ^{1}H NMR ($CDCl_3$): δ 8.56 (dd, *J* = 7.5, 2.8 Hz, 1H), 7.91 (d, *J* = 7.5 Hz, 1H), 7.88

(d, J = 2.8 Hz, 1H), 7.65-7.57 (m, 2H), 7.55-7.47 (m, 2H), 1.72-1.64 (m, 2H), 7.11 (d, J = 9.1 Hz, 2H), 6.59 (br s, 1H), 4.88 (s, 2H), 4.00 (s, 3H), 2.99 (t, J = 6.0 Hz, 2H), 2.35 (t, J = 6.1 Hz, 2H), 2.22 (s, 6H); ^{13}C NMR ($CDCl_3$): δ 167.7, 153.1, 140.2, 132.88, 132.86, 132.5, 128.6, 127.7, 126.5, 124.3, 120.8, 113.2, 58.9, 56.9, 51.1, 45.6, 43.8, 26.0.

3-Chloro-benzo[*b*]thiophene-2-*N*-(3-dimethylamino-propyl)-carboxamide hydrochloride (9, AL005): using general procedure 1; yield 56%; ^{1}H NMR (CD_3OD): δ 7.96-7.89 (m, 2H), 7.57-7.53 (m, 2H), 3.57 (t, J = 6.7 Hz, 2H), 3.27-3.23 (m, 2H), 2.93 (s, 6H), 2.11 (m, 2H); ^{13}C NMR (CD_3OD): δ 163.9, 139.1, 138.0, 132.7, 129.0, 127.0, 124.2, 124.1, 121.7, 56.9, 43.8, 38.0, 26.2;.

***N*-[2-(Piperidin-1-yl)-ethyl]-2-(4-trifluoromethyl-phenyl)-acetamide (10, AL014):** using general procedure 1; yield 92%; ^{1}H NMR ($CDCl_3$): δ 7.59 (d, J = 8.0 Hz, 2H), 7.39 (d, J = 8.0 Hz, 2H), 6.25 (br s, 1H), 3.61 (s, 2H), 3.26 (td, J = 6.1, 4.7 Hz, 2H), 2.35 (t, J = 6.1 Hz, 2H), 2.28-2.23 (m, 4H), 1.41-1.36 (m, 6H); ^{13}C NMR ($CDCl_3$): δ 169.9, 139.4, 129.9, 129.6 (q, J = 32.4 Hz), 125.8 (q, J = 3.7 Hz), 124.2 (q, J = 272.1 Hz), 56.5, 54.1, 43.6, 36.2, 26.0, 24.3.

5-(4-Chlorophenyl)-*N*-(2-morpholinoethyl)furan-2-carboxamide (11, AL009): using general procedure 1; yield 28%; ^{1}H NMR (CD_3OD): δ 7.80 (d, J = 8.6 Hz, 2H), 7.41 (d, J = 8.6 Hz, 2H), 7.16 (d, J = 3.6 Hz, 1H), 6.90 (d, J = 3.6 Hz, 1H), 3.71-3.69 (m, 4H), 3.54 (t, J = 6.8 Hz, 2H), 2.60 (t, J = 6.8 Hz, 2H), 2.55-2.53 (m, 4H); ^{13}C NMR (CD_3OD): δ 163.8, 139.0, 137.9, 132.6, 128.8, 126.9, 124.01, 123.99, 121.5, 56.7, 43.6, 37.9, 26.1.

5-(4-Chloro-phenyl)-furan-2-*N*-[3-(diethylamino)-propyl]-carboxamide hydrochloride (12, AL010): using general procedure 1; yield 50%; ^{1}H NMR (CD_3OD): δ 7.86 (d, J = 8.6 Hz, 2H), 7.43 (d, J = 8.6 Hz, 2H), 7.2 (d, J = 3.7 Hz, 1H), 6.95 (d, J = 3.7 Hz, 1H), 3.52 (t, J = 6.6 Hz, 2H), 3.28-3.20 (m, 6H), 2.11-2.04 (m, 2H), 1.33 (t, J = 7.3 Hz, 6H); ^{13}C NMR (CD_3OD): δ 161.1, 156.3, 147.8, 135.4, 130.1, 129.6, 127.1, 117.7, 108.8, 50.8, 49.9, 49.6, 48.4, 37.2, 25.5, 9.2.

1-(2-[2-(4-Chloro-phenyl)-acetylamino]-ethyl)-pyridinium bromide (13, AL017): Pyridine (16 mg, 0.20 mmol) was added to a solution of **21** (50 mg, 0.18 mmol) in MeCN (1.5 mL) and the resulting mixture heated at 80 °C for 12 h, at which point LCMS analysis indicated complete consumption of the starting material. The mixture was purified by preparative HPLC (acetonitrile/water 10-50%, 20 mL/min over 30 min) to give the salt **13** (27 mg, 42%) as a colourless powder; ^{1}H NMR (CD_3OD): δ 8.87 (dd, J = 6.7, 1.1 Hz, 2H), 8.50 (tt, J = 7.8, 1.3 Hz, 1H), 7.93 (t, J = 6.7 Hz, 2H), 7.36-7.28 (m, 2H), 7.26-7.16 (m, 2H), 4.74 (t, J = 5.6 Hz, 2H), 3.80 (t, J = 5.6 Hz, 2H), 3.44 (s, 2H); ^{13}C NMR (CD_3OD): δ 174.2, 147.0, 146.3, 135.4, 133.9, 131.9, 129.7, 129.2, 62.3, 42.8, 40.7.

1-(3-[(3-Chloro-benzo[*b*]thiophen-2-carbonyl)-amino]-propyl)-pyridinium chloride (14, AL021): Alkyl halide **22** (88 mg, 0.30 mmol) was dissolved in pyridine (0.5 mL) and heated at 120 °C for 3 days, at which point LCMS analysis indicated complete consumption of the starting material. Excess pyridine was removed under reduced pressure and the crude residue purified by preparative HPLC (MeCN/water 10-30%, 20 mL/min over 30 min) to give the salt **14** (93 mg, 83%) as a colourless powder; ^{1}H NMR (CD_3OD): δ 9.17-9.09 (m, 2H), 8.64-8.56 (m, 1H), 8.18-8.09 (m, 2H), 7.96-7.89

(m, 1H), 7.89-7.84 (m, 1H), 7.58-7.49 (m, 2H), 4.80 (t, *J* = 7.3 Hz, 2H), 3.59 (t, *J* = 6.5 Hz, 2H), 2.50-2.36 (m, 2H); ^{13}C NMR (CD_3OD): δ 163.4, 147.0, 138.9, 137.8, 132.7, 129.5, 128.8, 126.9, 124.0, 123.9, 121.4, 60.9, 37.8, 32.3.

1-(3-[(4-Trifluoromethyl-phenyl)-methanesulfonylamino]-propyl)-pyridinium bromide (15, AL022): Alkyl halide **23** (60 mg, 0.17 mmol) was dissolved in pyridine (99 mg, 1.2 mmol) and acetonitrile (2 mL). The mixture was stirred at 90 ºC for 16 h, at which point LCMS analysis indicated complete consumption of the starting material. Solvents were removed under reduced pressure and the crude residue was purified by preparative HPLC (MeCN/water 10-40%, 20 mL/min over 30 min) to give the salt **15** (51 mg, 70%) as a colorless solid; ^{1}H NMR (CD_3OD): δ 9.05-8.99 (m, 2H), 8.60 (tt, *J* = 7.8, 1.2 Hz, 1H), 8.17-8.08 (m, 2H), 7.69 (s, *J* = 8.3 Hz, 2H), 7.64 (d, *J* = 8.3 Hz, 2H), 4.75 (t, *J* = 7.2 Hz, 2H), 4.48 (s, 2H), 3.14 (t, *J* = 6.1 Hz, 2H), 2.30-2.19 (m, 2H); ^{13}C NMR (CD_3OD): δ 147.0, 146.2, 135.8 (q, *J* = 1.4 Hz), 132.7, 131.5 (q, *J* = 3.3 Hz), 129.5, 126.5 (q, *J* = 3.7 Hz), 125.6 (q, *J* = 269.7 Hz), 60.3, 58.2, 40.9, 32.6.

3-Chloro-benzo[*b*]thiophene-2-*N*-[2-(piperazin-1-yl)-ethyl]-carboxamide hydrochloride (16, AL018): starting from **24** using general procedure 3; yield 87%; ^{1}H NMR (CD_3OD): δ 7.93-7.88 (m, 1H), 7.88-7.82 (m, 1H), 7.55-7.47 (m, 2H), 3.59 (t, *J* = 6.2 Hz, 3H), 3.28-3.21 (m, 4H), 2.86-2.78 (m, 4H), 2.71 (t, *J* = 6.2 Hz, 3H); ^{13}C NMR (CD_3OD): δ 162.9, 138.9, 137.9, 133.2, 128.7, 126.8, 124.0, 123.9, 120.8, 57.3, 50.6, 45.0, 38.0.

***N*-[3-(Piperazin-1-yl)-propyl]-C-(4-trifluoromethyl-phenyl)-methanesulfonamide hydrochloride (17, AL020):** starting from **25** using general procedure 3; yield 28%; ^{1}H NMR (CD_3OD): δ 7.70 (d, *J* = 8.3 Hz, 2H), 7.64 (d, *J* = 8.3 Hz, 2H), 4.46 (s, 2H), 3.29-3.18 (m, 4H), 3.09 (t, *J* = 7.0 Hz, 2H), 2.79 (br s, 4H), 2.58 (t, *J* = 7.0 Hz, 2H), 1.79-1.66 (m, 2H); ^{13}C NMR (CD_3OD): δ 136.1, 132.7, 131.4 (q, *J* = 3.8 Hz), 126.4 (q, *J* = 3.8 Hz), 125.6 (q, *J* = 271.7 Hz), 58.3, 56.0, 50.6, 44.5, 42.3, 27.9.

5-(4-Chloro-phenyl)-furan-2-*N*-[3-(piperazin-1-yl)-propyl)-carboxamide hydrochloride (18, AL019): starting from **26** using general procedure 3; yield 60%; ^{1}H NMR (CD_3OD): δ 8.54 (br s, 1H), 7.94-7.72 (m, 2H), 7.52-7.32 (m, 2H), 7.17 (d, *J* = 3.7 Hz, 1H), 6.92 (d, *J* = 3.7 Hz, 1H), 3.45 (t, *J* = 7.0 Hz, 2H), 3.28-3.09 (m, 4H), 2.82-2.63 (m, 4H), 2.53 (t, *J* = 7.1 Hz, 2H), 1.83 (tt, *J* = 7.1, 7.0 Hz, 2H); ^{13}C NMR (CD_3OD): δ 160.7, 156.1, 148.3, 135.4, 130.1, 129.7, 127.1, 117.2, 108.7, 56.5, 51.0, 44.8, 38.3, 27.5.

***N*-(2-Bromo-ethyl)-2-(4-chloro-phenyl)-acetamide (21):** using general procedure 1; yield 87%; ^{1}H NMR ($CDCl_3$): δ 7.36-7.29 (m, 2H), 7.24-7.17 (m, 2H), 5.97 (br s, 1H), 3.61 (dt, *J* = 5.9, 5.8 Hz, 2H), 3.54 (s, 2H), 3.43 (t, *J* = 5.8 Hz, 2H).

3-Chloro-benzo[*b*]thiophene-2-*N*-(3-chloro-propyl)-carboxamide (22): using general procedure 1; yield 61%; ^{1}H NMR ($CDCl_3$) δ 7.85-7.76 (m, 2H), 7.50-7.41 (m, 2H), 7.31 (br s, 1H), 3.74-3.61 (m, 4H), 2.20-2.09 (m, 2H).

***N*-(3-Bromo-propyl)-C-(4-trifluoromethyl-phenyl)-methanesulfonamide (23):** using general procedure 1; yield 40%; ^{1}H NMR ($CDCl_3$): δ 7.66 (d, *J* = 8.2 Hz, 2H), 7.53 (d, *J* = 8.2 Hz, 2H), 4.46 (t, *J* = 6.2 Hz, 1H), 4.31 (s, 2H), 3.42 (t, *J* = 6.2 Hz, 2H), 3.25-3.15 (m, 2H), 1.98-2.09 (2H, m).

***tert*-Butyl-4-[2-(3-chloro-benzo[b]thiophene-2-carboxamido)-ethyl]-piperazine-1-carboxylate (24):** using general procedure 1 (the amine coupling partner, (4-(2-aminoethyl)piperazine)carboxylic acid 1,1-dimethylethylester **20i** was prepared according to literature procedures) [3]; yield 49%; ^{1}H NMR ($CDCl_3$): δ 7.84 (br s, 1H) 7.82-7.70 (m, 2H), 7.49-7.38 (m, 2H), 3.57 (dt, *J* = 5.7, 5.4 Hz, 2H), 3.54-3.51 (m, 4H), 2.61 (t, *J* = 6.0 Hz, 2H), 2.56-2.37 (m, 4H), 1.44 (s, 9H).

***tert*-Butyl-4-(3-[(4-trifluoromethyl-phenyl)-methanesulfonylamino]-propyl)-piperazine-1-carboxylate (25):** using general procedure 1 (the amine coupling partner, (4-(2-aminopropyl)piperazine)carboxylic acid 1,1-dimethylethylester **20j** was prepared according to literature procedures) [3]; yield 44%; ^{1}H NMR ($CDCl_3$): δ 7.65 (d, *J* = 8.1 Hz, 2H), 7.55 (d, *J* = 8.1 Hz, 2H), 7.03 (br s, 1H), 4.27 (s, 2H), 3.21 (br s, 4H), 3.14 (t, *J* = 5.7 Hz, 2H), 2.43 (t, *J* = 5.8 Hz, 2H), 2.30-2.21 (m, 4H), 1.72-1.62 (m, 2H), 1.42 (s, 9H); ^{13}C NMR ($CDCl_3$): δ 154.7, 134.2, 131.1, 131.0 (q, *J* = 32.0 Hz), 125.7 (q, *J* = 3.7 Hz), 124.0 (q, *J* = 271.1 Hz), 79.9, 58.2, 44.7, 28.5, 24.6.

***tert*-Butyl-4-(3-[5-(4-chloro-phenyl)-furan-2-carboxamido]-propyl)-piperazine-1-carboxylate (26):** using general procedure 1 (the amine coupling partner, (4-(2-aminopropyl)piperazine)carboxylic acid 1,1-dimethylethylester **20j** was prepared according to literature procedures) [3]; yield 81%; ^{1}H NMR ($CDCl_3$): δ 7.56 (d, *J* = 8.6 Hz, 2H), 7.51 (br s, 1H), 7.32 (d, *J* = 8.6 Hz, 2H), 7.13 (d, *J* = 3.6 Hz, 1H), 6.68 (d, *J* = 3.6 Hz, 1H), 3.61-3.52 (m, 2H), 3.50-3.40 (m, 4H), 2.50 (t, *J* = 6.2 Hz, 2H), 2.45-2.35 (m, 4H), 1.81-1.75 (m, 2H), 1.42 (s, 9H).

1-[3-(4-Methyl-3-nitro-benzoylamino)-propyl]-pyridinium bromide (27, AL053): pyridine (37 mg, 0.46 mmol) was added to a solution of **32** (140 mg, 0.46 mmol) in MeCN (25 µL) and the resulting mixture heated at 80 °C for 6 h, at which point LCMS indicated complete consumption of the starting material. The mixture was purified by preparative HPLC (MeCN/water 10-50%, 20 mL/min over 30 min) to give the salt **27** (27 mg, 42%) as a white powder; ^{1}H NMR (CD_3OD): δ 9.13 (d, *J* = 5.9 Hz, 2H), 8.61 (t, *J* = 8.0 Hz, 1H), 8.43 (d, *J* = 1.8 Hz, 1H), 8.15 (t, *J* = 7.9 Hz, 2H), 8.08-8.04 (m, 1H), 7.56 (d, *J* = 8.0 Hz, 1H), 4.84-4.75 (m, 2H), 3.54 (t, *J* = 6.6 Hz, 2H), 2.61 (s, 3H), 2.4 (m, *J* = 6.8 Hz, 2H); ^{13}C NMR (CD_3OD): δ 167.9, 150.7, 147.1, 146.3, 138.2, 134.5, 134.4, 132.6, 129.7, 124.7, 61.0, 37.7, 32.4, 20.3.

1-[2-(4-Methyl-3-nitro-benzoylamino)-ethyl]-pyridinium bromide (28, AL054): 4-methyl-3-nitrobenzoyl chloride (0.19 g, 0.95 mmol), **33** (0.19 g, 0.95 mmol) was dissolved in CH_2Cl_2 (2.0 mL) and the solution was stirred at rt for 6 h, at which point LCMS analysis indicated consumption of the starting material. The solvent was removed under reduced pressure. The residue was dissolved in MeOH and purified by preparative HPLC (MeCN:water 1:9, isocratic) which gave **28** (62 mg, 18%) as a white solid after lyophilisation; ^{1}H NMR (CD_3OD): δ 9.06 (d, *J* = 5.5 Hz, 2H),8.62 (t, *J* = 7.7

Hz, 1H), 8.32 (s, 1H), 8.16-8.08 (m, 2H), 7.99-7.92 (m, 1H), 7.58-7.50 (m, 1H), 4.93-4.86 (m, 2H), 4.07-4.98 (m, 2H), 2.59 (s, 3H); ^{13}C NMR (CD_3OD): δ 168.1, 150.8, 147.5, 146.6, 138.5, 134.5, 133.8, 132.5, 129.6, 124.7, 62.6, 41.7, 20.3.

Dimethyl-[3-(4-methyl-3-nitro-benzoylamino)-propyl]-ammonium chloride (29, AL055): 3-(dimethylamino)-1-propylamine (0.10 g, 0.50 mmol) was dissolved in CH_2Cl_2 (2.0 mL) and cooled to 0 °C. 4-Methyl-3-nitrobenzoyl chloride (51 mg, 0.5 mmol) was added slowly and the mixture was stirred at rt for 2 h, at which point LCMS analysis indicated consumption of the starting material. The organic phase was washed with sat. aq. $NaHCO_3$ (2 mL), dried (Na_2SO_4) and concentrated under reduced pressure. The residue was dissolved in MeOH, adjusted to pH 5 (using HCl, 1 M) and purified by preparative HPLC (MeCN/water 10→50%, over 30 min) to yield **29** (68 mg, 45%) as a white solid after lyophilisation; ^{1}H NMR (CD_3OD): δ 8.46-8.43 (m, 1H), 8.09-8.03 (m, 1H), 7.58-7.53 (m, 1H), 3.55-3.49 (m, 2H), 3.26-3.20 (m, 2H), 2.93 (br s, 6H), 2.61 (br s, 3H); ^{13}C NMR (CD_3OD): δ 168.0, 150.7, 138.0, 134.3, 132.5, 124.5, 56.7, 43.6, 37.7, 26.1, 20.1.

3-[2-(2-Nitro-benzenesulfonylamino)-ethyl]-thiazol-3-ium bromide (30, AL058): **34** (15 mg, 0.05 mmol) was mixed with thiazole (8.5 mg, 0.10 mmol) in a microwave tube and heated to 130 °C for 60 min in a microwave reactor [4]. The solid formed was dissolved in CH_3OH and LCMS analysis indicated that the desired product had been formed. The product was purified by preparative HPLC (MeCN/water, 10→50% over 30 min), which gave **30** (15 mg, 76%) as a white solid after lyophilisation; ^{1}H NMR (CD_3OD): δ 10.1 (br 2, 1H), 8.51-8.46 (m, 1H), 8.30-8.25 (m, 1H), 8.07-8.01 (m, 1H), 7.91-7.79 (m, 3H), 4.77 (t, J = 5.6 Hz, 2H), 3.63 (t, J = 5.3 Hz, 2H); ^{13}C NMR ($CDCl_3$): δ 160.9, 149.4, 138.8, 135.5, 134.0, 133.9, 131.5, 127.3, 126.2, 56.5, 44.0.

3-(2-[(5-Nitro-benzo[b]thiophene-2-carbonyl)-amino]-ethyl)-thiazol-3-ium bromide (31, AL129): **35** (17 mg, 0.05 mmol) was mixed with thiazole (17 mg, 0.20 mmol) in a microwave tube and heated at 130 °C for 70 min in a microwave reactor [4]. The formed solid was dissolved in MeOH and LCMS analysis indicated that two main products had been formed. The products were separated by HPLC (MeCN/water, 10→50% over 30 min to yield **31** (12 mg, 58%) as a white solid after lyophillization; ^{1}H NMR ($(CD_3)_2SO$): δ 10.28-10.22 (m, 1H), 9.24 (t, J = 5.8 Hz, 1H), 8.86 (d, J = 2.0 Hz, 1H), 8.66-8.62 (m, 1H), 8.37-8.33 (m, 1H), 8.32-8.21 (m, 3H), 4.83-4.77 (m, 2H), 3.88-3.80 (m, 2H); ^{13}C NMR ($(CD_3)_2SO$): δ 161.4, 160.0, 145.9, 145.4, 142.7, 138.9, 137.5, 126.8, 126.0, 124.3, 120.9, 120.1, 54.2.

***N*-(3-Bromo-propyl)-4-methyl-3-nitro-benzamide (32)**: using general procedure 1; yield 93%; ^{1}H NMR ($CDCl_3$): 8.31 (d, J = 2.0 Hz, 1H), 7.93-7.87 (m, 1H), 7.48-7.39 (m, 1H), 7.35-7.29 (m, 1H), 3.55 (q, J = 6.3 Hz, 2H), 3.43 (t, J = 6.5 Hz, 2H), 2.54 (s, 3H), 2.15 (m, J = 6.6 Hz, 2H).

2-(Pyridinium-1-yl)-ethylamine bromide (33): 2-bromoethylamine hydrobromide (225 mg, 1.1 mmol) was added to pyridine (0.5 mL) and the solution was refluxed for 24 h. Pyridine was removed under reduced pressure and the pH was adjusted to 8 using base (NaOH, 1 M). The water was removed under reduced pressure and the solid residue was

dissolved in MeOH/CH_2Cl_2 1:1. Undissolved material was removed by filtration. Solvents were removed under reduced pressure resulting **33** (193 mg, 86%) as a yellow solid, which were used without further purification; ^{1}H NMR (CD_3OD): 9.21-9.13 (m, 2H), 8.75-8.65 (m, 1H), 8.29.8.15 (m, 2H), 4.94-4.81 (m, 2H) 4.74-4.60 (m, 4H) 3.44-3.29 (m, 2H).

***N*-(3-Bromo-propyl)-2-nitro-benzenesulfonamide (34)**: 2-bromoethylamine hydrobromide (0.12 g, 0.60 mmol) was dissolved in CH_2Cl_2 (3 mL) and cooled to 0 °C. Triethylamine (0.10 g, 1.0 mmol) was added and the mixture was stirred for 5 mins after which 2-nitro-benzenesulfonyl chloride (0.11 g, 0.50 mmol) was added slowly. The mixture was stirred at rt for 2 h, at which point LCMS analysis indicated consumption of the starting material. The organic phase was washed with sat. aq. $NaHCO_3$ (1×1 mL), dried (Na_2SO_4) and CH_2Cl_2 was removed under reduced pressure. The residue was purified by column chromatography (EtOAc) to offer **34** (99 mg, 64%) as a white solid; ^{1}H NMR ($CDCl_3$): δ 8.15-8.07 (m, 1H), 7.91-7.84 (m, 1H), 7.78-7.69 (2H), 5.90-5.76 (m, 1H), 3.56-3.48 (m, 2H), 3.47-3.39 (m, 2H).

5-Nitro-benzo[b]thiophene-2-*N*-(2-bromo-ethyl)-carboxamide (35): adjusted from reference [5]. 5-nitro-benzo[b]thiophene-2-carboxylic acid (0.11 g, 0.50 mmol) was mixed with thionyl chloride (0.30 g, 2.52 mmol) and refluxed for 2 h. The acid chloride (pale yellow solid) was obtained after distillation to remove excess thionyl chloride. 2-Bromoethylamine hydrobromide (0.10 g, 0.5 mmol) was dissolved in CH_2Cl_2 (0.03 mL) and cooled to 0 °C. Triethylamine (0.05 g, 0.50 mmol) was added and the mixture was stirred for 5 min, after which the acid chloride was added slowly. The mixture was stirred at rt for 2 h, at which point LCMS analysis indicated complete consumption of the starting material. The organic phase was washed with sat. aq. $NaHCO_3$ (1×1 mL), dried (Na_2SO_4) and CH_2Cl_2 was removed under reduced pressure. The residue was purified by column chromatography (EtOAc/petrolium ether, 1:1) to offer **35** (129 mg, 78%) as a white solid; ^{1}H NMR ($(CD_3)_2SO$): δ 9.32-9.21 (m, 1H), 8.96-8.88 (m, 1H), 8.35-8.20 (m, 3H), 7.09-5.06 (m, 4H).

2-(2,4-Dichlorophenoxy)-N-(4-(piperidin-1-ylmethyl)phenyl)acetamide (36, AL137): purchased from Chembridge; ^{1}H NMR (CD_3OD): δ 7.57 (d, *J* = 8.7 Hz, 2H), 7.57 (d, *J* = 2.5 Hz, 1H), 7.31 (d, J = 8.7 Hz, 2H), 7.3 (dd, *J* = 8.7, 2.5 Hz, 1 H), 7.1 (d, *J* = 8.7 Hz, 1 H), 4.76 (s, 2H), 3.48 (s, 2H), 2.42 (bs, 4H), 1.60 (p, *J* = 5.7 Hz, 4H), 1.49-1.45 (m, 2H); ^{13}C NMR (CD_3OD): δ 186.2, 154.0, 138.1, 134.6, 131.5, 131.0, 129.1, 128.1, 125.1, 121.2, 116.8, 69.8, 64.1, 55.2, 26.4, 25.2.

2-(4-Chlorophenoxy)-N-(4-(piperidin-1-ylmethyl)phenyl)acetamide (37, AL138): purchased from Chembridge; ^{1}H NMR (CD_2Cl_2): δ 8.21 (bs, 1H), 7.52 (d, *J* = 8.4 Hz, 2H), 7.34-7.29 (m, 4H), 7.00-6.96 (m, 2H), 4.57 (s, 2H), 3.43 (bs, 2H), 2.36 (bs, 4H), 1.58-1.53 (m, 4H), 1.46-1.41 (m, 2H); ^{13}C NMR (CD_2Cl_2): δ 166.0, 156.2, 136.2, 130.1, 130.1, 127.5, 120.2, 120.1, 116.6, 68.3, 63.4, 54.8, 26.4, 24.8.

2-(2-Chloro-4,6-dimethylphenoxy)-N-(4-(piperidin-1-ylmethyl)phenyl)acetamide (38, AL139): purchased from Chembridge; ^{1}H NMR (CD_3OD): δ 7.56 (d, *J* = 8.5 Hz, 2H), 7.35 (d, *J* = 8.5 Hz, 2H), 7.1 (d, *J* = 1.7 Hz, 1 H), 7.0 (d, *J* =

1.7 Hz), 4.52 (s, 2H), 3.61 (s, 2H), 2.56 (bs, 4H), 2.34 (s, 3 H), 2.27 (s, 3H), 1.64 (p, *J* = 5.7, 4 H), 1.50 (p, *J* = 5.7, 2H); ^{13}C NMR (CD_3OD): δ 169.1, 151.3, 138.4, 137.0, 134.1, 133.5, 131.7, 131.7, 129.4, 127.9, 121.8, 72.2, 63.8, 55.0, 26.4, 24.8, 20.6, 16.5

2-Phenoxy-N-(4-(piperidin-1-ylmethyl)phenyl)acetamide (39, AL140): purchased from Chembridge; ^{1}H NMR (CD_3OD): δ 7.58 (d, *J* = 8.6 Hz, 2H), 7.34-7.29 (m, 4H), 7.05 (d, *J* = 8.6 Hz, 2 H), 7.00 (t, *J* = 7.3 Hz, 1 H), 4.66 (s, 2H), 3.48 (s, 2 H), 2.43 (bs, 4 H), 1.63-1.57 (m, 4H), 1.46 (p, *J* = 6.4 Hz, 2 H) ^{13}C NMR (CD_3OD): δ 169.4, 159.3, 138.1, 134.6, 131.4, 130.7, 122.9, 121.7, 115.9, 68.6, 64.1, 55.2, 26.4, 25.2

2-(2-Chlorophenoxy)-N-(4-(piperidin-1-ylmethyl)phenyl)acetamide (40, AL142): purchased from Chembridge; ^{1}H NMR (CD_3OD): δ 7.58 (d, *J* = 8.5 Hz, 2 H), 7.43 (dd, *J* = 7.8, 1.6 Hz, 1 H), 7.32 (d, *J* = 8.5 Hz, 2 H), 7.31-7.27 (m, 1H), 7.12 (dd, *J* = 8.4, 1.3 Hz, 1 H), 7.01-7.00 (m, 1 H), 4.75 (s, 2H), 3.48 (s, 2H), 2.43 (bs, 4 H), 1.63-1.57 (m, 4H), 1.46 (p, *J* = 6.1 Hz, 2H); ^{13}C NMR (CD_3OD): δ 168.5, 154.9, 138.1, 134.7, 131.5, 131.4, 129.3, 124.2, 124.0, 121.2, 115.9, 69.7, 64.1, 55.2, 26.4, 25.1

***N*-(4-((Diethylamino)methyl)phenyl)-2-(2,5-dimethylphenoxy)acetamide (41, AL150):** 4-nitrobenzyl bromide (200 mg, 0.94 mmol) and diethylamine (0.28 mL, 2.71 mmol) were dissolved in toluene (1 mL) and refluxed for 1 h. The resulting mixture was washed with H_2O (3×4 mL). The organic phase was extracted with HCl (3 M, 0.8 mL) and H_2O (0.8 mL), the water phases were pooled and cooled in an ice bath. After addition of NaOH (2 M) the precipitated product was filtered off and washed with ice cold water to yield 181 mg (0.87 mmol, 92%). The crude compound (206 mg, 0.99 mmol) was dissolved in EtOAc (10 mL). $SnCl_2$ (1.14 g, 6.01 mmol) was added and the reaction was stirred overnight, filtered and diluted with aqueous $NaHCO_3$ (60 ml). The mixture was extracted with CH_2Cl_2 (3×20 mL) and the combined organic phase was dried with $MgSO_4$, filtered and concentrated to a yellow oil (70 mg, 40%). The crude amine (70 mg, 0.39 mmol) was dissolved in glacial acetic acid (1.5 mL) and saturated NaOAc (2.2 mL). Chloroacetyl chloride (0.06 mL, 0.75 mmol) was added over 2 h. The reaction mixture was washed with water (4 mL) and Na_2CO_3 was added to pH 9. The water phase was extracted with CH_2Cl_2 (4×30 mL), dried with $MgSO_4$, filtered and concentrated to a yellow solid (60 mg, 60%). The resulting crude product (52 mg, 0.20 mmol), dimehtylphenol (30 mg, 0.25 mmol) and K_2CO_3 (58 mg, 0.42 mmol) were dissolved in acetone (5 mL). KI (16 mg, 0.10 mmol) was added and the reaction mixture was refluxed for 48 h, cooled to room temperature, filtered and concentrated. Purification was performed with flash column chromatography (Heptane:EtOAc 2:1→1:1, with 1% triethylamine) to yield **41** (20 mg, 33%); ^{1}H NMR (CD_3OD): δ 7.77 (d, *J* = 8.5, 2H), 7.51 (d, *J* = 8.5, 2 H), 7.04 (d, *J* = 7.6 Hz, 1 H), 6.73 (d, *J* = 7.6, 1 H), 6.72 (s, 1H), 4.68 (s, 2H), 4.31 (s, 2 H), 3.20 (q, *J* = 7.3 Hz, 4 H), 2.30 (s, 6H), 1.35 (t, *J* = 7.3 Hz, 6 H); ^{13}C NMR (CD_3OD): δ 170.0, 157.3, 140.8, 138.0, 132.8, 131.8, 126.6, 125.0, 123.3, 122.0, 113.8, 69.1, 56.7, 47.8, 21.3, 16.0, 9.1

3-(2,4-Dichlorophenyl)-N-(4-(piperidin-1-ylmethyl)phenyl)propanamide (42, AL152): 4-nitrobenzyl bromide (500 mg, 2.31 mmol) and piperidine (0.69 mL, 6.94 mmol) were dissolved in toluene (20 mL) and refluxed for 2 h. The resulting mixture was washed with H_2O (3×50 mL). The organic phase was extracted with HCl (2 M, 20 mL) and H_2O (20 mL), and the combined water phases were cooled in an ice bath. After addition of NaOH (2 M) to pH 9 the water phase was extracted with CH_2Cl_2 three times. The combined organic phases were pooled, dried over $MgSO_4$, filtered and concentrated to give the alkylated amine (456 mg, 90%) which was further used without purification. The crude compound (220.3 mg, 1.00 mmol) was dissolved in EtOAc (25 mL). $SnCl_2$ (2.75 g, 0.01 mmol) was added and the reaction was stirred overnight. Aqueous $NaHCO_3$ (50 mL) was added to pH 8 and the water phase was extracted with CH_2Cl_2 (3×50 mL). The organic phases were pooled, dried with $MgSO_4$, filtered and concentrated to give the reduced compound (165 mg, 87%). 3-(2,4-Dichlorophenyl)propanoic acid (46 mg, 0.21 mmol) was dissolved in CH_2Cl_2 (5 mL) and cooled in an ice bath. Oxalyl chloride (180 µL, 2.13 mmol) was carefully added followed by three drops of dimethylformamide. The reaction mixture was stirred for 1 h and thereafter concentrated. The resulting acid chloride (50 mg, 0.21 mmol) was dissolved in CH_2Cl_2 (4 mL), the prepared amine (48 mg, 0.25 mmol) was added together with triethylamine (100 uL, 0.72 mmol) and the reaction mixture was stirred overnight at room temperature. The mixture was washed with aq. $NaHCO_3$ (2×2 mL) and the combined organic phases were dried with $MgSO_4$, filtered, concentrated and purified with flash column chromatography (EtOAc:MeOH 5:1) to yield **42** (18 mg, 57%) as a pale yellow powder. ^{1}H NMR (CD_3OD): δ 7.66 (d, *J* = 8.5 Hz, 2 H), 7.45 (d, *J* = 8.5 Hz, 2 H), 7.44 (d, *J* = 2.3, 1H), 7.33 (d, *J* = 8.4 Hz, 1H), 7.24 (dd, *J* = 8.4, 2.3 Hz, 1 H), 4.24 (s, 2H), 3.43 (d, *J* = 12.8 Hz, 2H), 3.12 (t, *J* = 7.6 Hz, 2H), 2.94 (td, *J* = 12.8, 2.5 Hz, 2H), 2.71 (t, *J* = 7.6 Hz. 2H), 1.94 (d, *J* = 14.4 Hz, 2 H), 1.85 – 1.69 (m, 3H), 1.56 – 1.45 (m, 1H); ^{13}C NMR (CD_3OD): δ 173.0, 141.6, 138.5, 135.7, 133.9, 133.0, 132.9, 130.1, 128.4, 125.5, 121.4, 61.3, 53.8, 37.3, 29.7, 24.1, 22.7.

AChE inhibition assay

Recombinant *Homo sapiens* AChE was expressed according to previously described methods [6,7]. The enzymatic activity was measured using the Ellman assay [8] adapted to a 96-well format. The final assay volume was 200 μL and all measurements were performed in 0.2 mM 5,5'-Dithiobis(2-nitrobenzoic acid) and 1 mM acethylthiocholine iodide in 0.1 M phosphate buffer pH 8.0. All measurements were performed in triplicates and the data was fitted to a (log)inhibitor vs. response four parameters equation using GraphPad Prism (5.0). The obtained IC_{50}-values were recalculated to pIC_{50} according to pIC_{50}=-log(IC_{50}) in molar (M) concentration.

Permutation tests

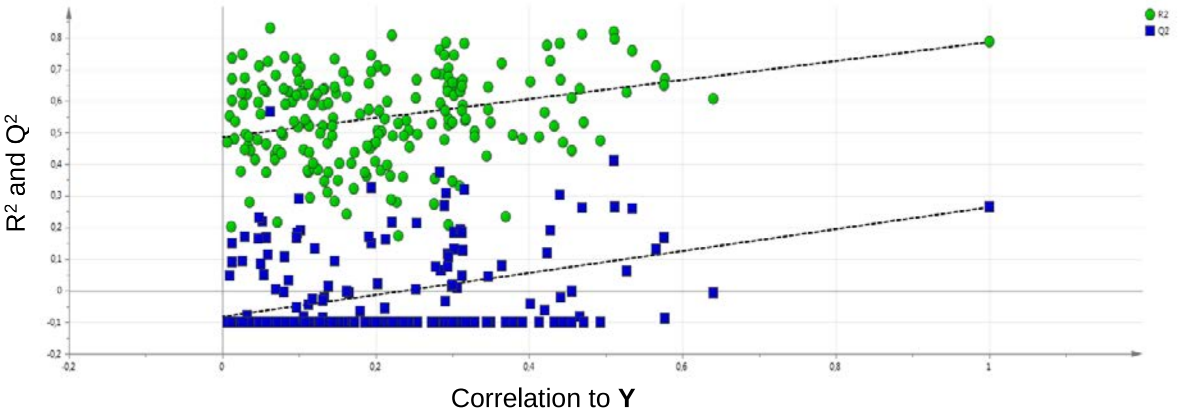

Figure S14 a)Permutation test from the qualitative PLS-based SAR model

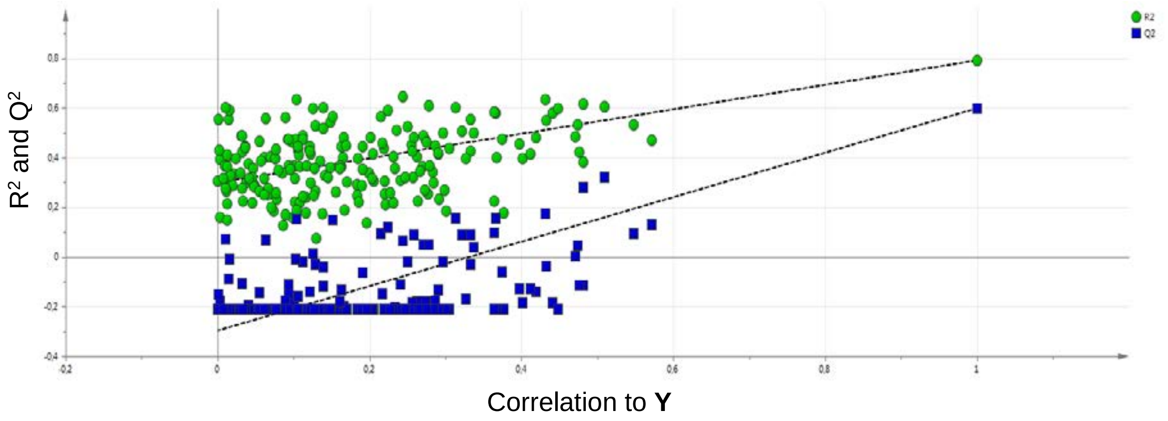

Figure S14 b) permutation test of quantitative PLS-based QSAR-model

Test sets Set1, Set2, and Set3 molecules and inhibition data

Table S15. Test Set1, Set2, and Set3 [9] names, molecules and AChE inhibition data

ID	Name	Test Set	Structure	IC_{50} (μM)	Nearest Neighbor in training set[a]
**27**	AL053	1	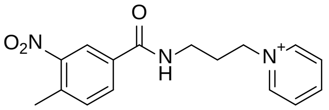	4.9	6
**28**	AL054	1	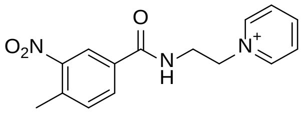	10	6
**29**	AL055	1	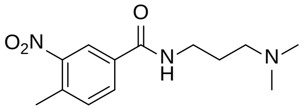	19	6
**30**	AL058	1	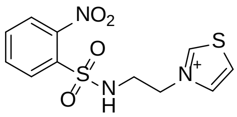	12	4
**31**	AL129	1	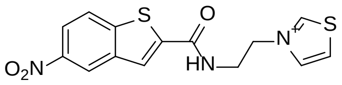	2.6	14
**36**	AL137	2	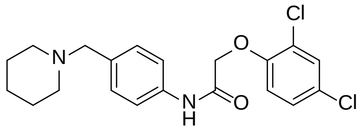	0.3	12
**37**	AL138	2	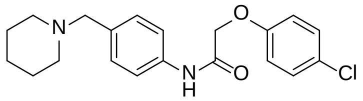	0.6	18
**38**	AL139	2	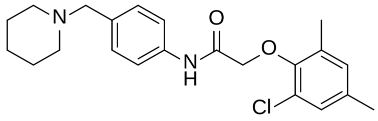	1.3	18
**39**	AL140	2	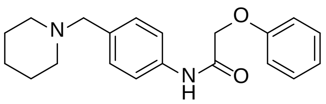	1.3	18
**40**	AL142	2	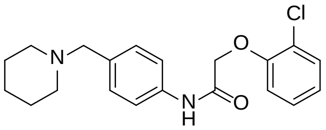	0.6	18
**41**	Al150	2	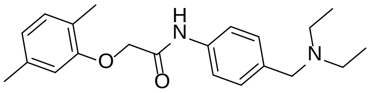	8.1	12

ID	Name	Test Set	Structure	IC_{50} (μM)	Nearest Neighbor in training set[a]
**42**	AL152	2	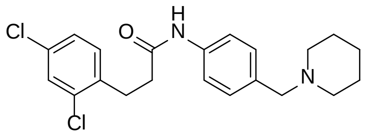	0.7	18
**43**	AL023	3	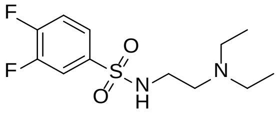	51	4
**44**	AL024	3	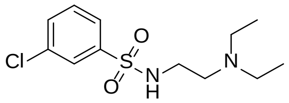	19	1
**45**	AL025	3	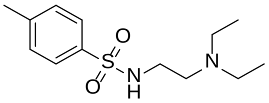	135	4
**46**	AL026	3	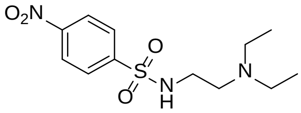	162	4
**47**	AL027	3	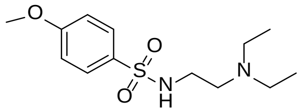	118	1
**48**	AL028	3	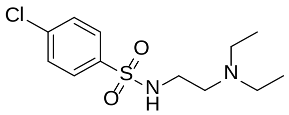	91	4
**49**	AL029	3	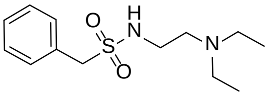	134	1
**50**	AL030	3	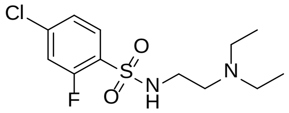	94	4
**51**	AL031	3	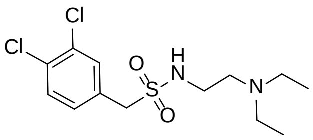	6.8	1
**52**	AL032	3	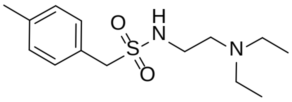	51	1
**53**	AL033	3	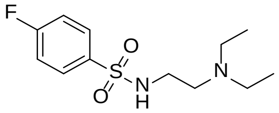	65	4

ID	Name	Test Set	Structure	IC_{50} (μM)	Nearest Neighbor in training set[a]
**54**	AL034	3	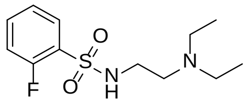	42	4
**55**	AL036	3	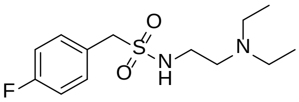	42	1
**56**	AL037	3	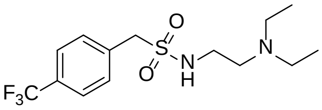	17	17
**57**	AL038	3	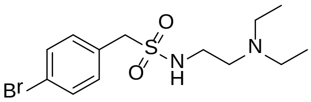	11	1
**58**	AL039	3	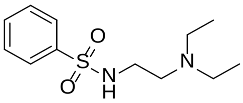	33	1
**59**	AL040	3	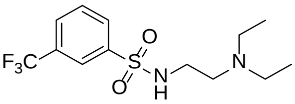	24	4
**60**	AL041	3	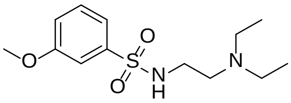	10	1
**61**	AL144	3	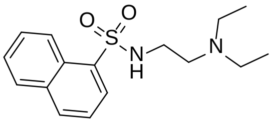	38	4
**62**	Al151	3	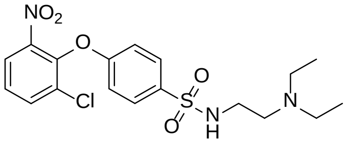	0.7	8

[a] Nearest neighbor in the training set selected by chemists

Reference model regressions for logP, TPSA and vdW area

a)

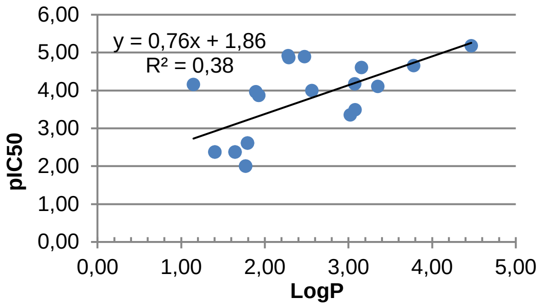

b)

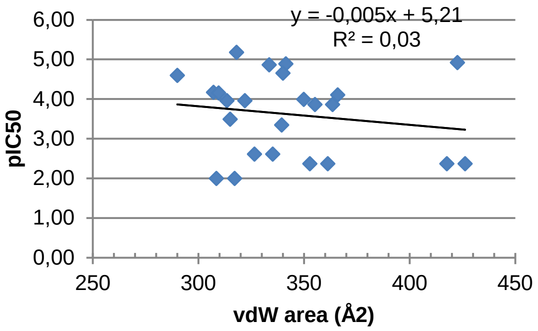

c)

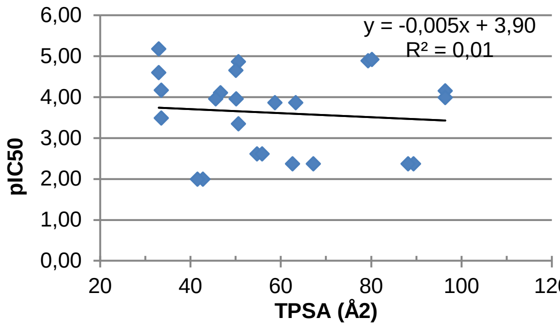

Figure S18. Reference model regressions for a) logP, b) vdW area, and c) TPSA.

Statistical analysis of QSAR model and reference models

Table S19. Statistics test including, Anderson-Darling, F-test, t-test, Kolmogorov-Smirnov and Mann-Whitney, comparing the predicted pIC_{50} values from corresponding model to the measured pIC_{50}.

Test/ Model	**Set1**			**Set2**			**Set3**				**Set4**		
	AD	F	t	AD	F	t	AD	F	t	KS	AD	KS	MW
	norm. distr.	F_{crit}: 6.39	t_{crit}: 2.78	norm. distr.	F_{crit}: 4.28	t_{crit}: 2.45	norm. distr.	F_{crit}: 2.17	t_{crit}: 2.09	p-value	norm. distr.	p-value	p-value
QSAR	Yes	**1.87**	**1.71**	Yes	**2.25**	**2.17**	Yes	3.5	**-**	**0.275**	No	**0.518**	**0.330**
LogP	Yes	**2.22**	11.53	Yes	**1.16**	4.24	Yes	**1.7**	11.71	0.000	No	0.000	0.000
vdw	Yes	69.90	-	Yes	31.0	-	No	-	-	0.000	No	0.000	0.000
TPSA	No	-	-	No	-	-	No	-	-	0.000	No	0.000	0.000
PLS	Yes	**3.08**	6.29	Yes	**1.26**	2.84	No	-	-	0.000	No	0.000	0.000
Nearest Neighbor	No	-	-	No	-	-	No	-	-	**0.275**	No	0.007	0.013

The predicted pIC_{50} resulting of all four test sets from the QSAR model and reference models were tested for the probability that they were drawn from a normal distribution using the Anderson-Darling (AD) test [10] at a confidence limit of 95% (p=0.05) implemented in Excel [11,12]. Average and median pIC_{50} values were obviously not normally distributed and was excluded from the significance testing. The AD-test result are given in the table above and show that the predicted pIC_{50} for **Set1-2** resulting from the QSAR model and reference models LogP, vdW and PLS were normally distributed. So were the predictions for **Set3** from the QSAR and LogP models, but no predictions for **Set4**. Predicted pIC_{50} were compared to measured pIC_{50} values *via* a one-tailed F-test (comparing variances) and a paired students t-test (comparing means) and critical values based on prediction molecules minus one (n-1) degrees of freedom. If $F < F_{crit}$ or $t < t_{crit}$ there is a no difference between the models' predictions and the measured pIC_{50} in terms of variance and median, respectively. The conclusion regarding pIC_{50} based on both F- and t-tests was that the QSAR-models' predictions was generally statistically not different from the measured pIC_{50} (calculated F or t smaller critical value), while the reference models were significantly different from measured pIC_{50} (calculated F or t was larger critical value). Non-parametric Kolmogorov-Smirnov (KS) [13,14] and Mann-Whitney (MW) U-test [15,16] tests were performed on **Set3-4** (**Set1** and **Set2** contained too few data points), which generally had non-normally distributed predictions and the results showed that the only significant similarity was between the QSAR models' prediction and the measured pIC_{50} for **Set3-4** and for Nearest neighbor for **Set3**. The reference models average and median was tested with the KS and MW tests and resulted in a p of 0.000, indicating that average and median are insufficient predictors of pIC_{50}.

References

1. Linusson A, Elofsson M, Andersson IE, Dahlgren MK (2010) Statistical molecular design of balanced compound libraries for QSAR modeling. Curr Med Chem 17: 2001-2016
2. St. John RC, Draper NR (1975) D-Optimality for regression designs: a review. Technometrics 17: 15-23
3. Wang Y-X, Zhao J, Sun X-Q, Wang C-J (2006) Synthesis, interaction with DNA and bioactivity of N-piperazinoalkylamide. Youji Huaxue 26: 1066-1072
4. Deetlefs M, Seddon KR (2003) Improved preparations of ionic liquids using microwave irradiation. Green Chem 5: 181-186
5. Lewis JJ, Martin-Smith M, Muir TC, Nanjappa SN, Reid ST (1963) Benzo[b]Thiophene derivatives . IV. The preparation and pharmacological activity of compounds related to serotonin and gramine. J Med Chem 6: 711-716
6. Ekström F, Pang YP, Boman M, Artursson E, Akfur C, Börjegren S (2006) Crystal structures of acetylcholinesterase in complex with HI-6, Ortho-7 and obidoxime: Structural basis for differences in the ability to reactivate tabun conjugates. Biochem Pharmacol 72: 597-607
7. Artursson E, Akfur C, Hörnberg A, Worek F, Ekström F (2009) Reactivation of tabun-hAChE investigated by structurally analogous oximes and mutagenesis. Toxicology 265: 108-114
8. Ellman GL, Courtney KD, Andres V, Featherstone RM (1961) A new and rapid colorimetric determination of acetylcholinesterase activity. Biochem Pharm 7: 88-95
9. Andersson CD, Forsgren N, Akfur C, Allgardsson A, Berg L, Engdahl C, Qian WX, Ekström F, Linusson A (2013) Divergent structure-activity relationships of structurally similar acetylcholinesterase inhibitors. J Med Chem 56: 7615-7624
10. Anderson TW, Darling DA (1952) Asymptotic theory of certain goodness of fit criteria based on stochastic processes. Ann Math Stat 23: 193-212
11. Excel (2013) Microsoft Excel Microsoft Corporation, Redmond, Washington, US.
12. Otto KN (2005) Normality Test Calculator.xls. http://www.kevinotto.com/RSS/templates/Anderson-Darling. Accessed 2014
13. Massey FJ (1951) The Kolmogorov-Smirnov test for goodness of fit. J Am Stat Assoc 46: 68-78
14. Kirkman TW (2014) Statistics to Use. http://www.physics.csbsju.edu/stats/. Accessed 28 Jan 2014
15. Mann HB, Whitney DR (1947) On a test of whether one of 2 random variables is stochastically larger than the other. Ann Math Stat 18: 50-60
16. Stangroom J (2014) Social Science Statistics. http://www.socscistatistics.com. Accessed 27 Jan 2014
