# Supplementary material for: Benefits of statistical molecular design, covariance analysis, and reference models in QSAR: a case study on acetylcholinesterase
Source: J Comput Aided Mol Des. 2014 Oct 29;29(3):199–215. doi: 10.1007/s10822-014-9808-1 (PMC4330465; doi:10.1007/s10822-014-9808-1)

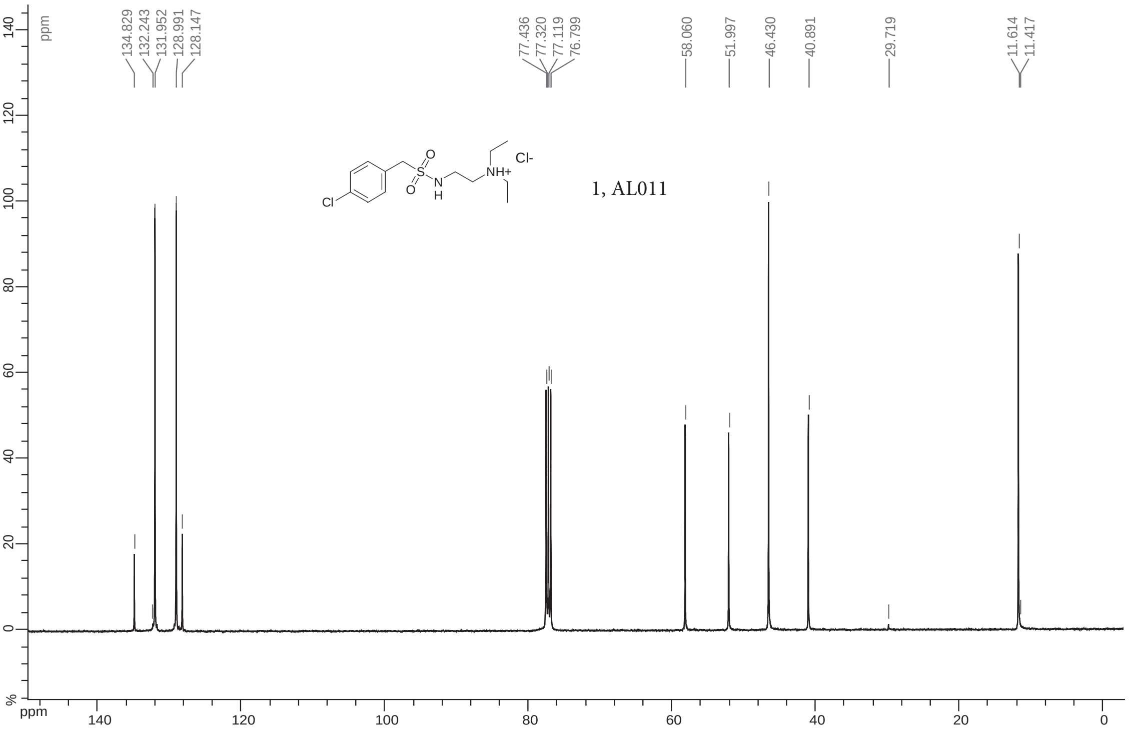
134.829
132.243
131.952
128.991
128.147
77.436
77.320
77.119
76.799
58.060
51.997
46.430
40.891
29.719
11.614
11.417
1, AL011
Cl-
NH+
ppm
%

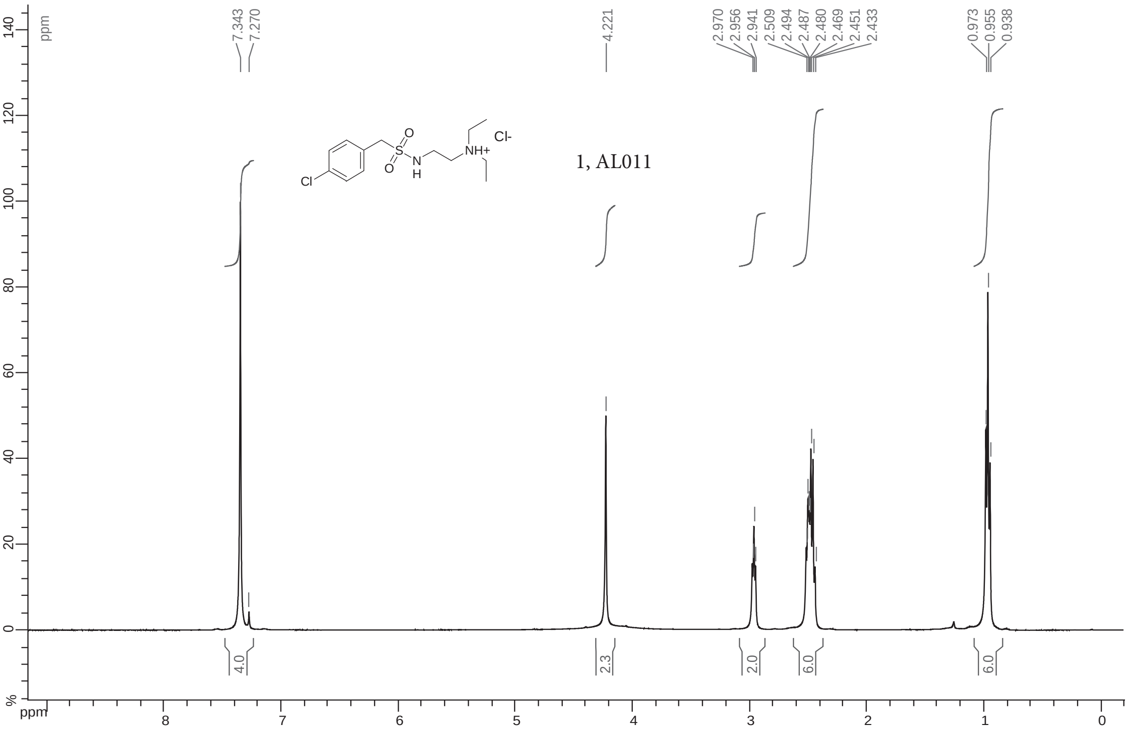
7.343
7.270
4.221
2.970
2.956
2.941
2.509
2.494
2.487
2.480
2.469
2.451
2.433
0.973
0.955
0.938
1, AL011
Cl-
NH+
4.0
2.3
2.0
6.0
6.0
ppm
%

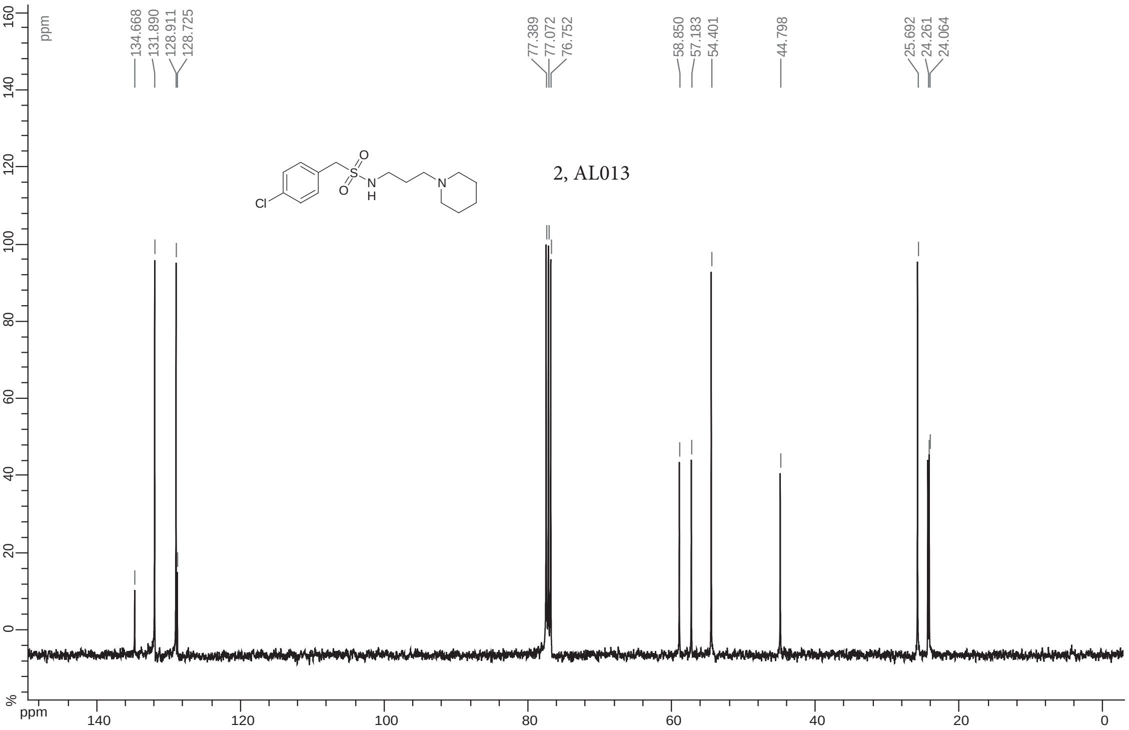
134.668
131.890
128.911
128.725
77.389
77.072
76.752
58.850
57.183
54.401
44.798
25.692
24.261
24.064
2, AL013
Cl
S
O
O
N
H
N
ppm
%
160
140
120
100
80
60
40
20
0
140
120
100
80
60
40
20
0

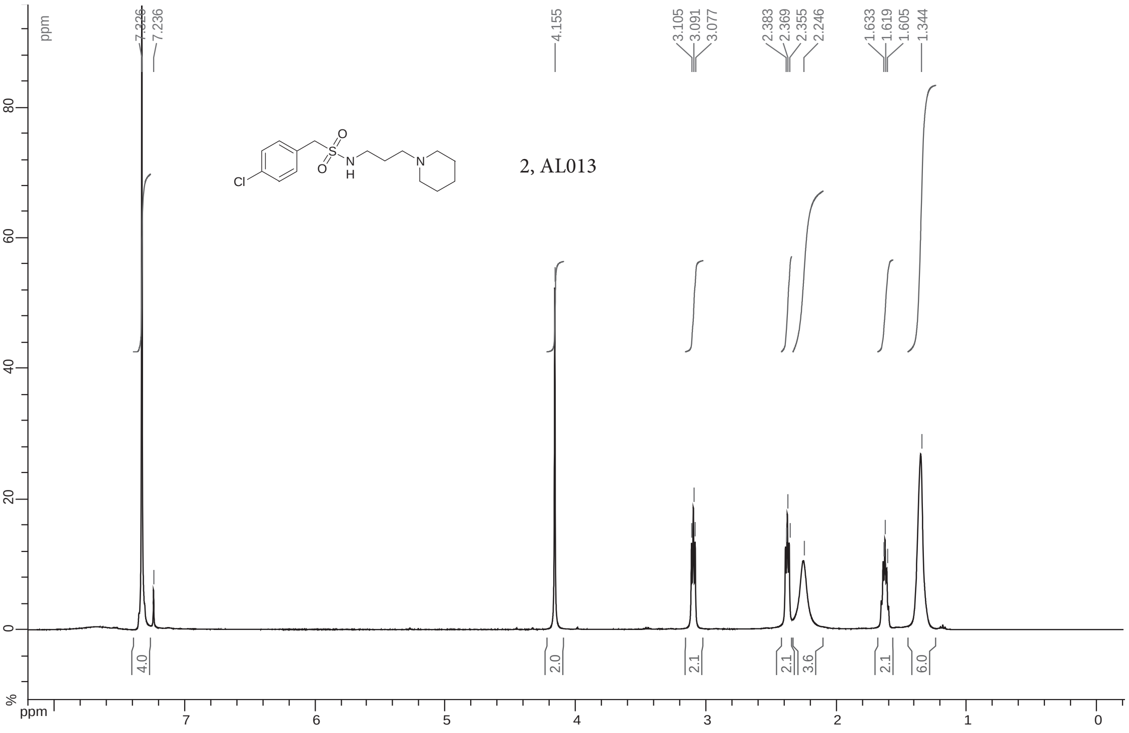
7.326
7.236
4.155
3.105
3.091
3.077
2.383
2.369
2.355
2.246
1.633
1.619
1.605
1.344
2, AL013
Cl
S
O
O
N
H
N
ppm
%
80
60
40
20
0
4.0
2.0
2.1
2.1
3.6
2.1
6.0
7
6
5
4
3
2
1
0

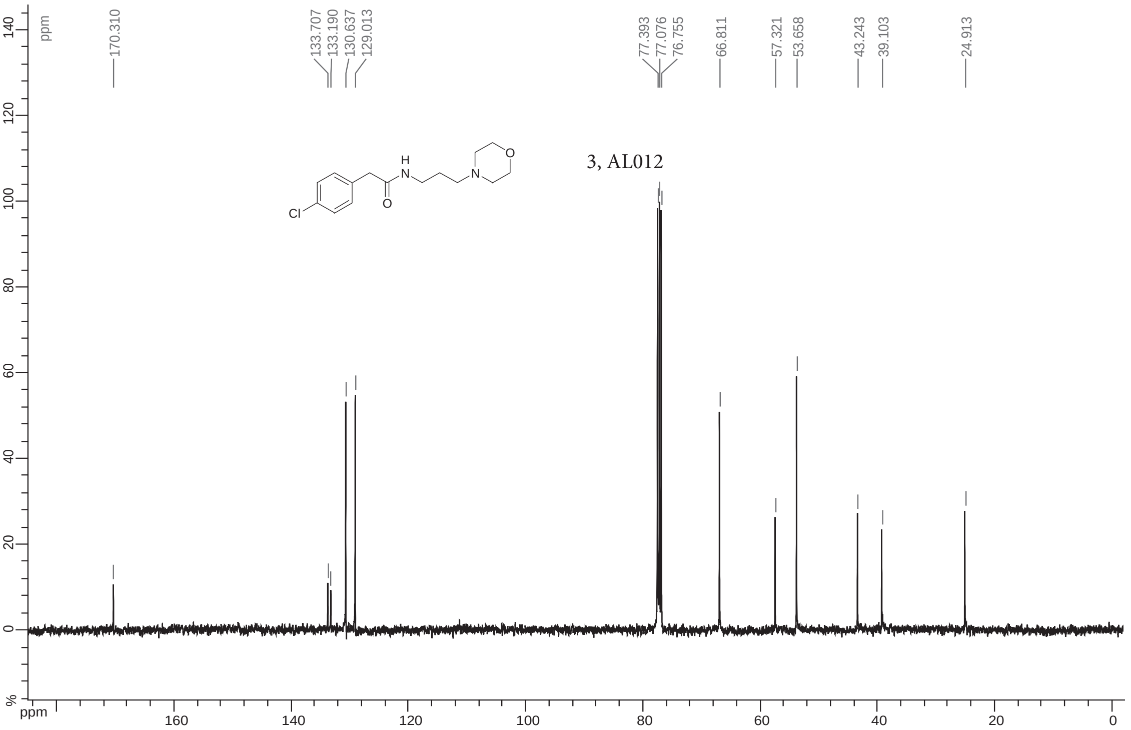
3, AL012
170.310
133.707
133.190
130.637
129.013
77.393
77.076
76.755
66.811
57.321
53.658
43.243
39.103
24.913
ppm
Cl
H
N
O
%

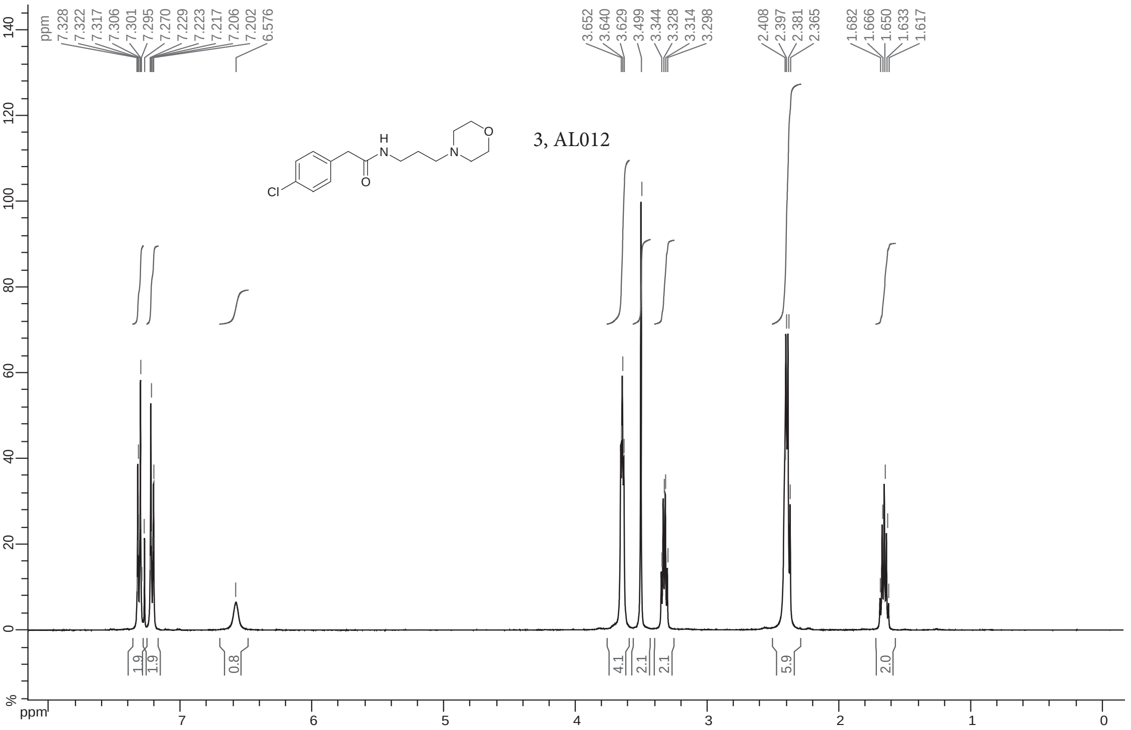
3, AL012
7.328
7.322
7.317
7.306
7.301
7.295
7.270
7.229
7.223
7.217
7.206
7.202
6.576
3.652
3.640
3.629
3.499
3.344
3.328
3.314
3.298
2.408
2.397
2.381
2.365
1.682
1.666
1.650
1.633
1.617
1.9
1.9
0.8
4.1
2.1
2.1
5.9
2.0
ppm
Cl
H
N
O
%

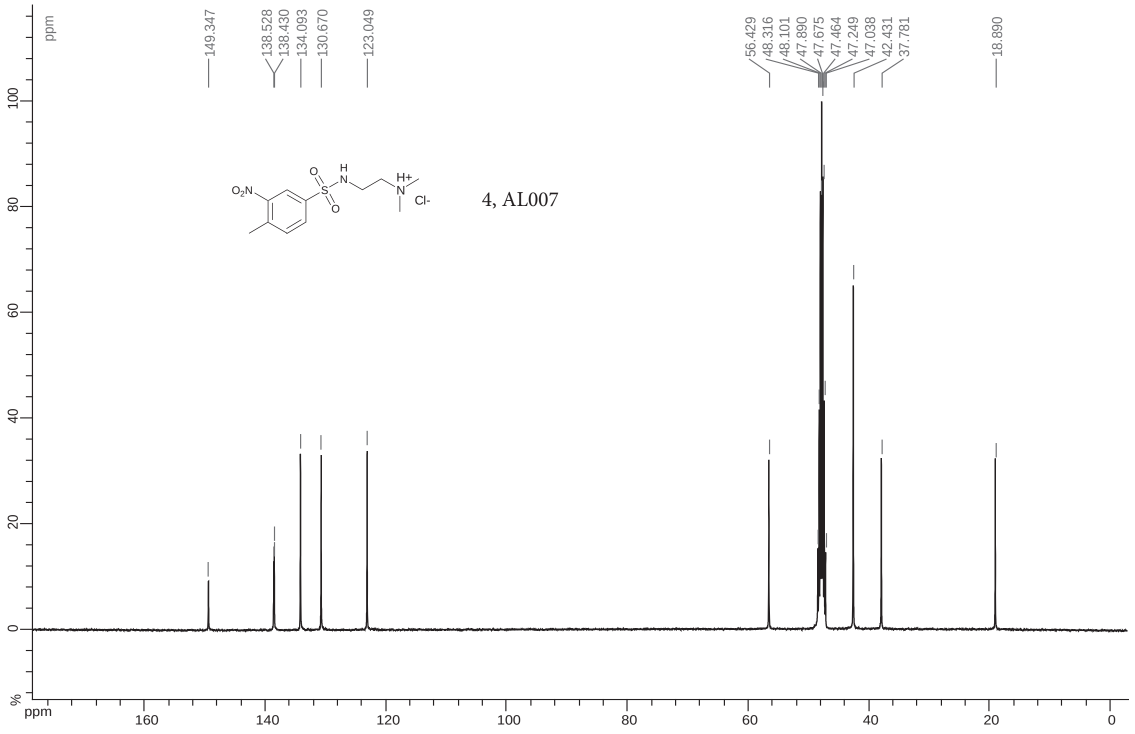
4, AL007
ppm
149.347
138.528
138.430
134.093
130.670
123.049
56.429
48.316
48.101
47.890
47.675
47.464
47.249
47.038
42.431
37.781
18.890
Cl-
H+

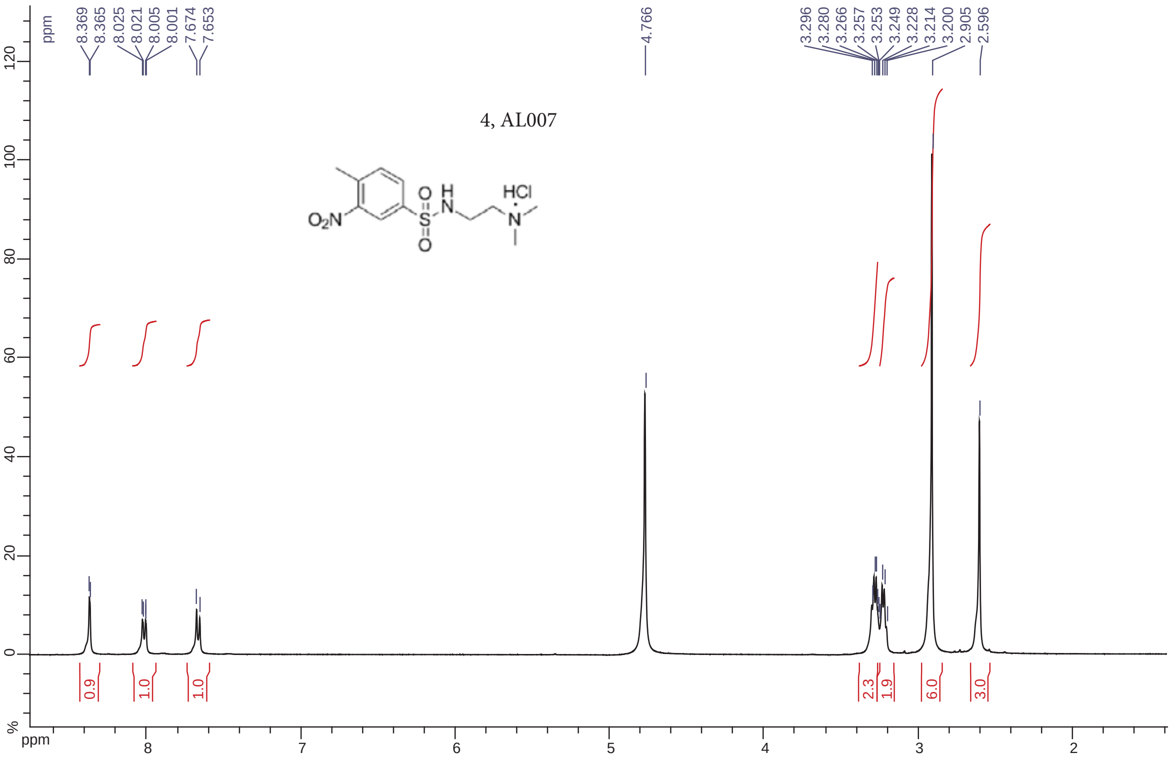
4, AL007
HCl
ppm
8.369
8.365
8.025
8.021
8.005
8.001
7.674
7.653
4.766
3.296
3.280
3.266
3.257
3.253
3.249
3.228
3.214
3.200
2.905
2.596
0.9
1.0
1.0
2.3
1.9
6.0
3.0

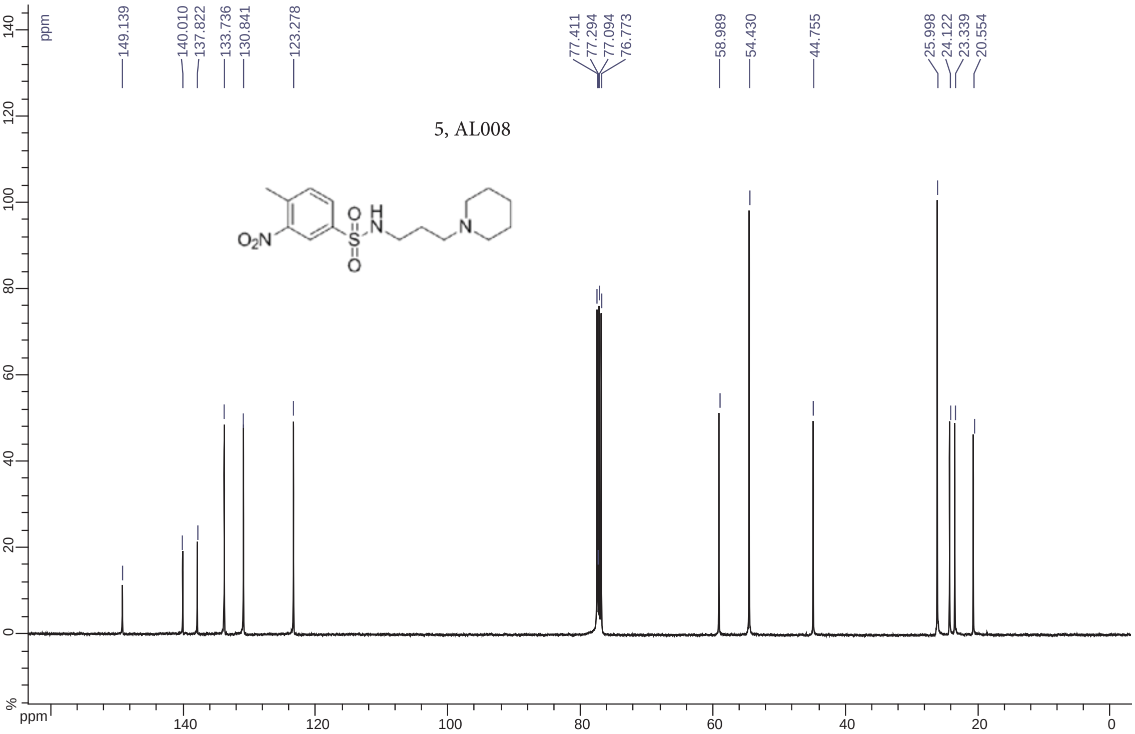
5, AL008
ppm
149.139
140.010
137.822
133.736
130.841
123.278
77.411
77.294
77.094
76.773
58.989
54.430
44.755
25.998
24.122
23.339
20.554
%
140 120 100 80 60 40 20 0

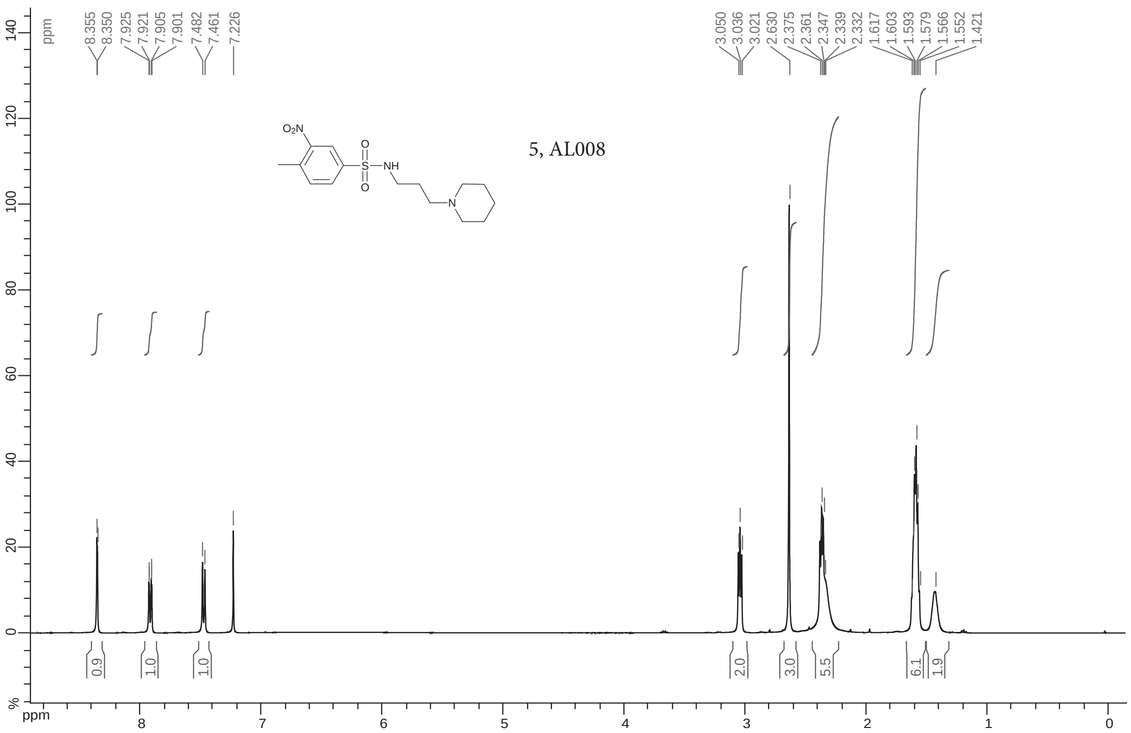
5, AL008
ppm
8.355
8.350
7.925
7.921
7.905
7.901
7.482
7.461
7.226
3.050
3.036
3.021
2.630
2.375
2.361
2.347
2.339
2.332
1.617
1.603
1.593
1.579
1.566
1.552
1.421
0.9
1.0
1.0
2.0
3.0
5.5
6.1
1.9
%
8 7 6 5 4 3 2 1 0

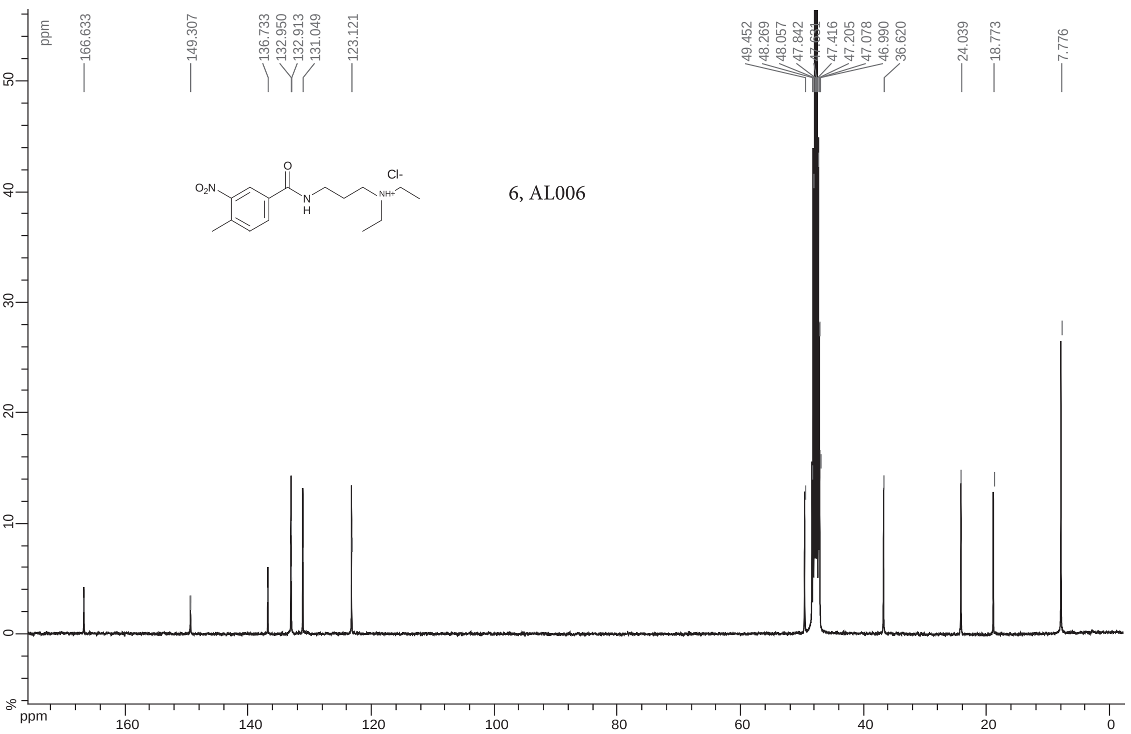
ppm
166.633
149.307
136.733
132.950
132.913
131.049
123.121
49.452
48.269
48.057
47.842
47.416
47.205
47.078
46.990
36.620
24.039
18.773
7.776
Cl-
6, AL006
%
ppm
160
140
120
100
80
60
40
20
0

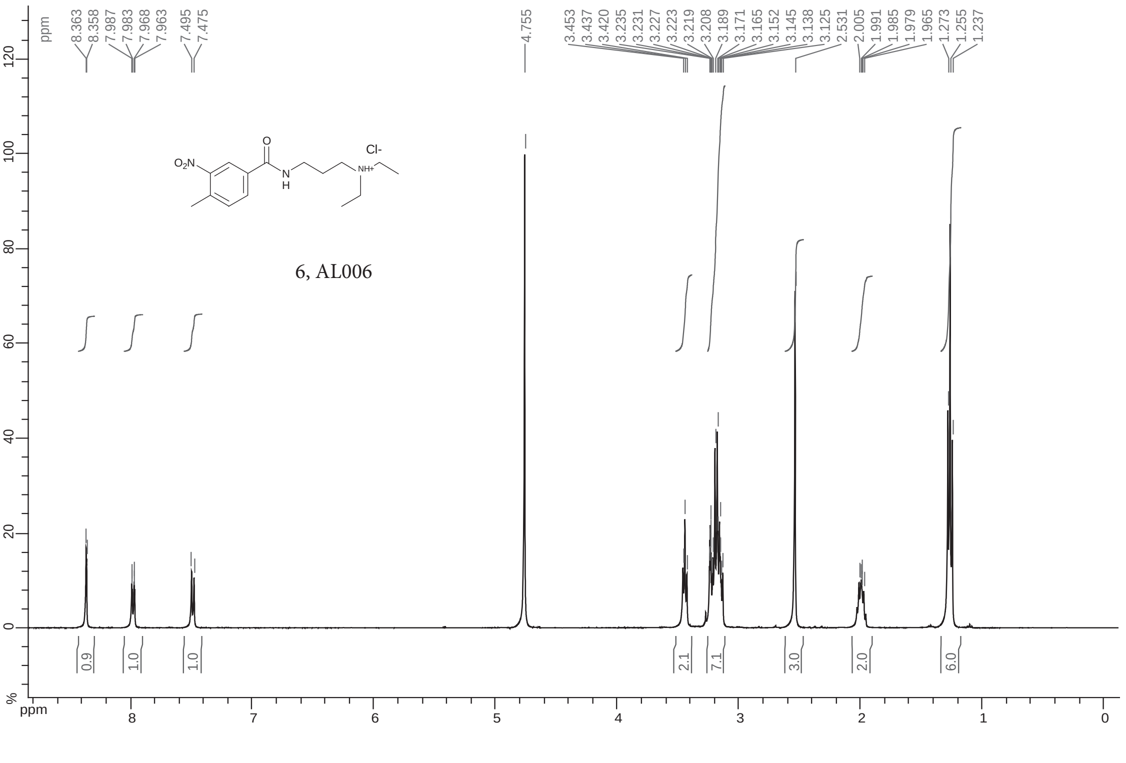
ppm
8.363
8.358
7.987
7.983
7.968
7.963
7.495
7.475
4.755
3.453
3.437
3.420
3.235
3.231
3.227
3.223
3.219
3.208
3.189
3.171
3.165
3.152
3.145
3.138
3.125
2.531
2.005
1.991
1.985
1.979
1.965
1.273
1.255
1.237
Cl-
6, AL006
0.9
1.0
1.0
2.1
7.1
3.0
2.0
6.0
%
ppm
8
7
6
5
4
3
2
1
0

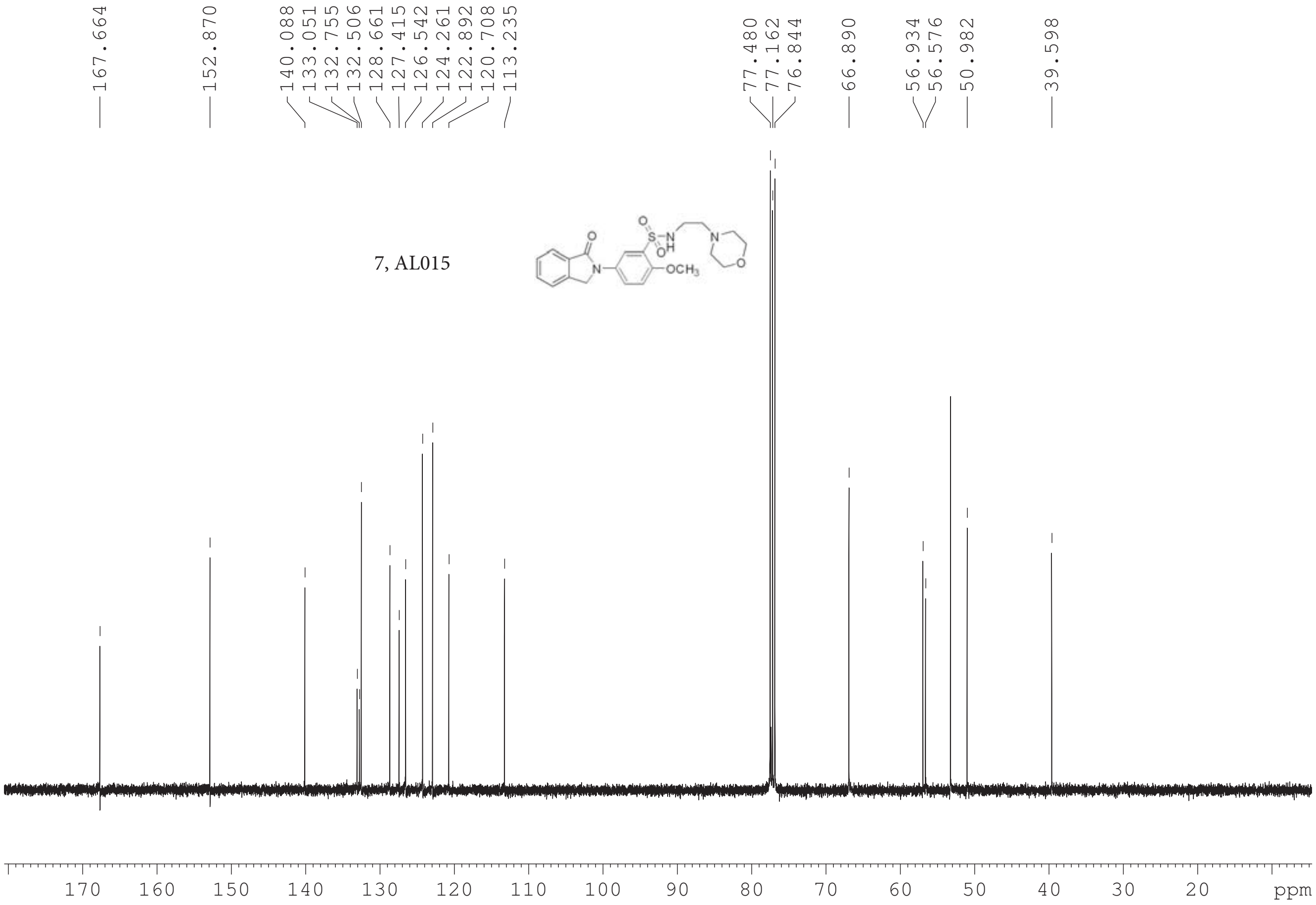
167.664
152.870
140.088
133.051
132.755
132.506
128.661
127.415
126.542
124.261
122.892
120.708
113.235
77.480
77.162
76.844
66.890
56.934
56.576
50.982
39.598
7, AL015
OCH3
170 160 150 140 130 120 110 100 90 80 70 60 50 40 30 20 ppm

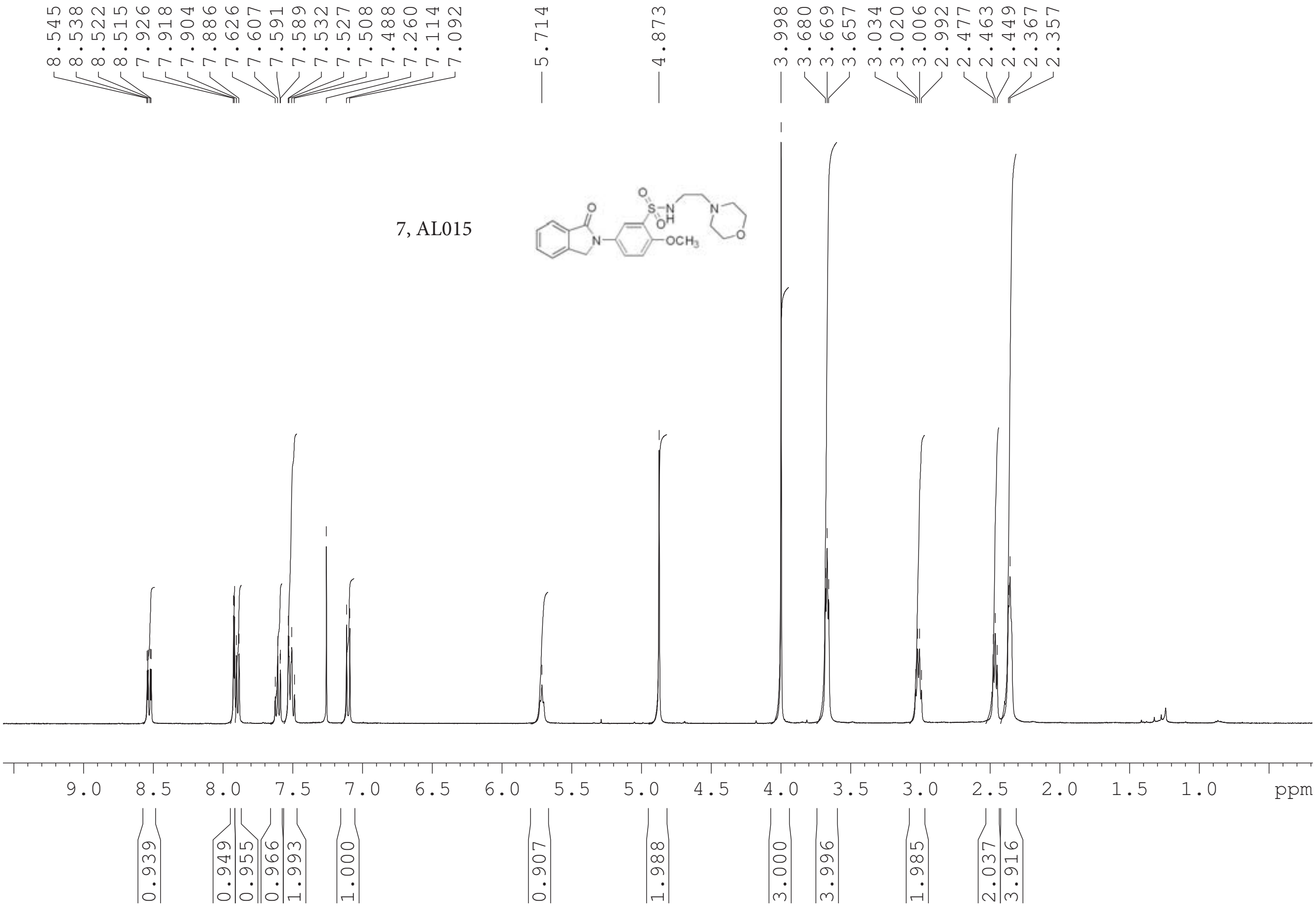
8.545
8.538
8.522
8.515
7.926
7.918
7.904
7.886
7.626
7.607
7.591
7.589
7.532
7.527
7.508
7.488
7.260
7.114
7.092
5.714
4.873
3.998
3.680
3.669
3.657
3.034
3.020
3.006
2.992
2.477
2.463
2.449
2.367
2.357
7, AL015
OCH3
9.0 8.5 8.0 7.5 7.0 6.5 6.0 5.5 5.0 4.5 4.0 3.5 3.0 2.5 2.0 1.5 1.0 ppm
0.939
0.949
0.955
0.966
1.993
1.000
0.907
1.988
3.000
3.996
1.985
2.037
3.916

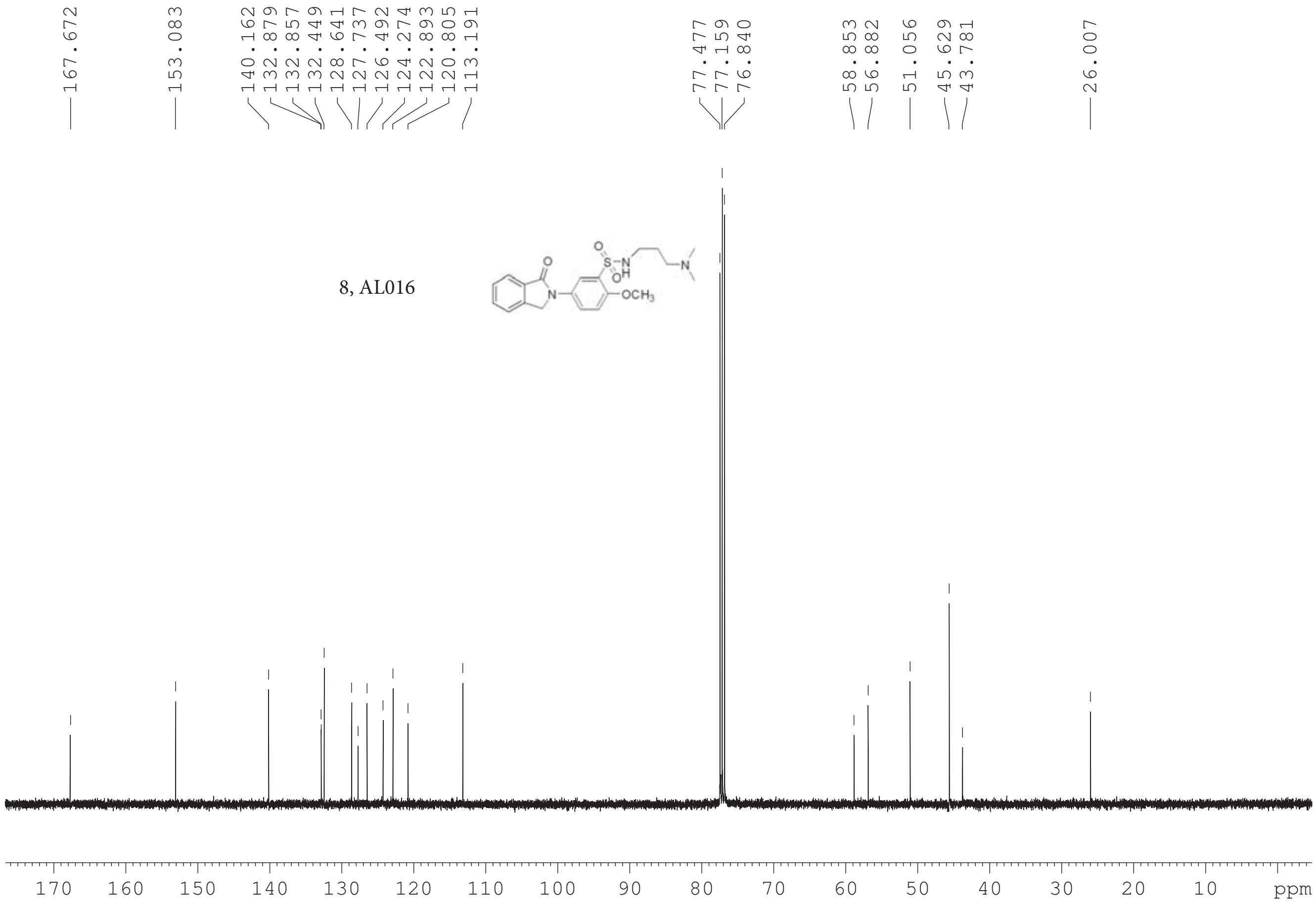
167.672
153.083
140.162
132.879
132.857
132.449
128.641
127.737
126.492
124.274
122.893
120.805
113.191
77.477
77.159
76.840
58.853
56.882
51.056
45.629
43.781
26.007
8, AL016
170 160 150 140 130 120 110 100 90 80 70 60 50 40 30 20 10 ppm

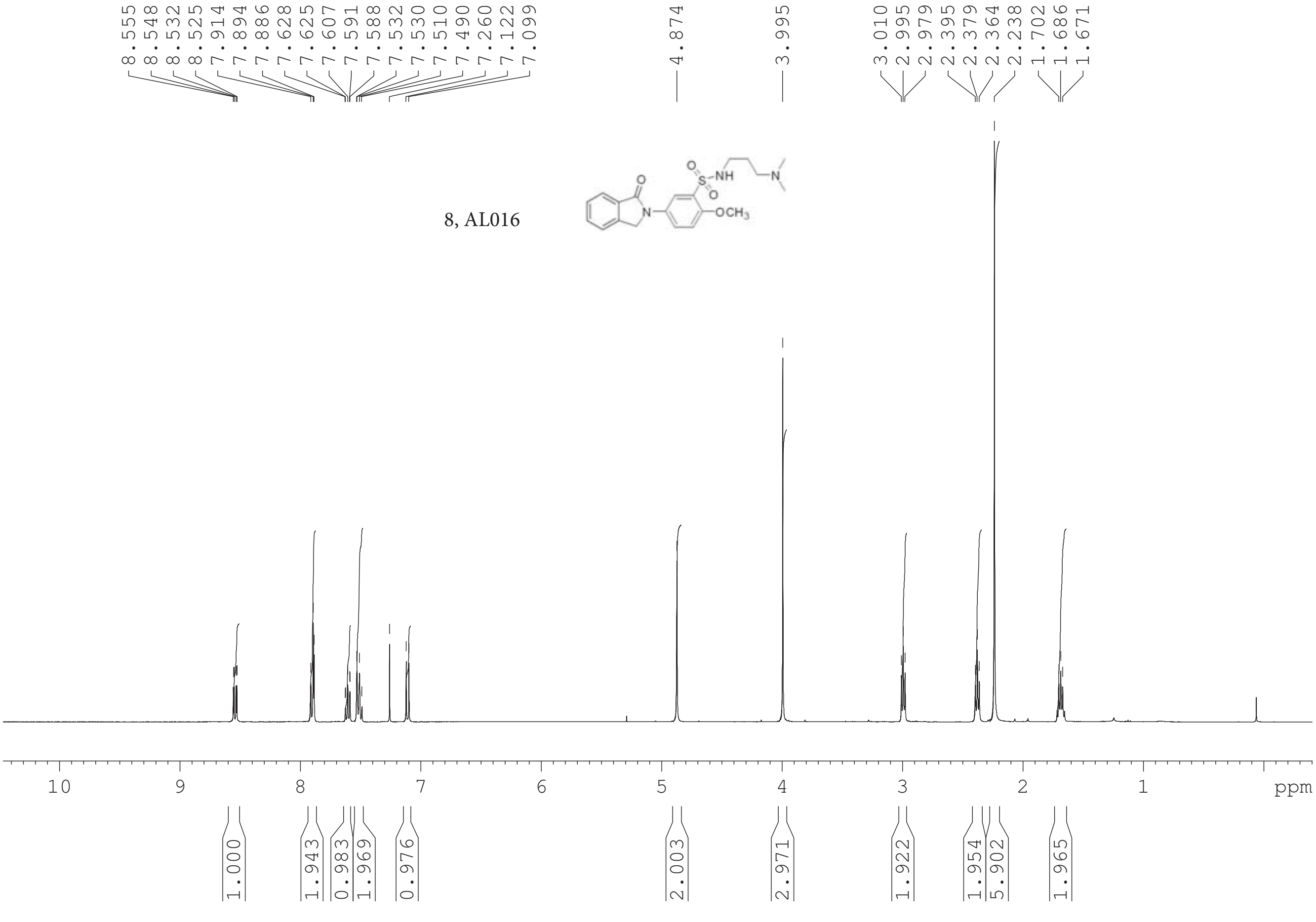
8.555
8.548
8.532
8.525
7.914
7.894
7.886
7.628
7.625
7.607
7.591
7.588
7.532
7.530
7.510
7.490
7.260
7.122
7.099
4.874
3.995
3.010
2.995
2.979
2.395
2.379
2.364
2.238
1.702
1.686
1.671
8, AL016
10 9 8 7 6 5 4 3 2 1 ppm
1.000
1.943
0.983
1.969
0.976
2.003
2.971
1.922
1.954
5.902
1.965

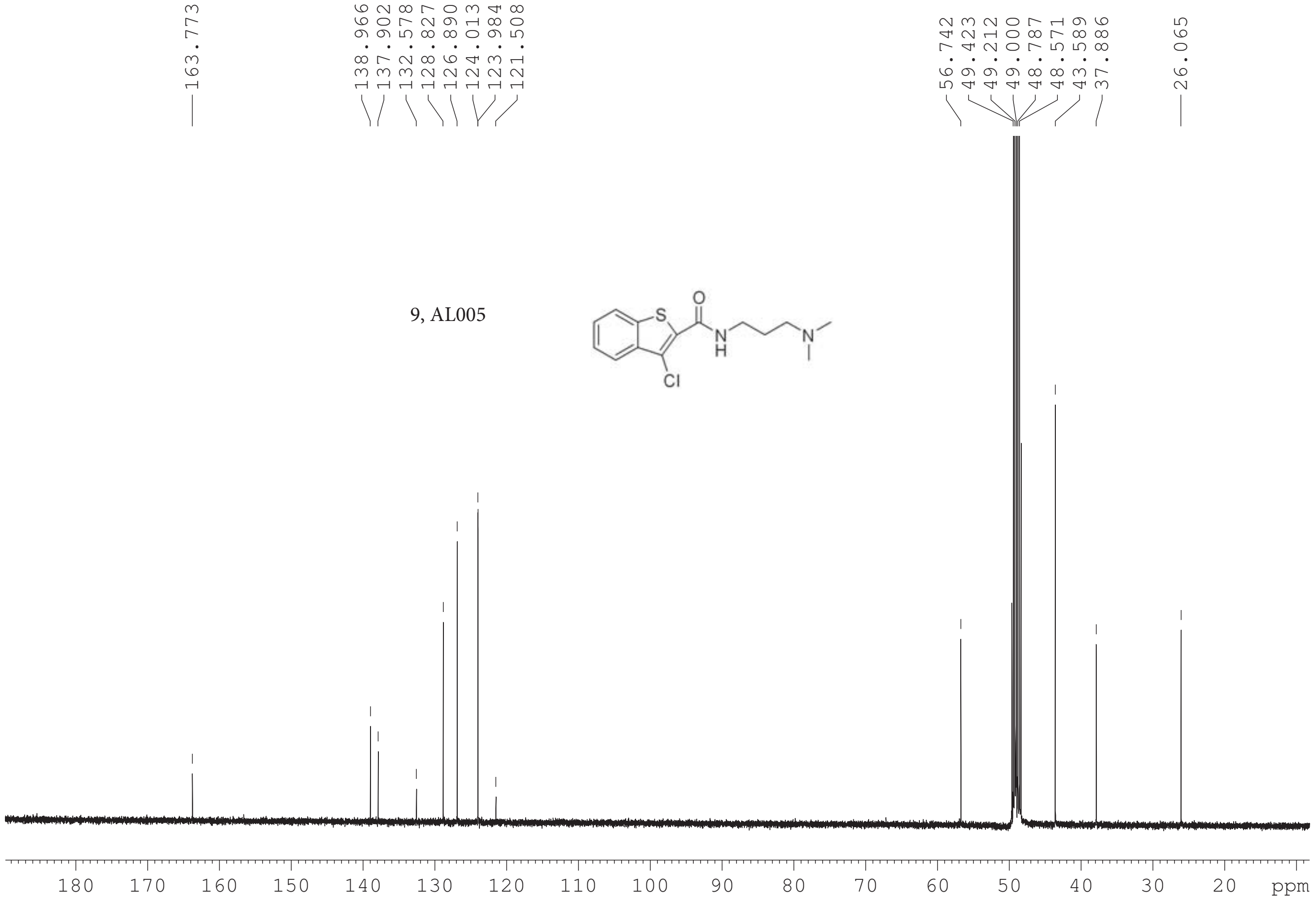
163.773
138.966
137.902
132.578
128.827
126.890
124.013
123.984
121.508
56.742
49.423
49.212
49.000
48.787
48.571
43.589
37.886
26.065
9, AL005
S
O
N
H
N
Cl
180 170 160 150 140 130 120 110 100 90 80 70 60 50 40 30 20 ppm

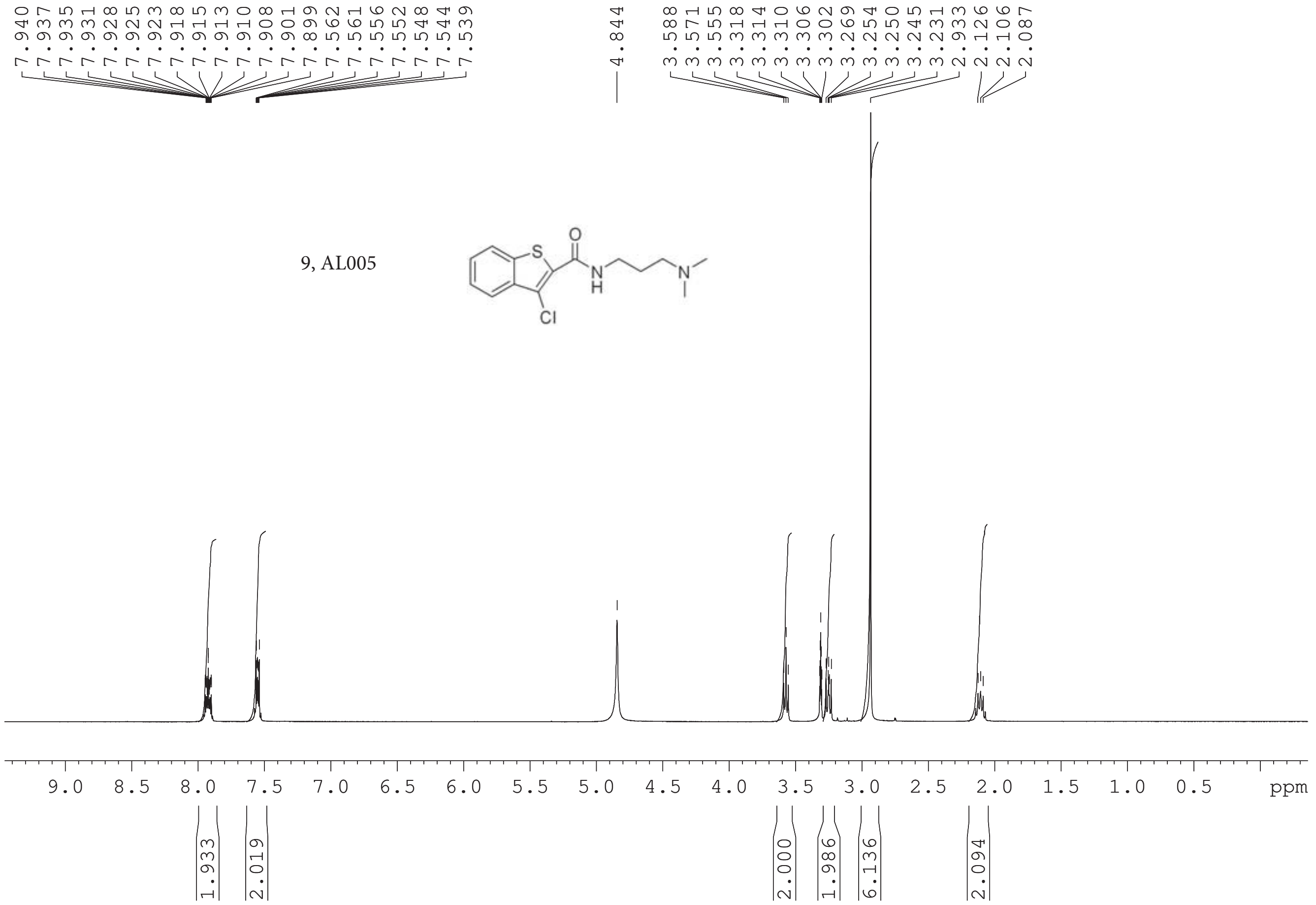
7.940
7.937
7.935
7.931
7.928
7.925
7.923
7.918
7.915
7.913
7.910
7.908
7.901
7.899
7.562
7.561
7.556
7.552
7.548
7.544
7.539
4.844
3.588
3.571
3.555
3.318
3.314
3.310
3.306
3.302
3.269
3.254
3.250
3.245
3.231
2.933
2.126
2.106
2.087
9, AL005
S
O
N
H
N
Cl
9.0 8.5 8.0 7.5 7.0 6.5 6.0 5.5 5.0 4.5 4.0 3.5 3.0 2.5 2.0 1.5 1.0 0.5 ppm
1.933
2.019
2.000
1.986
6.136
2.094

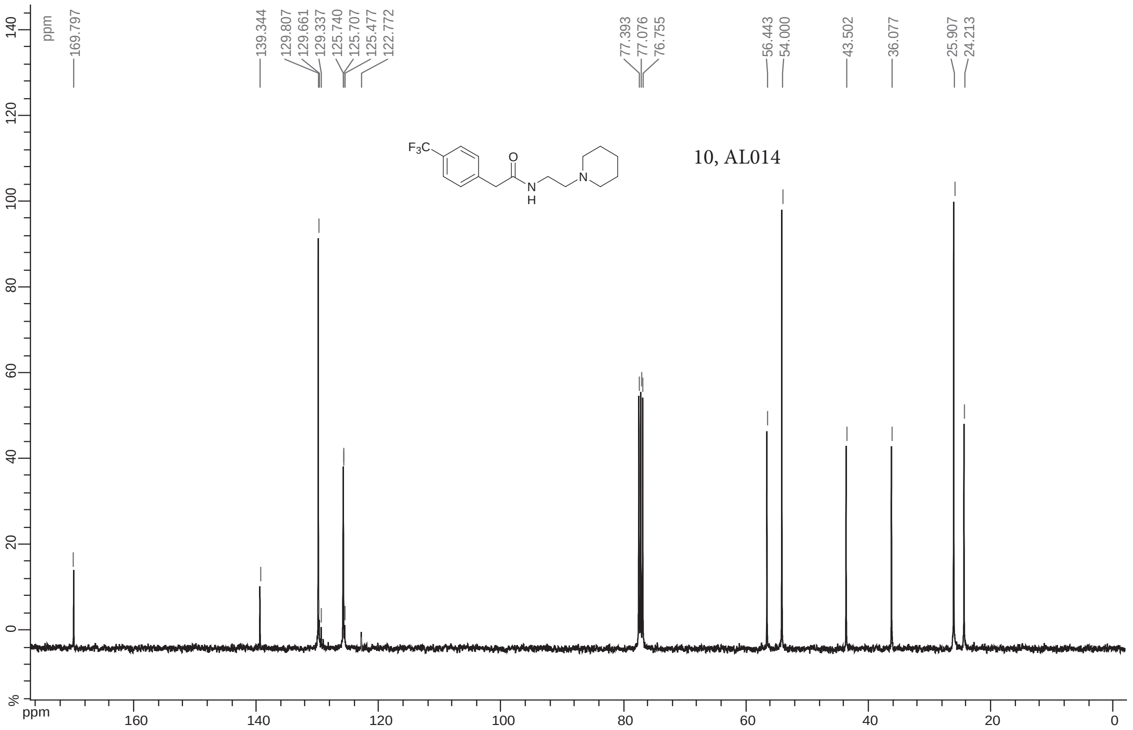
10, AL014
ppm
169.797
139.344
129.807
129.661
129.337
125.740
125.707
125.477
122.772
77.393
77.076
76.755
56.443
54.000
43.502
36.077
25.907
24.213
F3C
O
N
H
N
%

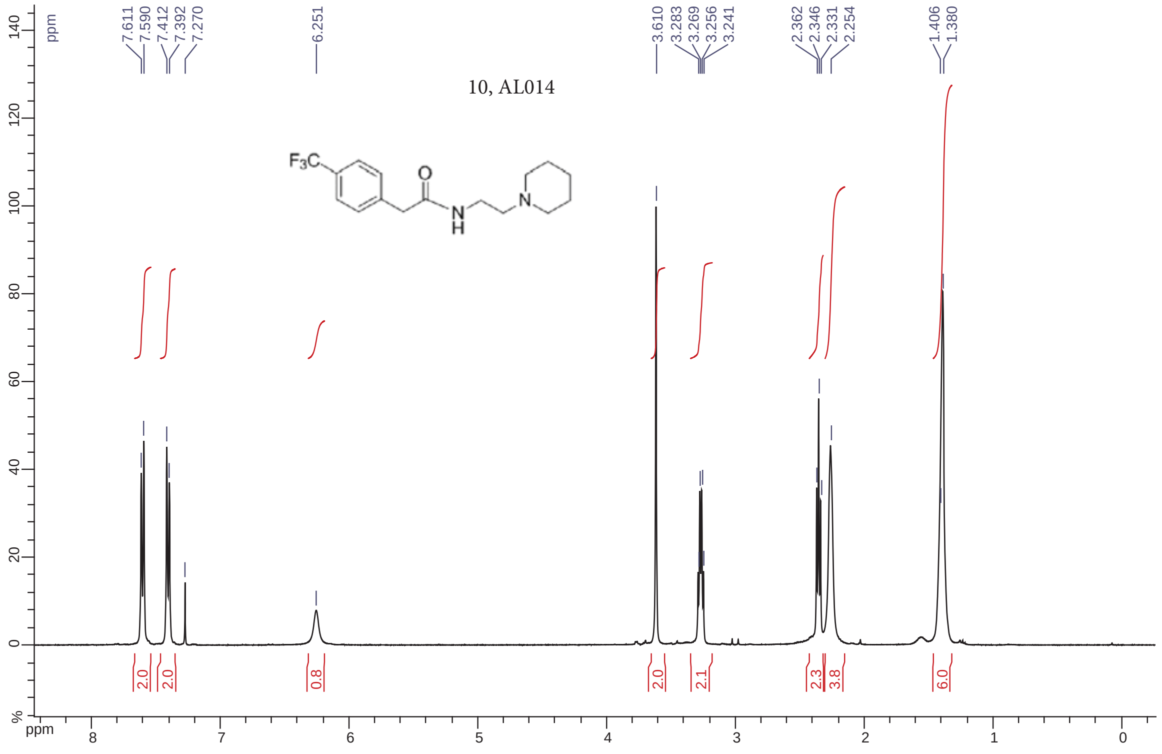
10, AL014
ppm
7.611
7.590
7.412
7.392
7.270
6.251
3.610
3.283
3.269
3.256
3.241
2.362
2.346
2.331
2.254
1.406
1.380
F3C
O
N
H
N
2.0
2.0
0.8
2.0
2.1
2.3
3.8
6.0
%

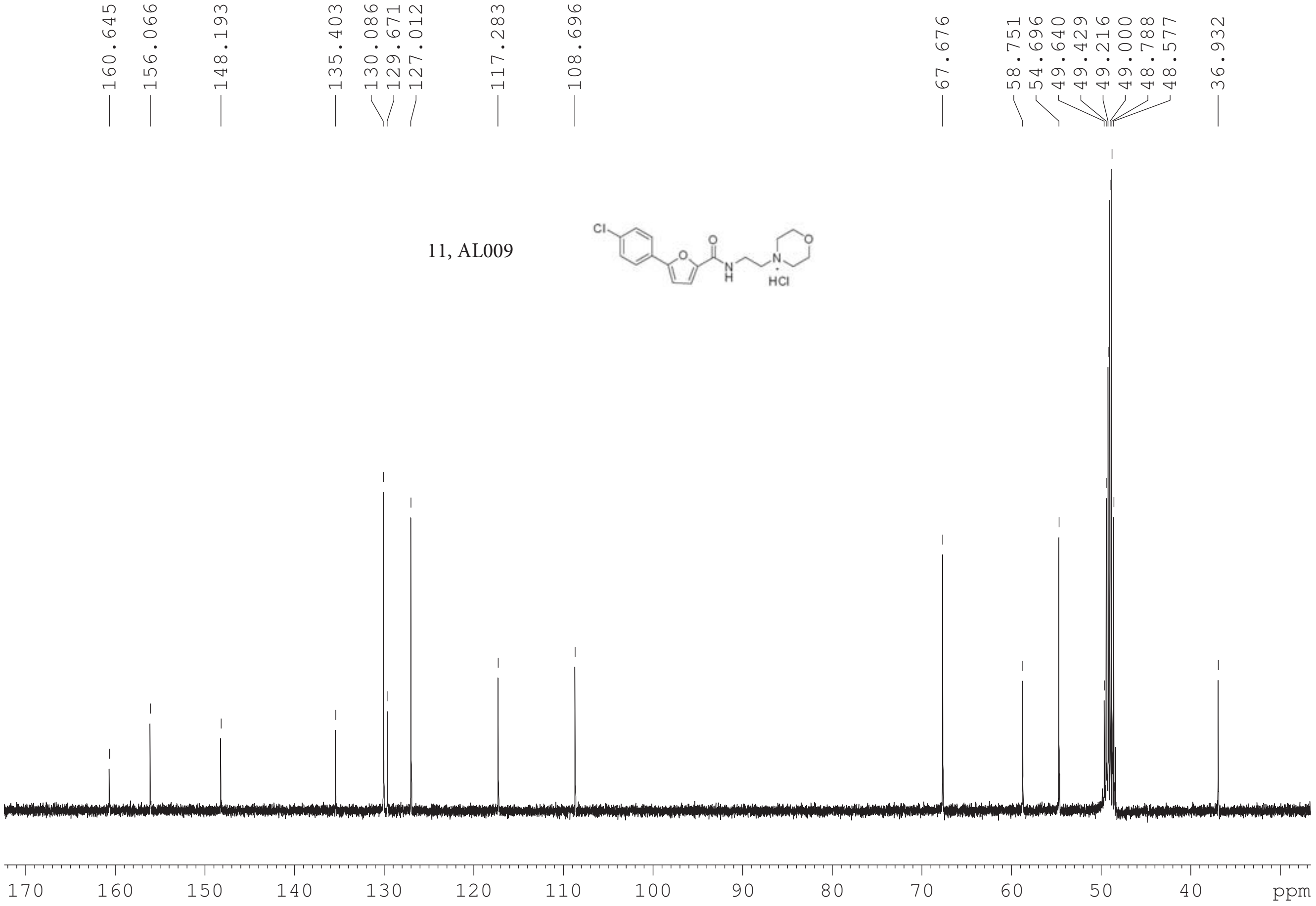
160.645
156.066
148.193
135.403
130.086
129.671
127.012
117.283
108.696
67.676
58.751
54.696
49.640
49.429
49.216
49.000
48.788
48.577
36.932
11, AL009
Cl
O
N
H
HCl
170 160 150 140 130 120 110 100 90 80 70 60 50 40 ppm

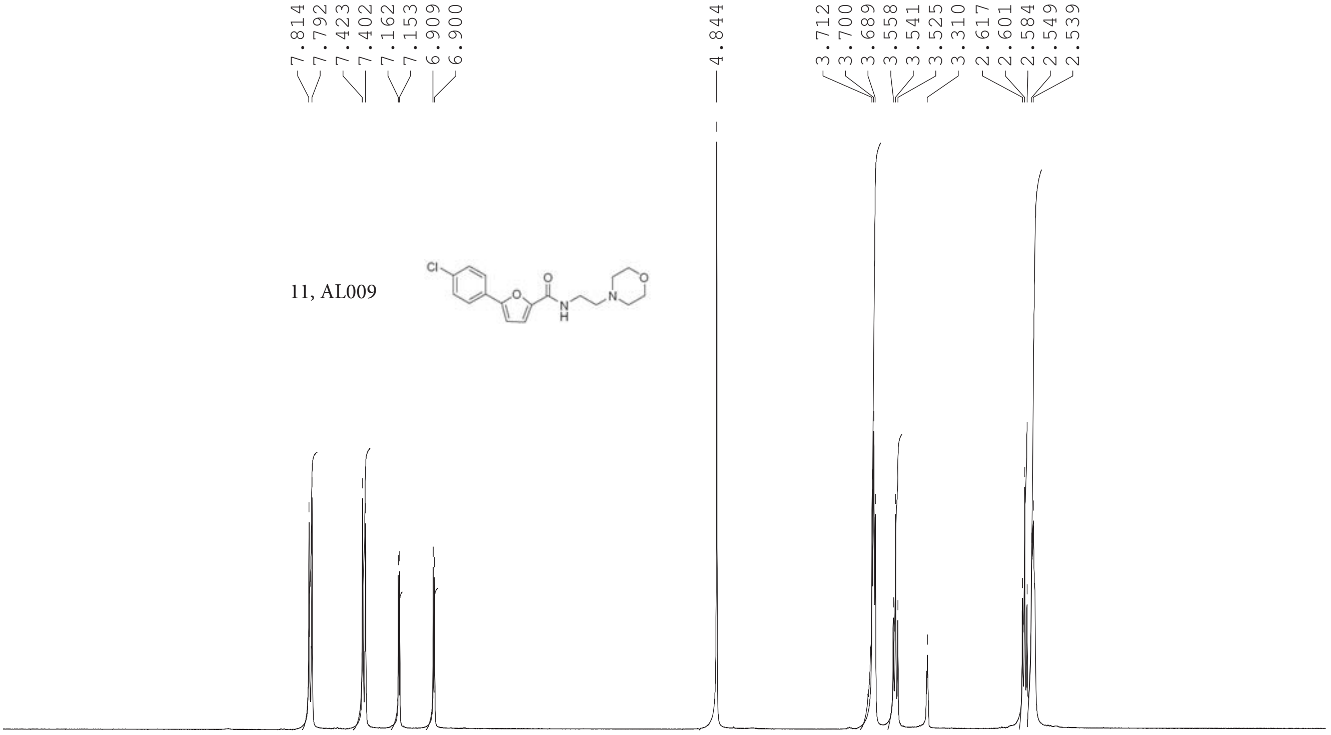
7.814
7.792
7.423
7.402
7.162
7.153
6.909
6.900
4.844
3.712
3.700
3.689
3.558
3.541
3.525
3.310
2.617
2.601
2.584
2.549
2.539
11, AL009
Cl
O
N
H
9 8 7 6 5 4 3 2 1 ppm
1.879
1.905
0.935
0.961
3.973
2.000
2.084
3.787

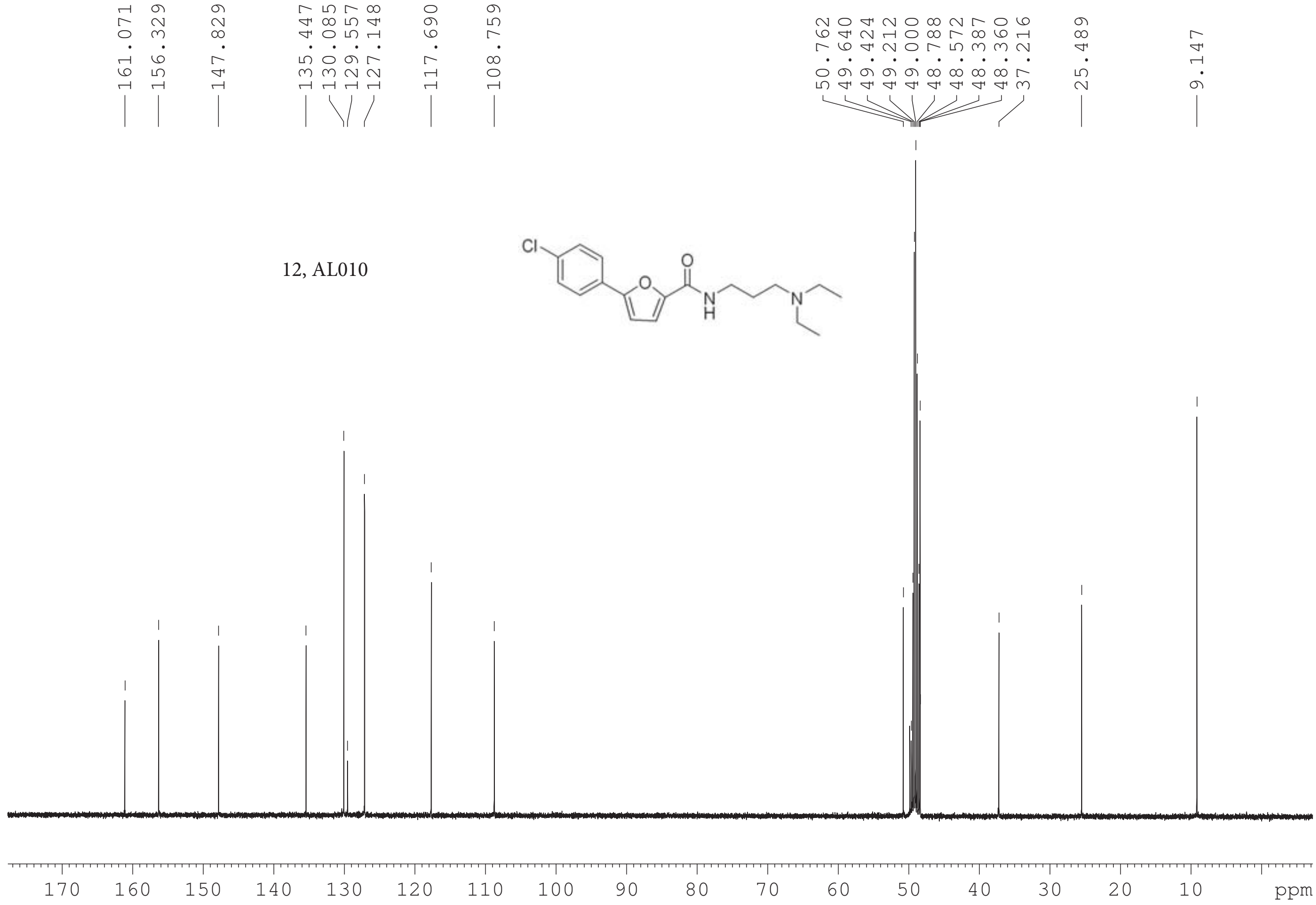
161.071
156.329
147.829
135.447
130.085
129.557
127.148
117.690
108.759
50.762
49.640
49.424
49.212
49.000
48.788
48.572
48.387
48.360
37.216
25.489
9.147
12, AL010
Cl
O
O
N
H
N
170 160 150 140 130 120 110 100 90 80 70 60 50 40 30 20 10 ppm

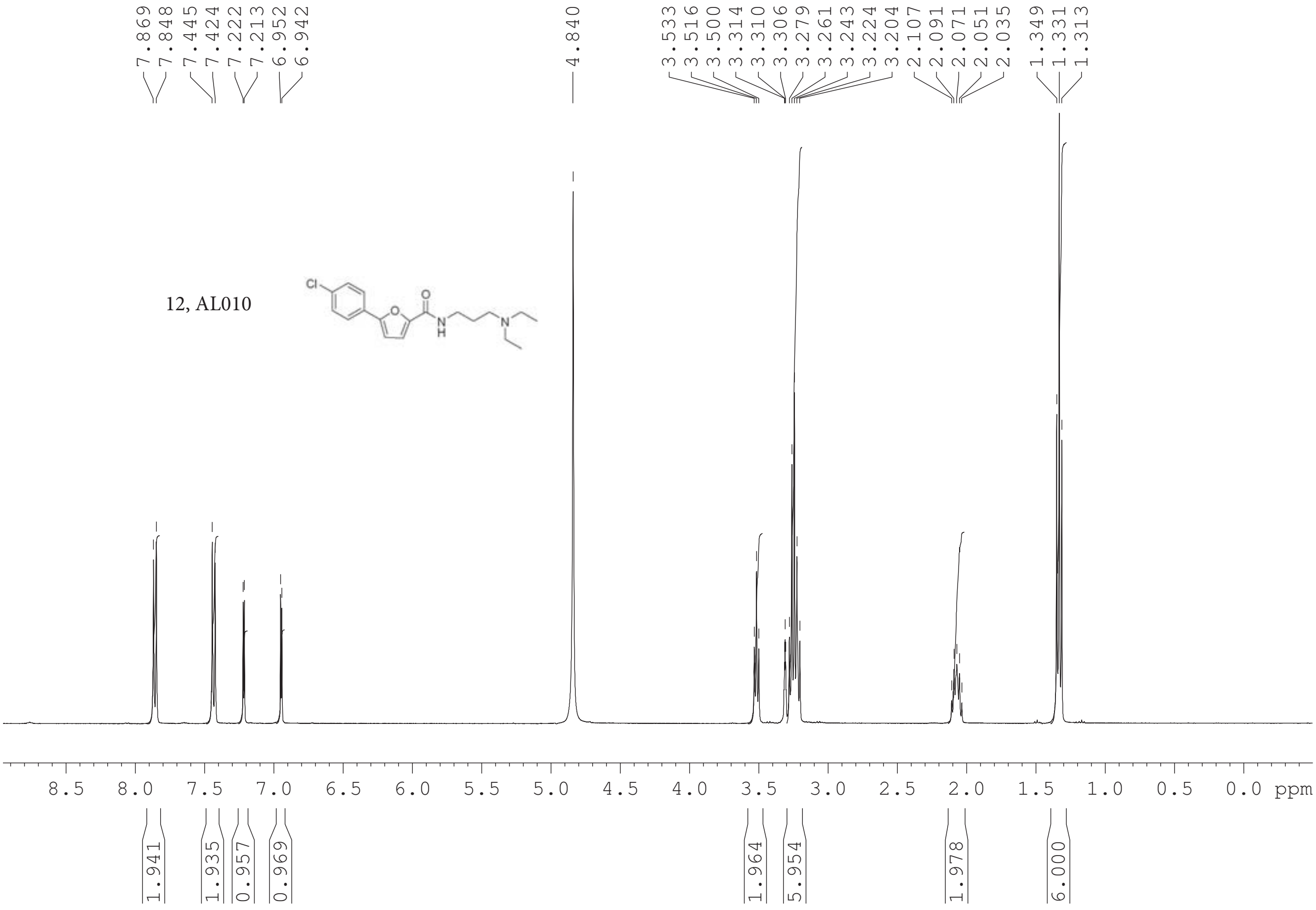
7.869
7.848
7.445
7.424
7.222
7.213
6.952
6.942
4.840
3.533
3.516
3.500
3.314
3.310
3.306
3.279
3.261
3.243
3.224
3.204
2.107
2.091
2.071
2.051
2.035
1.349
1.331
1.313
12, AL010
8.5 8.0 7.5 7.0 6.5 6.0 5.5 5.0 4.5 4.0 3.5 3.0 2.5 2.0 1.5 1.0 0.5 0.0 ppm
1.941
1.935
0.957
0.969
1.964
5.954
1.978
6.000

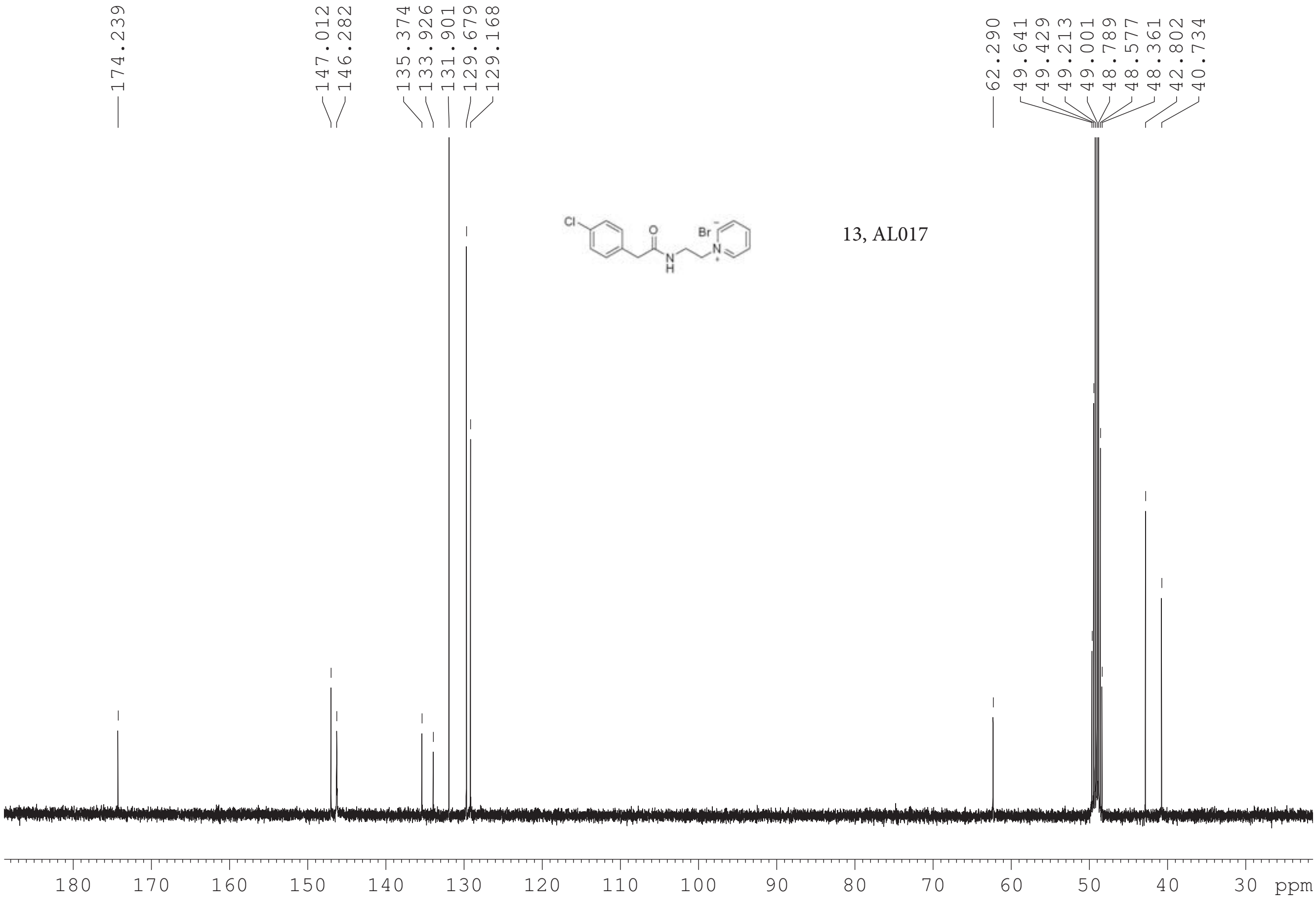

174.239
147.012
146.282
135.374
133.926
131.901
129.679
129.168
62.290
49.641
49.429
49.213
49.001
48.789
48.577
48.361
42.802
40.734
Cl
Br
N
H
13, AL017
180 170 160 150 140 130 120 110 100 90 80 70 60 50 40 30 ppm

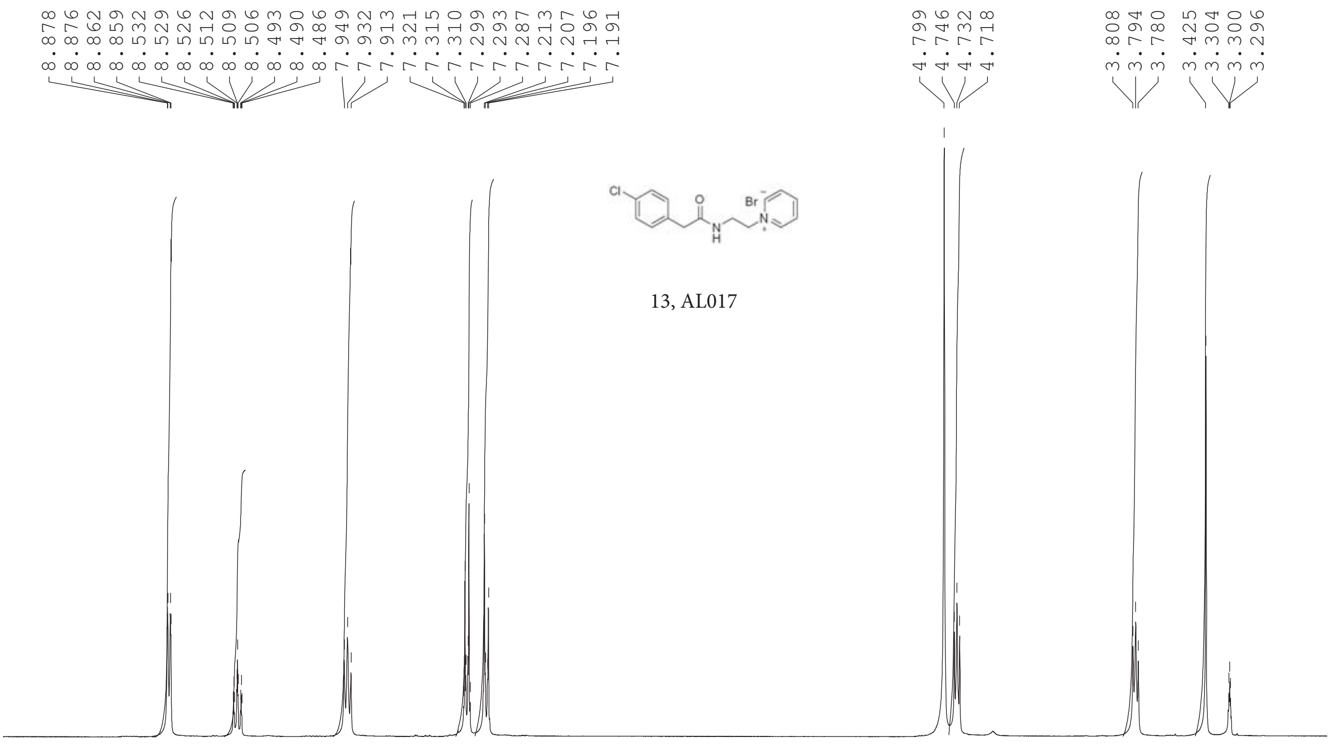

8.878
8.876
8.862
8.859
8.532
8.529
8.526
8.512
8.509
8.506
8.493
8.490
8.486
7.949
7.932
7.913
7.321
7.315
7.310
7.299
7.293
7.287
7.213
7.207
7.196
7.191
4.799
4.746
4.732
4.718
3.808
3.794
3.780
3.425
3.304
3.300
3.296
Cl
Br
N
H
13, AL017
9.5 9.0 8.5 8.0 7.5 7.0 6.5 6.0 5.5 5.0 4.5 4.0 3.5 ppm
1.911
0.944
1.898
1.902
1.975
2.085
2.000
1.989

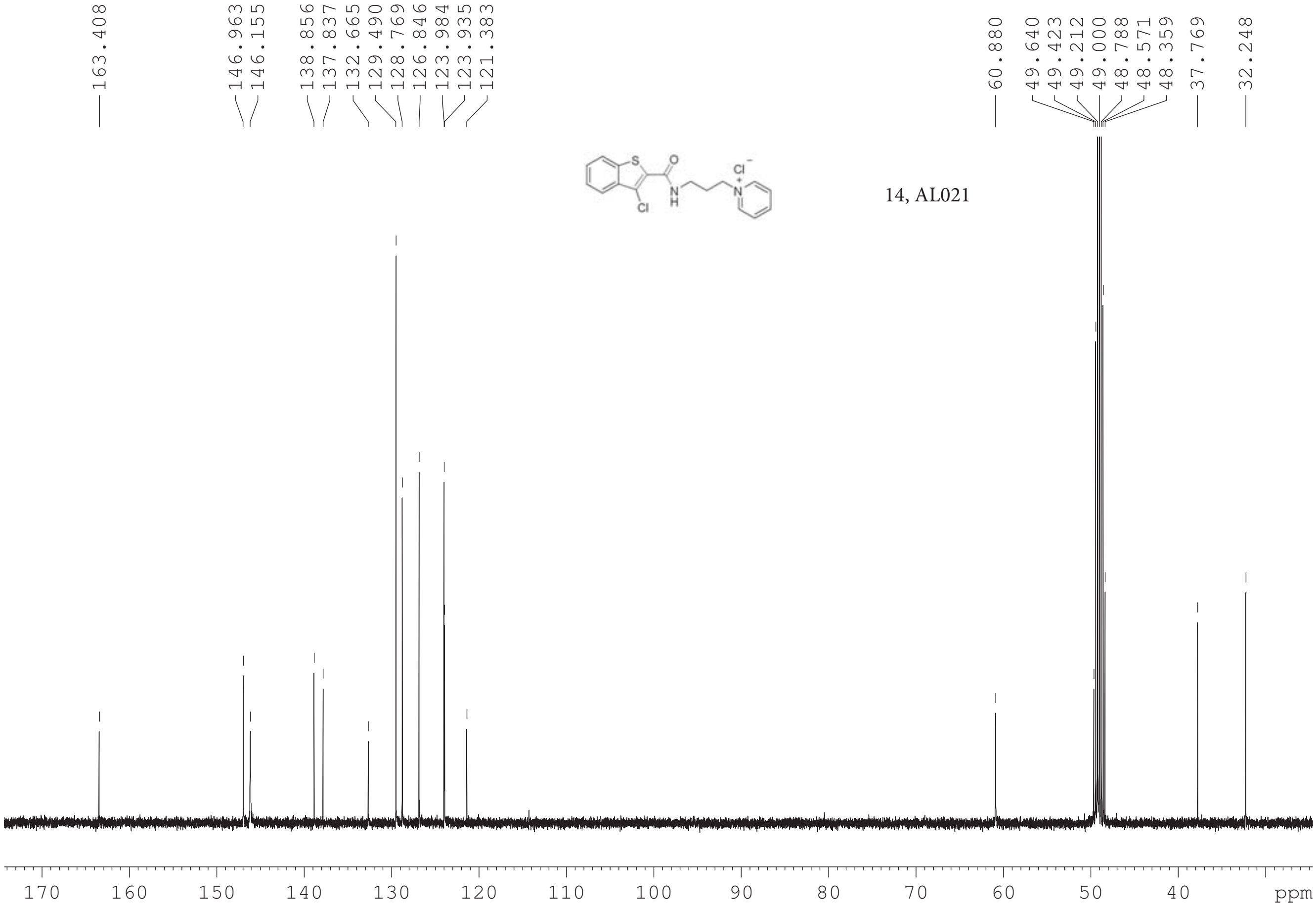
163.408
146.963
146.155
138.856
137.837
132.665
129.490
128.769
126.846
123.984
123.935
121.383
60.880
49.640
49.423
49.212
49.000
48.788
48.571
48.359
37.769
32.248
S
O
N
H
Cl
N
+
Cl
−
14, AL021
170 160 150 140 130 120 110 100 90 80 70 60 50 40 ppm

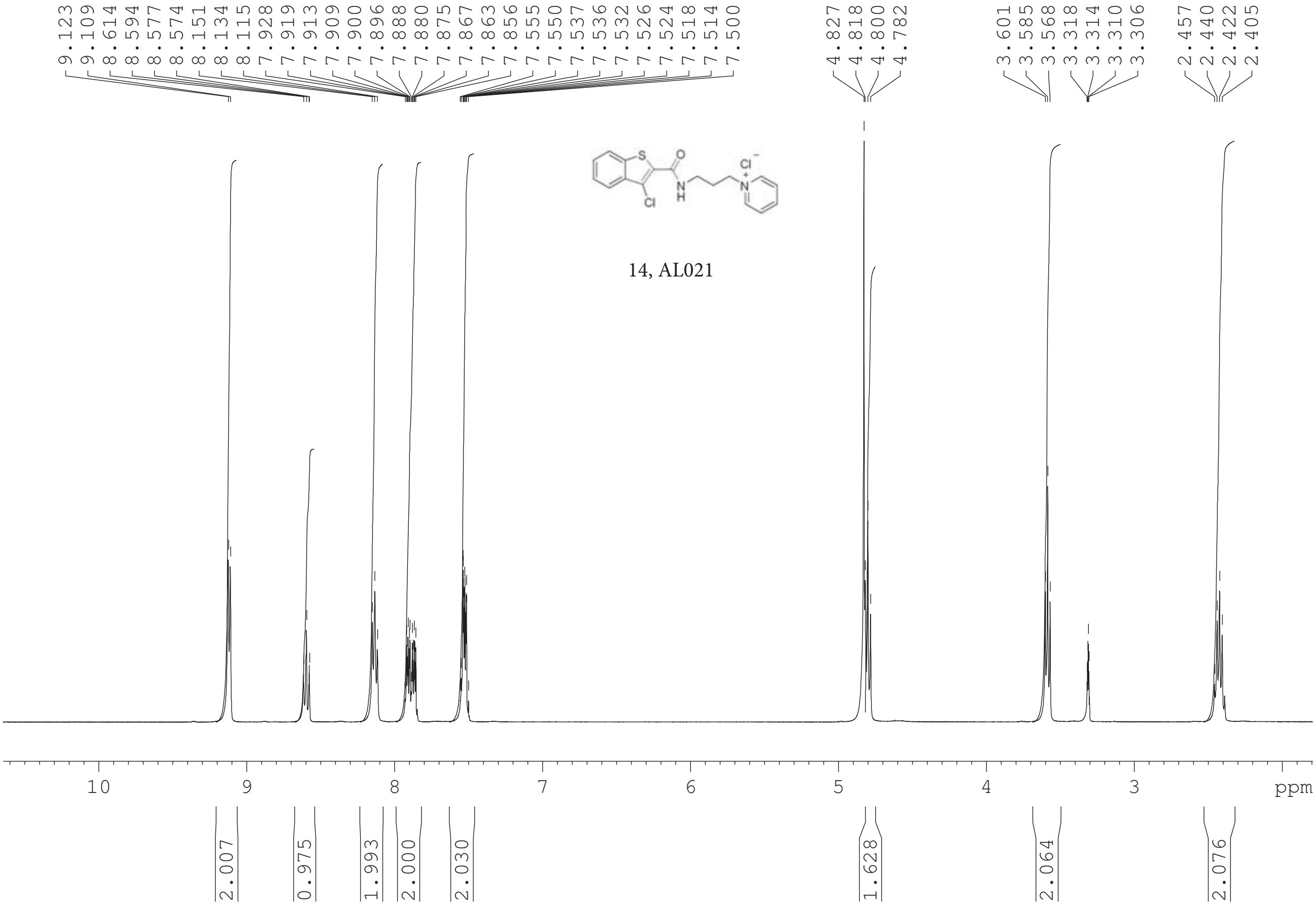
9.123
9.109
8.614
8.594
8.577
8.574
8.151
8.134
8.115
7.928
7.919
7.913
7.909
7.900
7.896
7.888
7.880
7.875
7.867
7.863
7.856
7.555
7.550
7.537
7.536
7.532
7.526
7.524
7.518
7.514
7.500
4.827
4.818
4.800
4.782
3.601
3.585
3.568
3.318
3.314
3.310
3.306
2.457
2.440
2.422
2.405
S
O
N
H
Cl
N
+
Cl
−
14, AL021
10 9 8 7 6 5 4 3 ppm
2.007
0.975
1.993
2.000
2.030
1.628
2.064
2.076

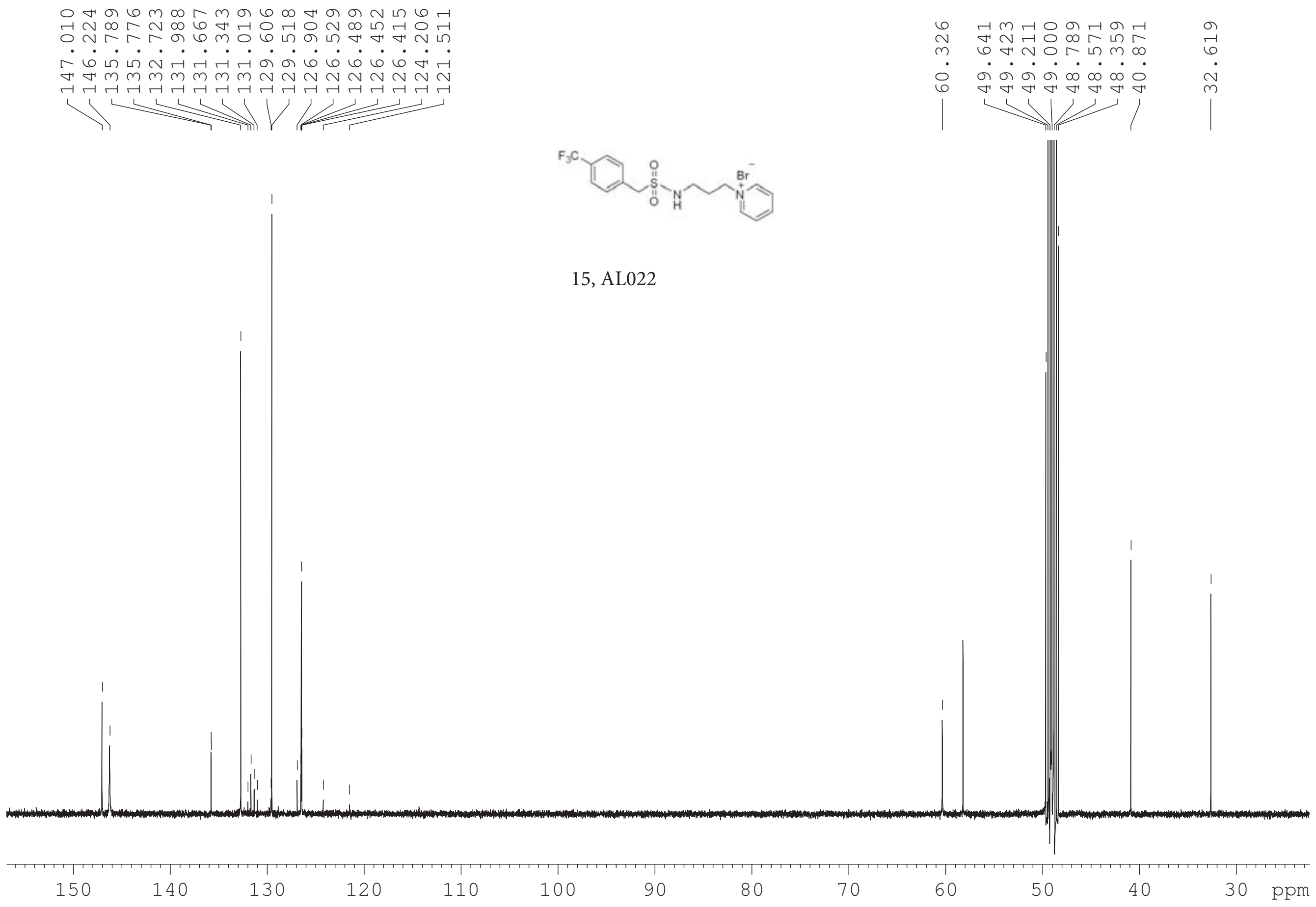
147.010
146.224
135.789
135.776
132.723
131.988
131.667
131.343
131.019
129.606
129.518
126.904
126.529
126.489
126.452
126.415
124.206
121.511
60.326
49.641
49.423
49.211
49.000
48.789
48.571
48.359
40.871
32.619
F_3C
S
O
O
N
H
Br
N
15, AL022
150
140
130
120
110
100
90
80
70
60
50
40
30
ppm

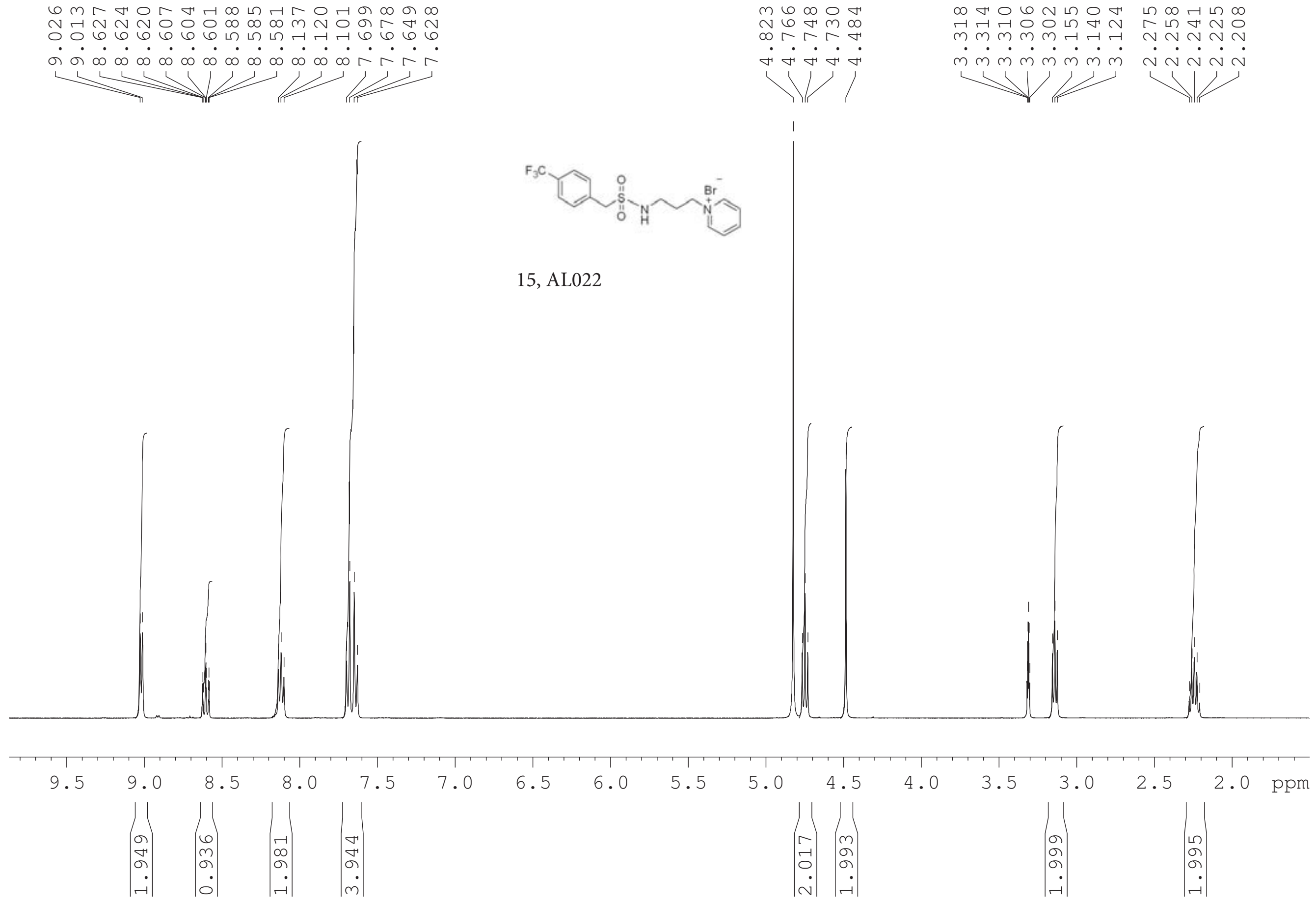
9.026
9.013
8.627
8.624
8.620
8.607
8.604
8.601
8.588
8.585
8.581
8.137
8.120
8.101
7.699
7.678
7.649
7.628
4.823
4.766
4.748
4.730
4.484
3.318
3.314
3.310
3.306
3.302
3.155
3.140
3.124
2.275
2.258
2.241
2.225
2.208
F_3C
S
O
O
N
H
Br
N
15, AL022
9.5
9.0
8.5
8.0
7.5
7.0
6.5
6.0
5.5
5.0
4.5
4.0
3.5
3.0
2.5
2.0
ppm
1.949
0.936
1.981
3.944
2.017
1.993
1.999
1.995

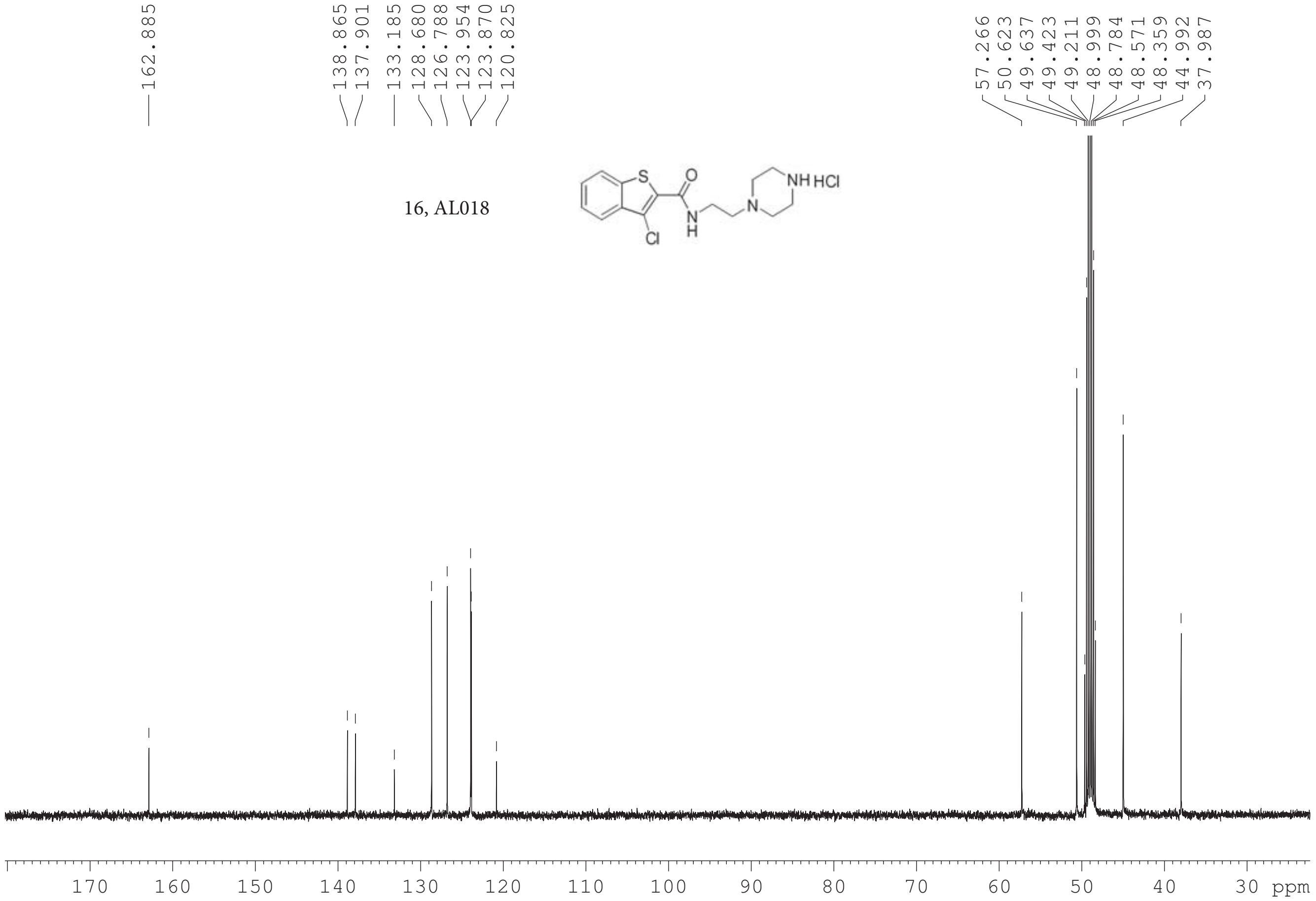
162.885
138.865
137.901
133.185
128.680
126.788
123.954
123.870
120.825
57.266
50.623
49.637
49.423
49.211
48.999
48.784
48.571
48.359
44.992
37.987
16, AL018
S
O
NH HCl
N
H
N
Cl
170 160 150 140 130 120 110 100 90 80 70 60 50 40 30 ppm

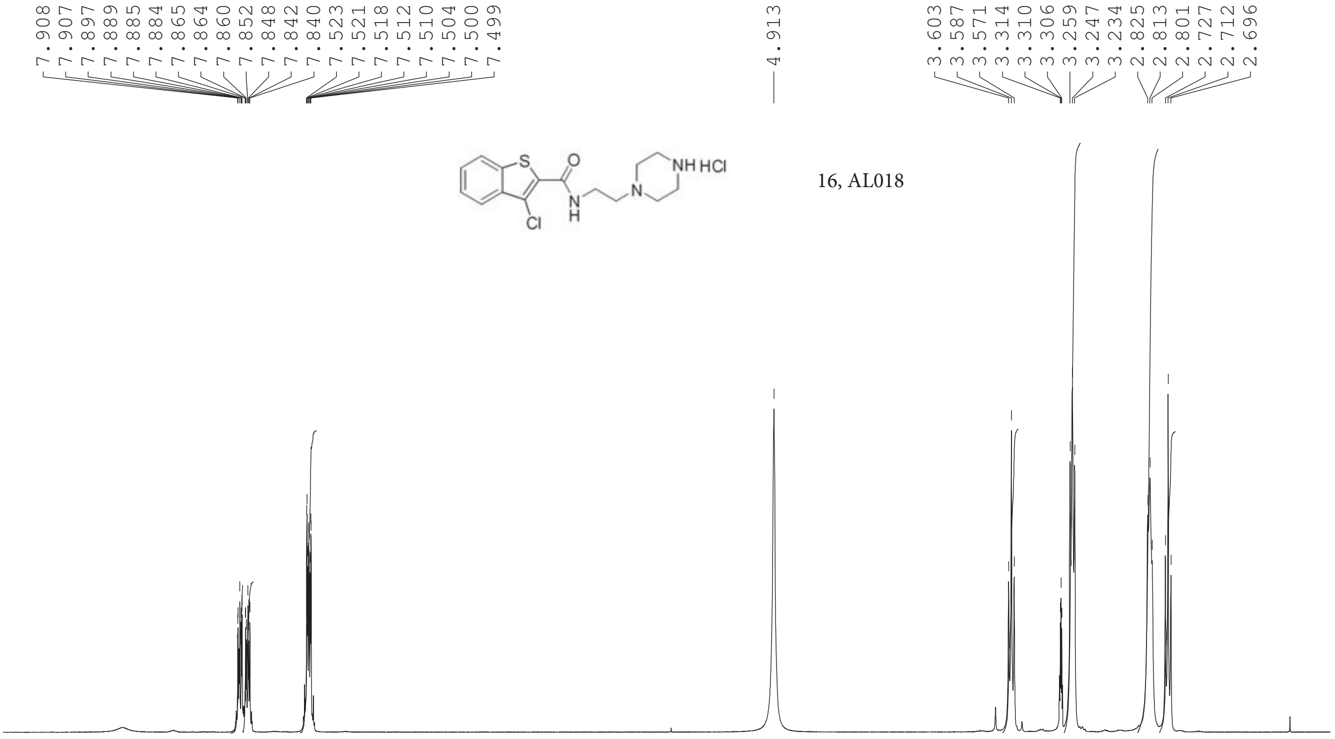
7.908
7.907
7.897
7.889
7.885
7.884
7.865
7.864
7.860
7.852
7.848
7.842
7.840
7.523
7.521
7.518
7.512
7.510
7.504
7.500
7.499
4.913
3.603
3.587
3.571
3.314
3.310
3.306
3.259
3.247
3.234
2.825
2.813
2.801
2.727
2.712
2.696
S
O
NH HCl
N
H
N
Cl
16, AL018
9.0 8.5 8.0 7.5 7.0 6.5 6.0 5.5 5.0 4.5 4.0 3.5 3.0 2.5 ppm
0.982
1.002
2.005
2.016
3.914
3.874
2.000

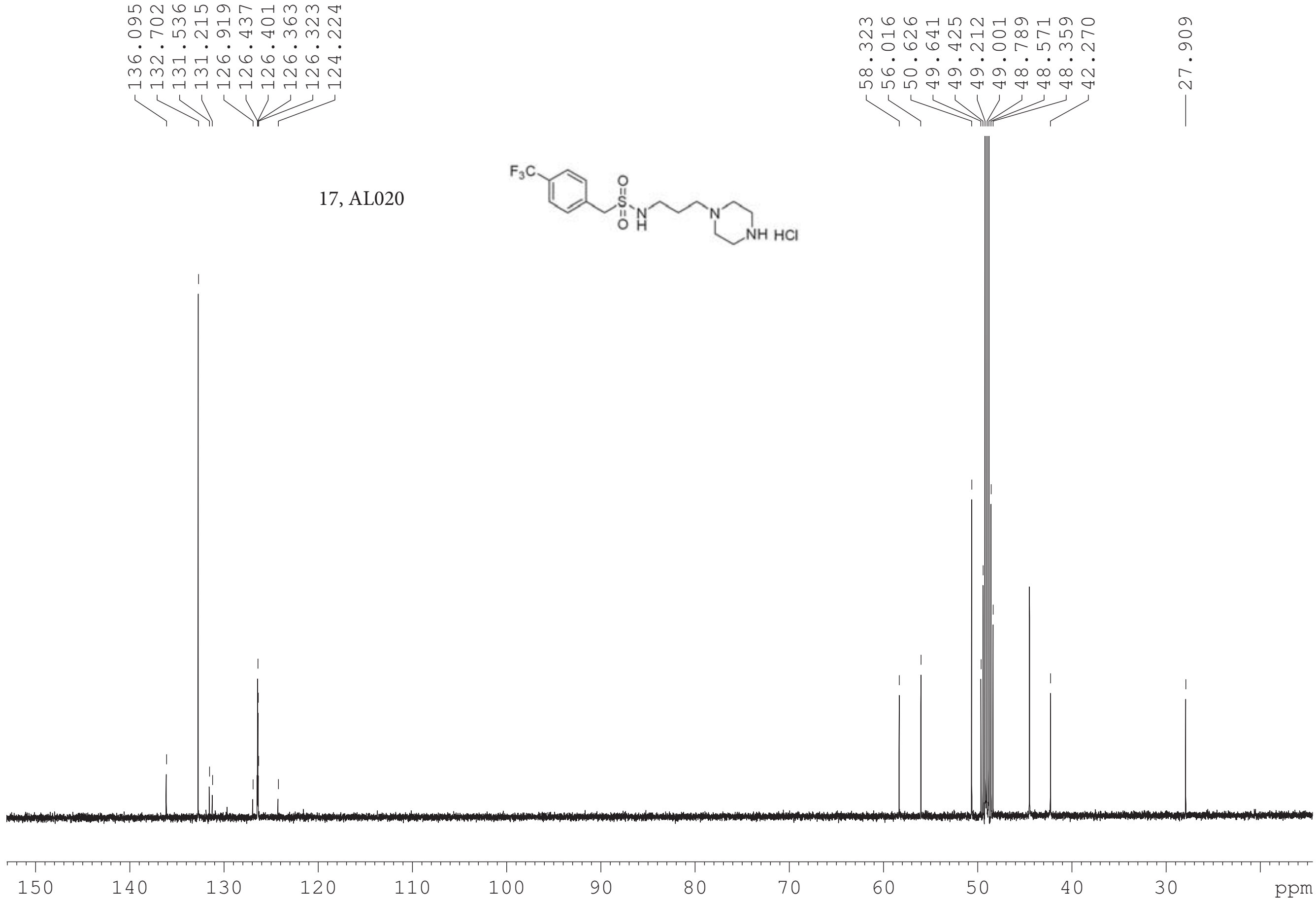
136.095
132.702
131.536
131.215
126.919
126.437
126.401
126.363
126.323
124.224
58.323
56.016
50.626
49.641
49.425
49.212
49.001
48.789
48.571
48.359
42.270
27.909
17, AL020
F3C
S
O
O
N
H
N
NH HCl
150
140
130
120
110
100
90
80
70
60
50
40
30
ppm

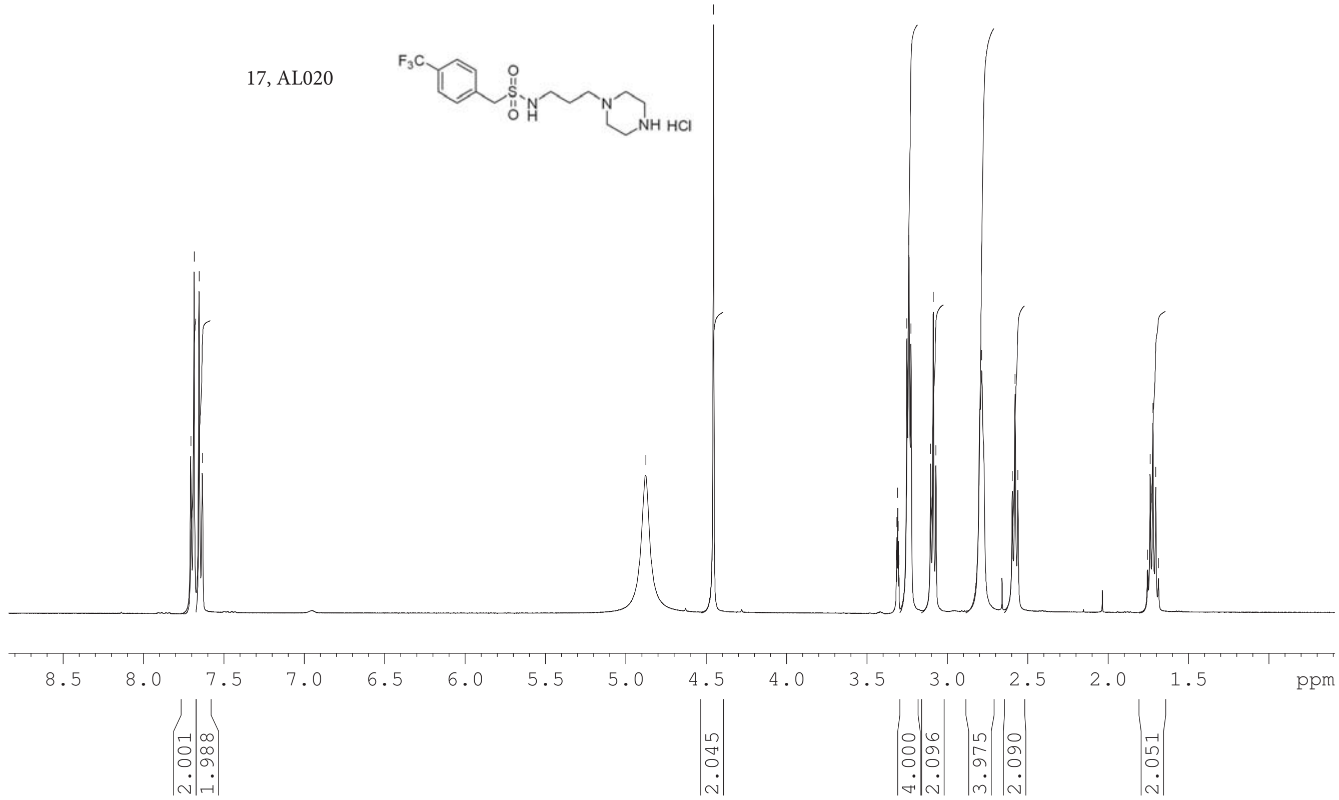
7.705
7.684
7.654
7.633
4.875
4.455
3.318
3.314
3.310
3.306
3.302
3.252
3.240
3.227
3.105
3.088
3.071
2.787
2.597
2.579
2.562
1.756
1.739
1.722
1.704
1.687
17, AL020
F3C
S
O
O
N
H
N
NH HCl
8.5
8.0
7.5
7.0
6.5
6.0
5.5
5.0
4.5
4.0
3.5
3.0
2.5
2.0
1.5
ppm
2.001
1.988
2.045
4.000
2.096
3.975
2.090
2.051

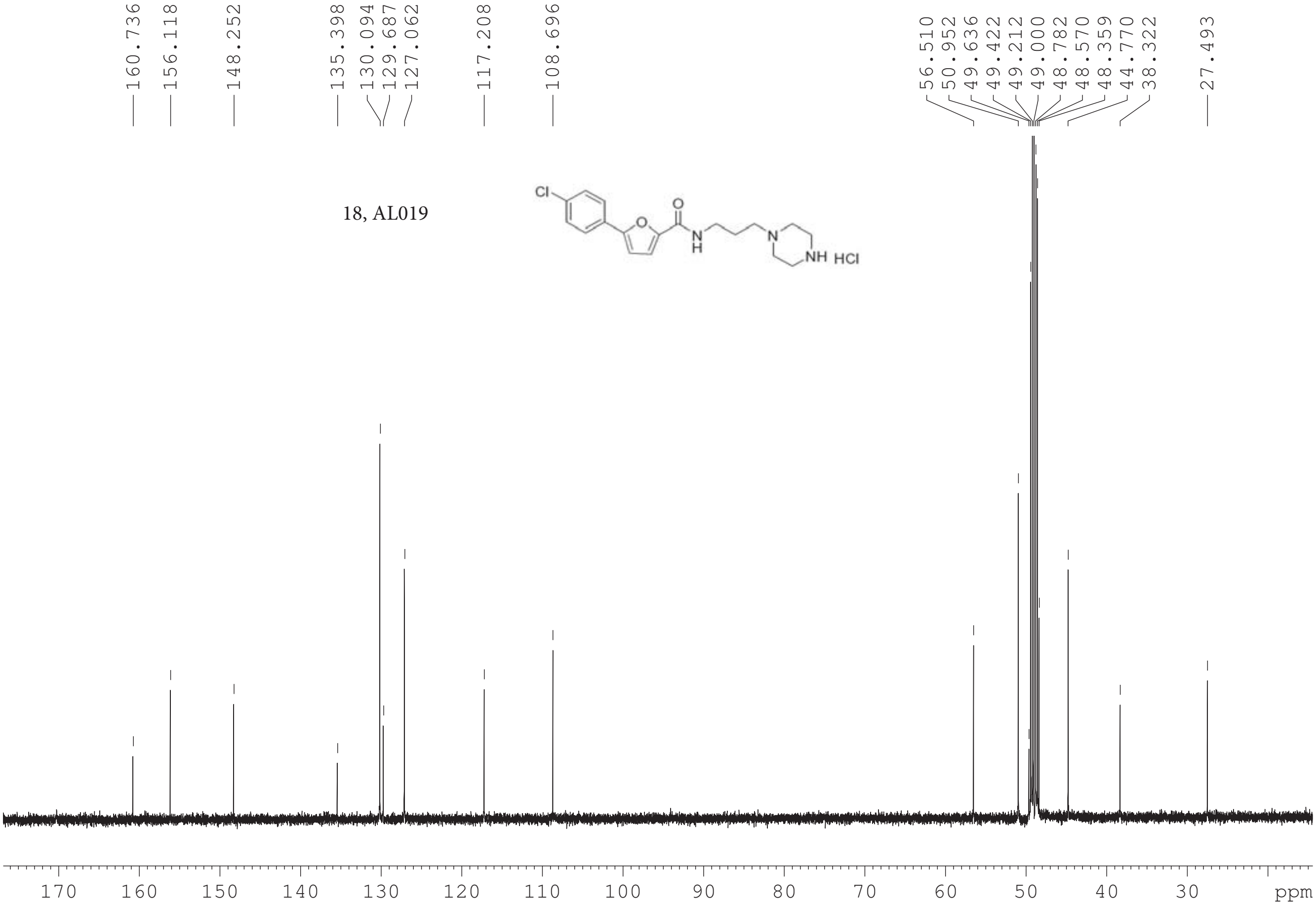
160.736
156.118
148.252
135.398
130.094
129.687
127.062
117.208
108.696
56.510
50.952
49.636
49.422
49.212
49.000
48.782
48.570
48.359
44.770
38.322
27.493
18, AL019
Cl
O
N
H
N
NH HCl
170 160 150 140 130 120 110 100 90 80 70 60 50 40 30 ppm

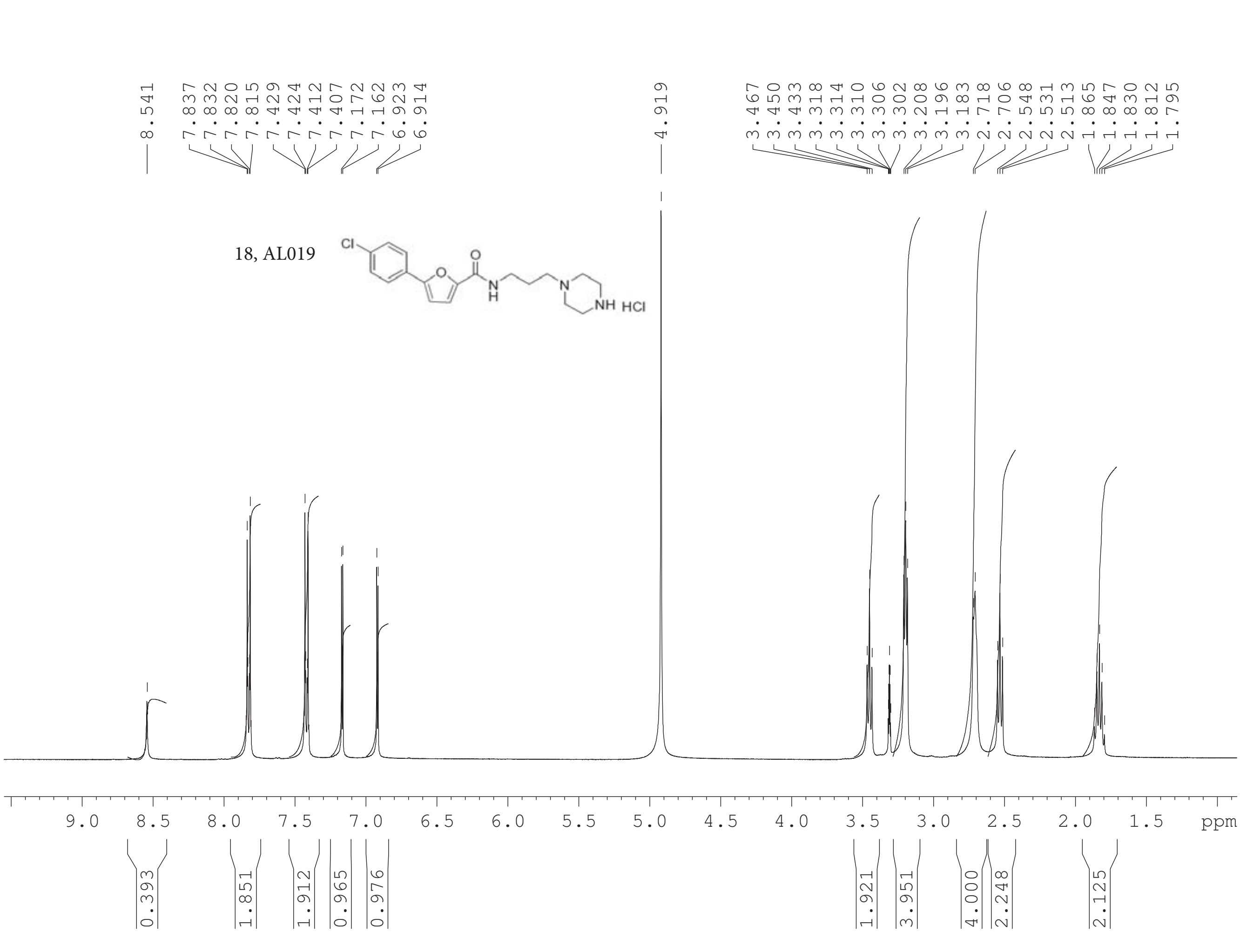
8.541
7.837
7.832
7.820
7.815
7.429
7.424
7.412
7.407
7.172
7.162
6.923
6.914
4.919
3.467
3.450
3.433
3.318
3.314
3.310
3.306
3.302
3.208
3.196
3.183
2.718
2.706
2.548
2.531
2.513
1.865
1.847
1.830
1.812
1.795
18, AL019
Cl
O
N
H
N
NH HCl
9.0 8.5 8.0 7.5 7.0 6.5 6.0 5.5 5.0 4.5 4.0 3.5 3.0 2.5 2.0 1.5 ppm
0.393
1.851
1.912
0.965
0.976
1.921
3.951
4.000
2.248
2.125

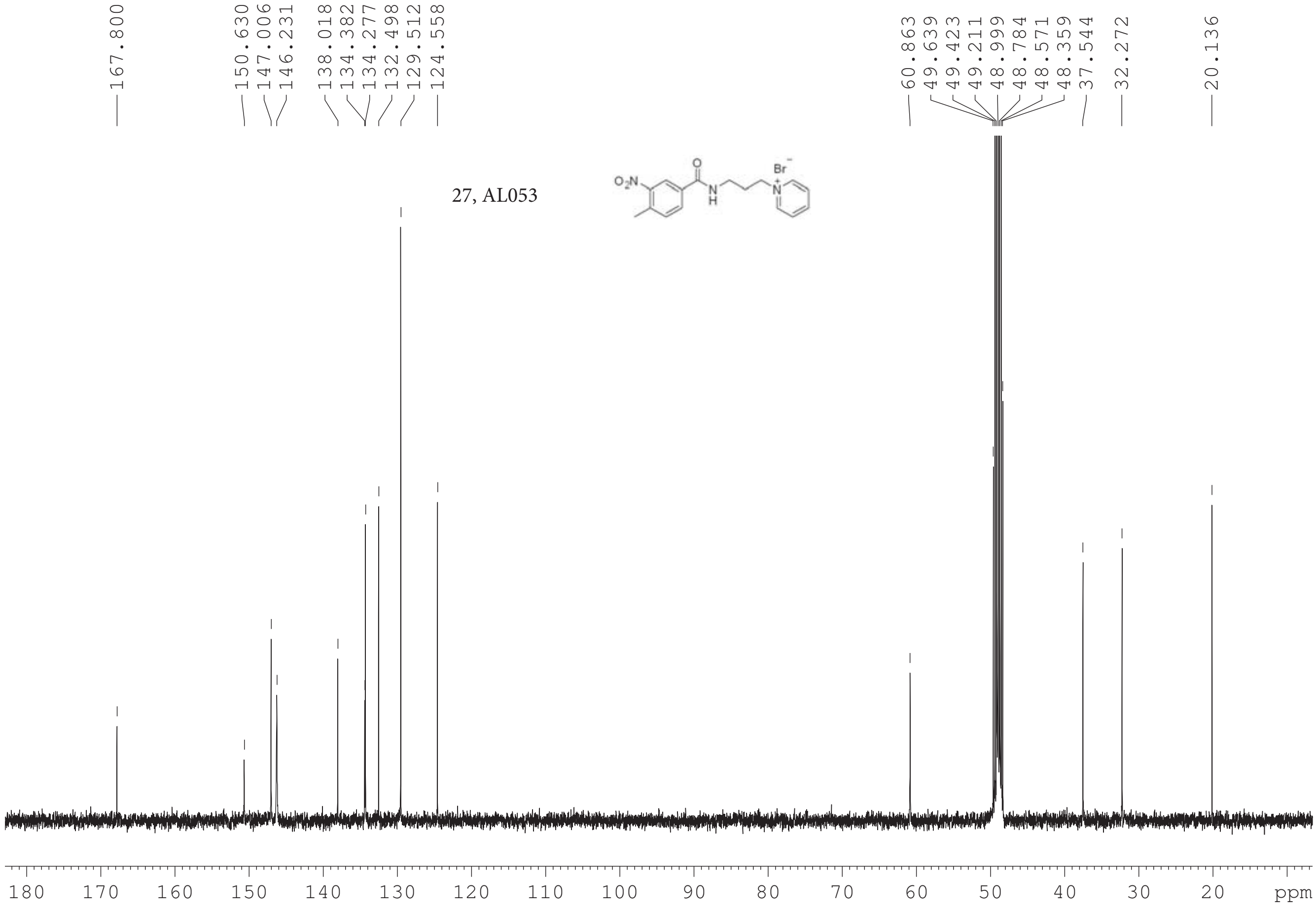
167.800
150.630
147.006
146.231
138.018
134.382
134.277
132.498
129.512
124.558
60.863
49.639
49.423
49.211
48.999
48.784
48.571
48.359
37.544
32.272
20.136
27, AL053
180 170 160 150 140 130 120 110 100 90 80 70 60 50 40 30 20 ppm

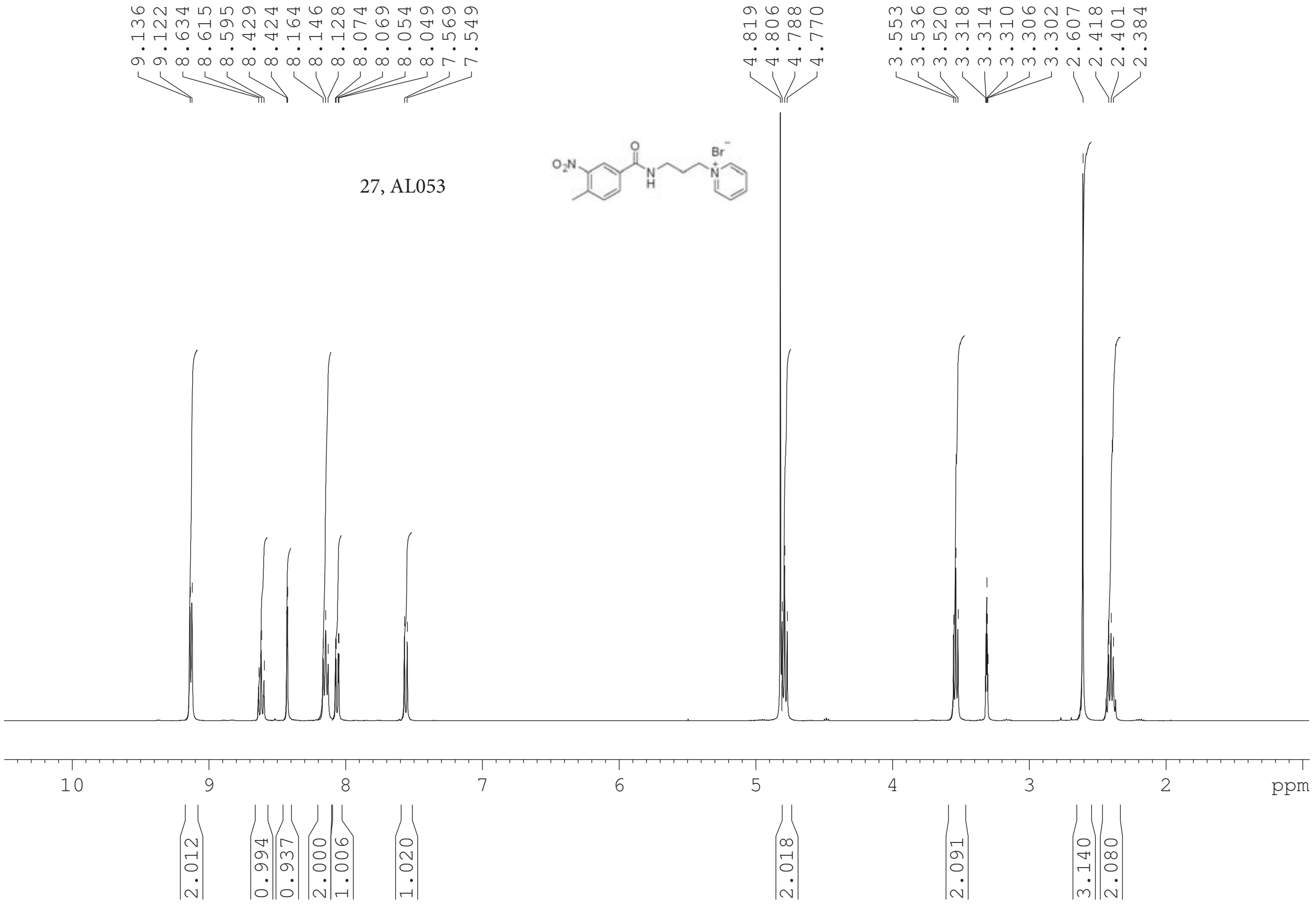
9.136
9.122
8.634
8.615
8.595
8.429
8.424
8.164
8.146
8.128
8.074
8.069
8.054
8.049
7.569
7.549
4.819
4.806
4.788
4.770
3.553
3.536
3.520
3.318
3.314
3.310
3.306
3.302
2.607
2.418
2.401
2.384
27, AL053
10 9 8 7 6 5 4 3 2 ppm
2.012
0.994
0.937
2.000
1.006
1.020
2.018
2.091
3.140
2.080

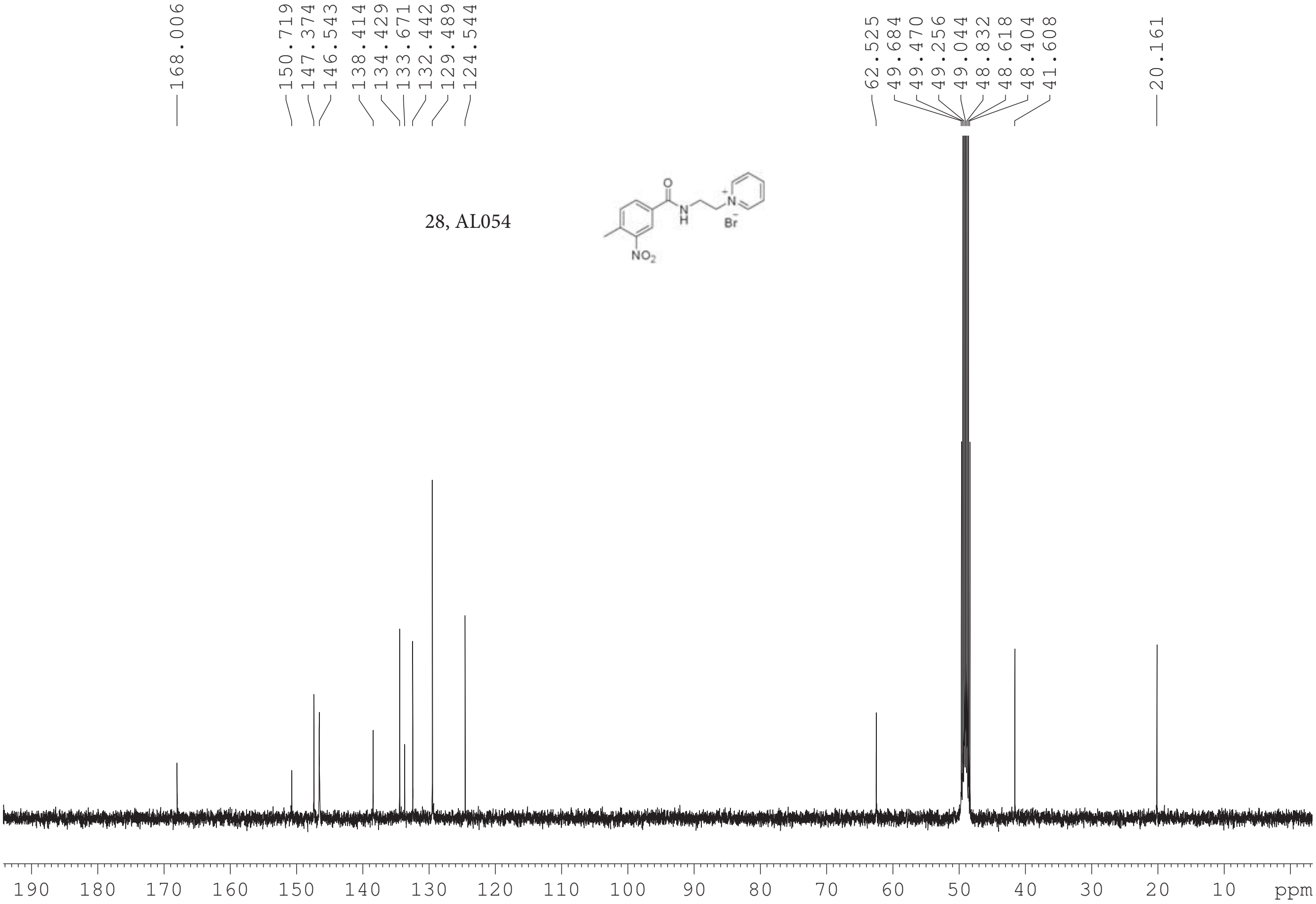
168.006
150.719
147.374
146.543
138.414
134.429
133.671
132.442
129.489
124.544
62.525
49.684
49.470
49.256
49.044
48.832
48.618
48.404
41.608
20.161
28, AL054
NO2
N
H
O
Br
190 180 170 160 150 140 130 120 110 100 90 80 70 60 50 40 30 20 10 ppm

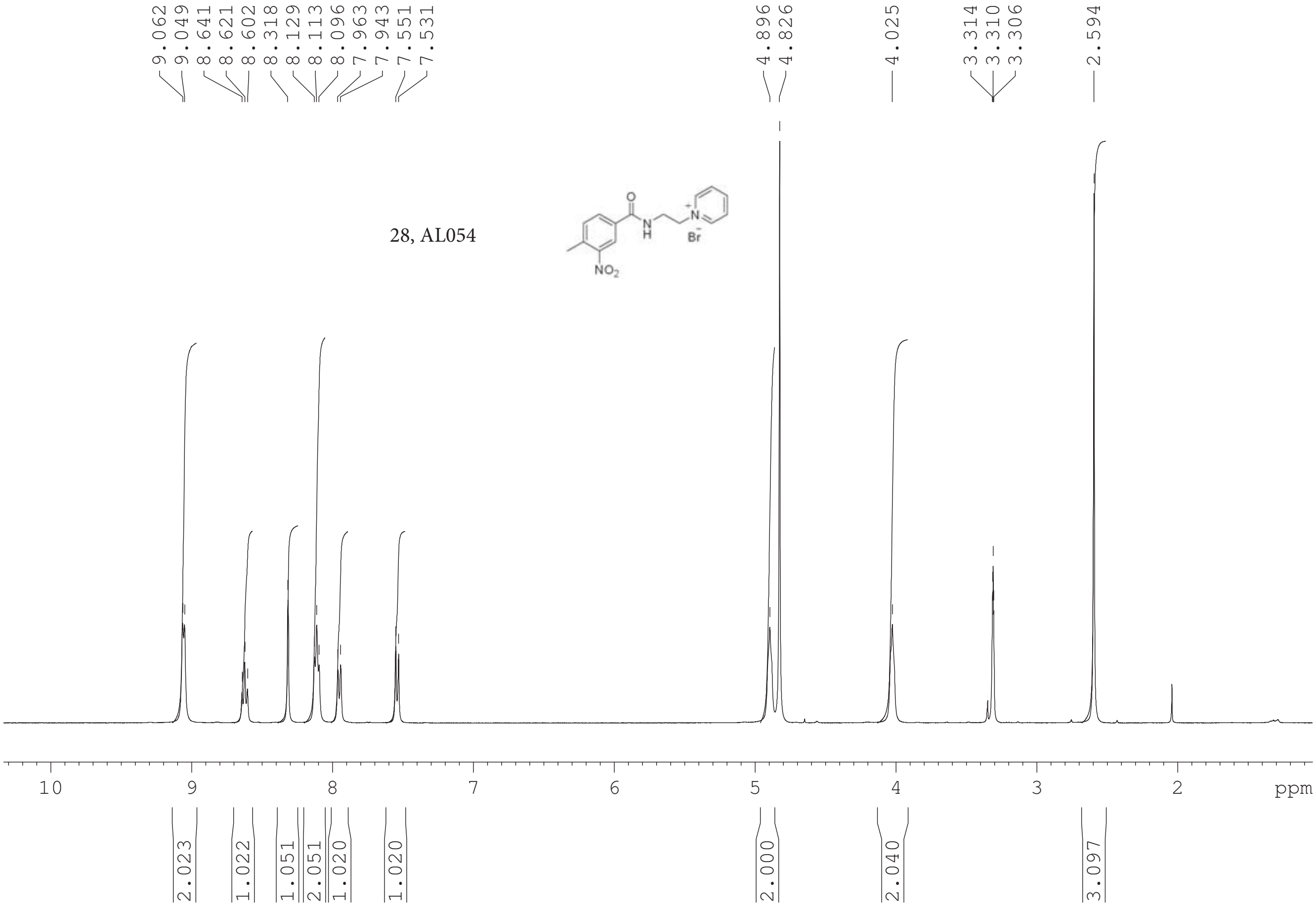
9.062
9.049
8.641
8.621
8.602
8.318
8.129
8.113
8.096
7.963
7.943
7.551
7.531
4.896
4.826
4.025
3.314
3.310
3.306
2.594
28, AL054
NO2
N
H
O
Br
10 9 8 7 6 5 4 3 2 ppm
2.023
1.022
1.051
2.051
1.020
1.020
2.000
2.040
3.097

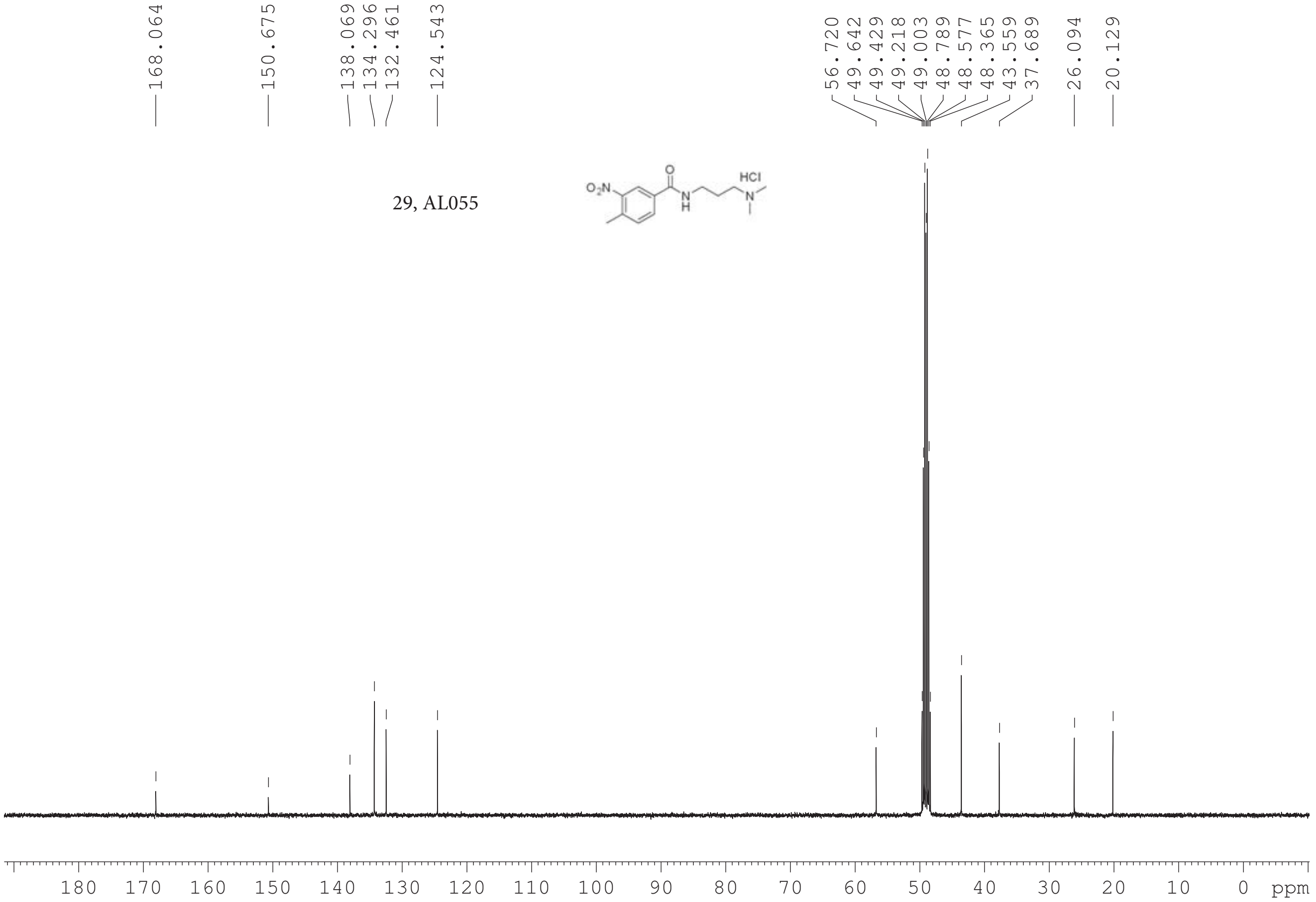
168.064
150.675
138.069
134.296
132.461
124.543
56.720
49.642
49.429
49.218
49.003
48.789
48.577
48.365
43.559
37.689
26.094
20.129
29, AL055
HCl
180 170 160 150 140 130 120 110 100 90 80 70 60 50 40 30 20 10 0 ppm

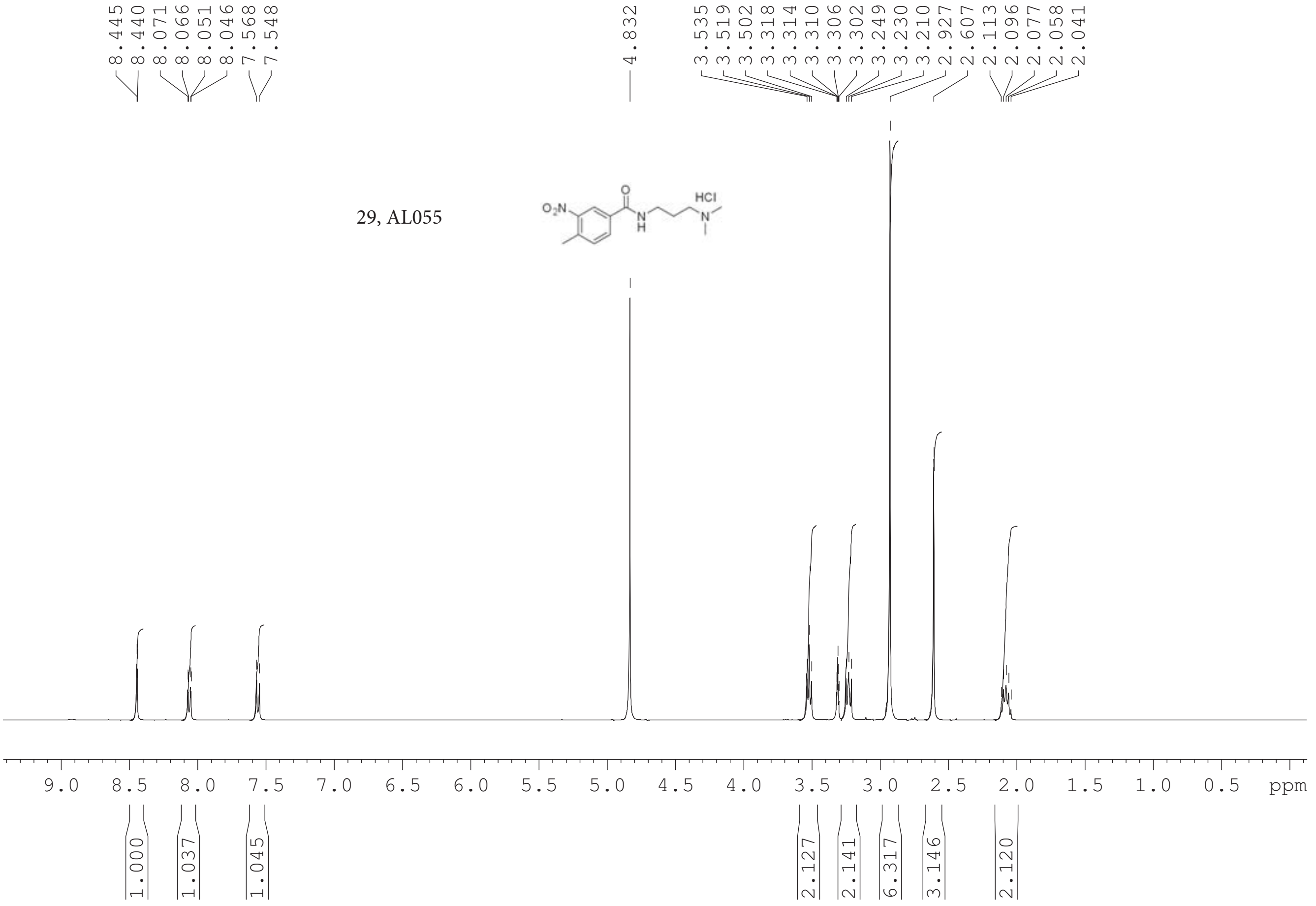
8.445
8.440
8.071
8.066
8.051
8.046
7.568
7.548
4.832
3.535
3.519
3.502
3.318
3.314
3.310
3.306
3.302
3.249
3.230
3.210
2.927
2.607
2.113
2.096
2.077
2.058
2.041
29, AL055
HCl
9.0 8.5 8.0 7.5 7.0 6.5 6.0 5.5 5.0 4.5 4.0 3.5 3.0 2.5 2.0 1.5 1.0 0.5 ppm
1.000
1.037
1.045
2.127
2.141
6.317
3.146
2.120

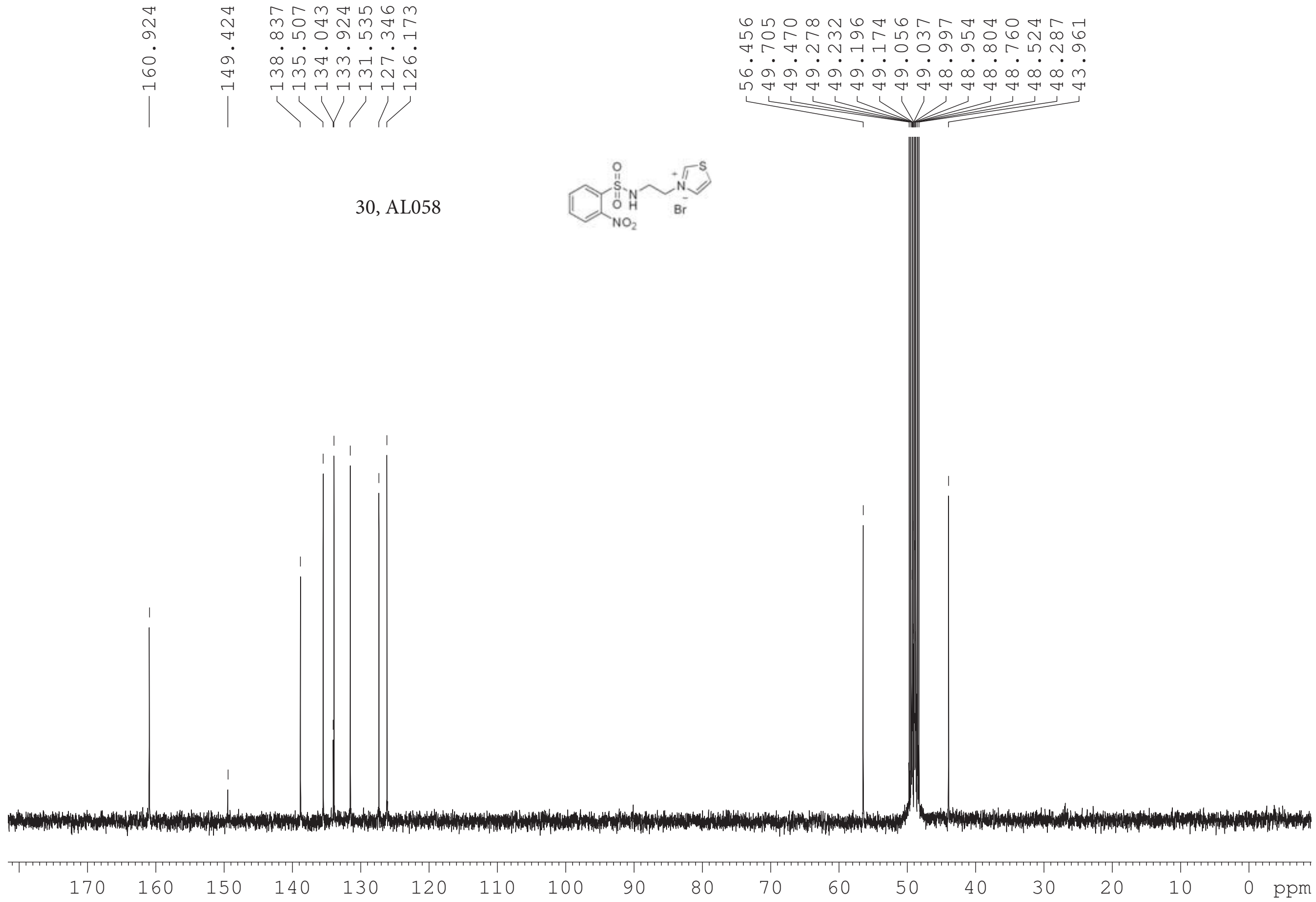
160.924
149.424
138.837
135.507
134.043
133.924
131.535
127.346
126.173
56.456
49.705
49.470
49.278
49.232
49.196
49.174
49.056
49.037
48.997
48.954
48.804
48.760
48.524
48.287
43.961
30, AL058
170 160 150 140 130 120 110 100 90 80 70 60 50 40 30 20 10 0 ppm

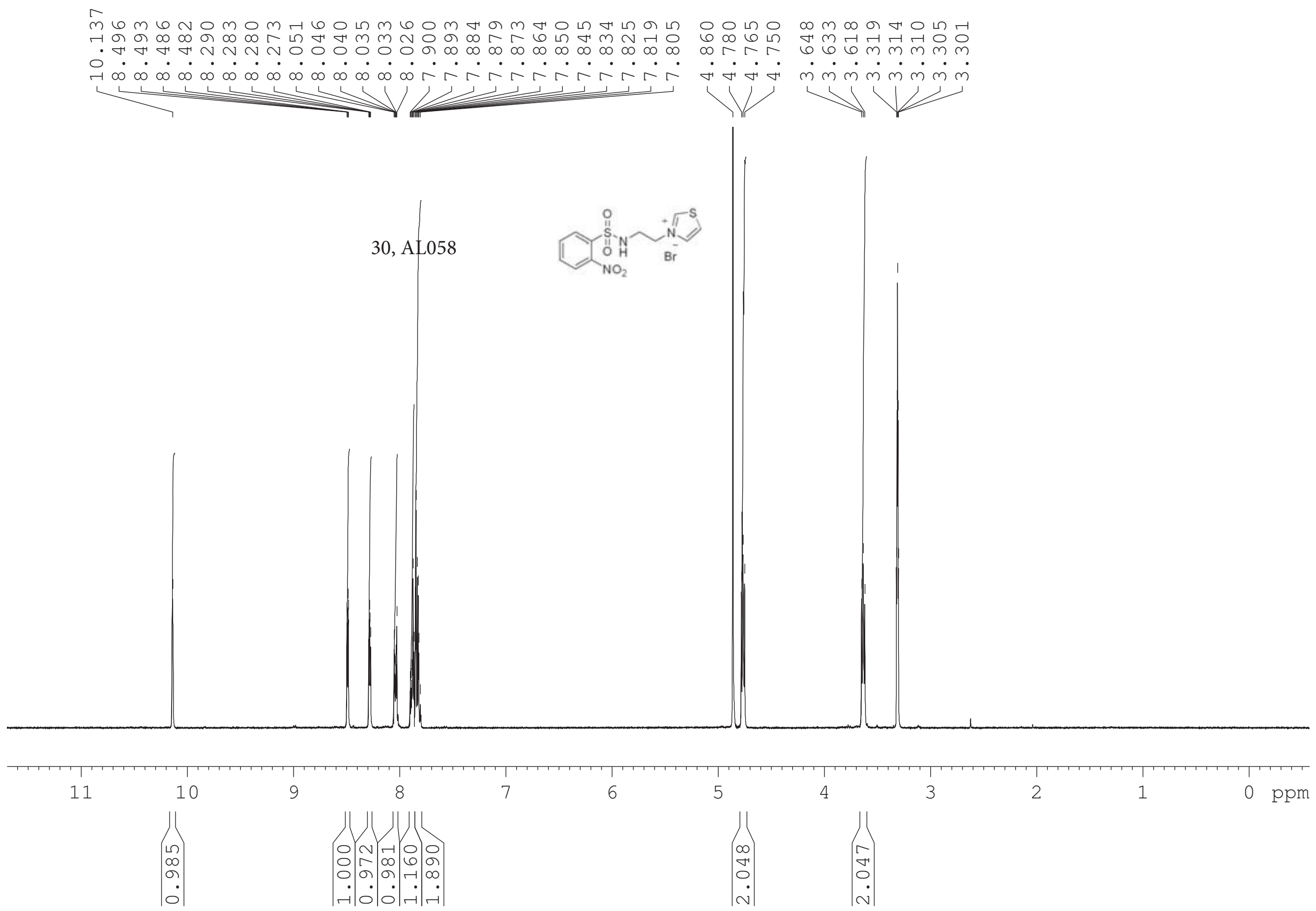
10.137
8.496
8.493
8.486
8.482
8.290
8.283
8.280
8.273
8.051
8.046
8.040
8.035
8.033
8.026
7.900
7.893
7.884
7.879
7.873
7.864
7.850
7.845
7.834
7.825
7.819
7.805
4.860
4.780
4.765
4.750
3.648
3.633
3.618
3.319
3.314
3.310
3.305
3.301
30, AL058
11 10 9 8 7 6 5 4 3 2 1 0 ppm
0.985
1.000
0.972
0.981
1.160
1.890
2.048
2.047

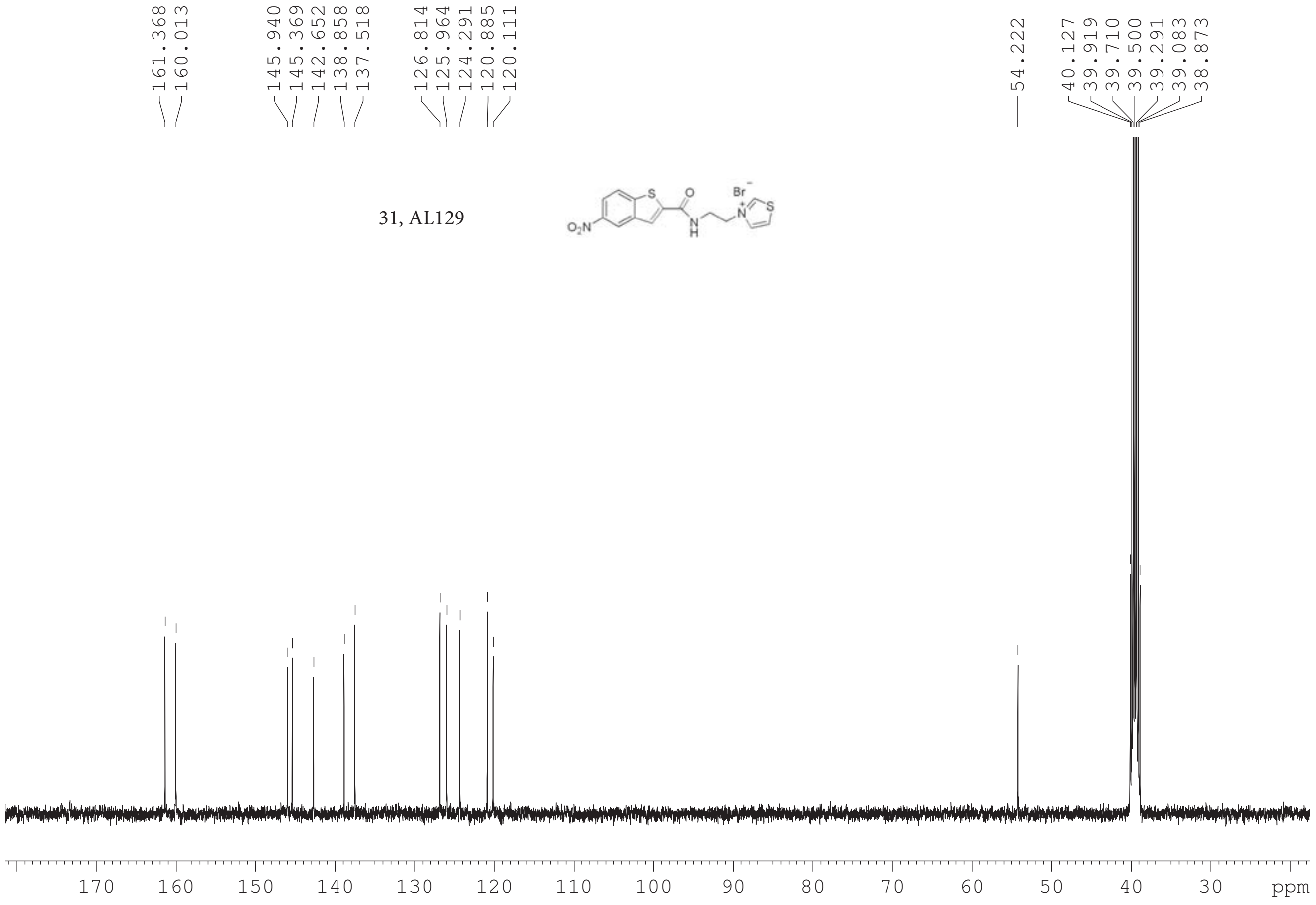
161.368
160.013
145.940
145.369
142.652
138.858
137.518
126.814
125.964
124.291
120.885
120.111
54.222
40.127
39.919
39.710
39.500
39.291
39.083
38.873
31, AL129
170 160 150 140 130 120 110 100 90 80 70 60 50 40 30 ppm

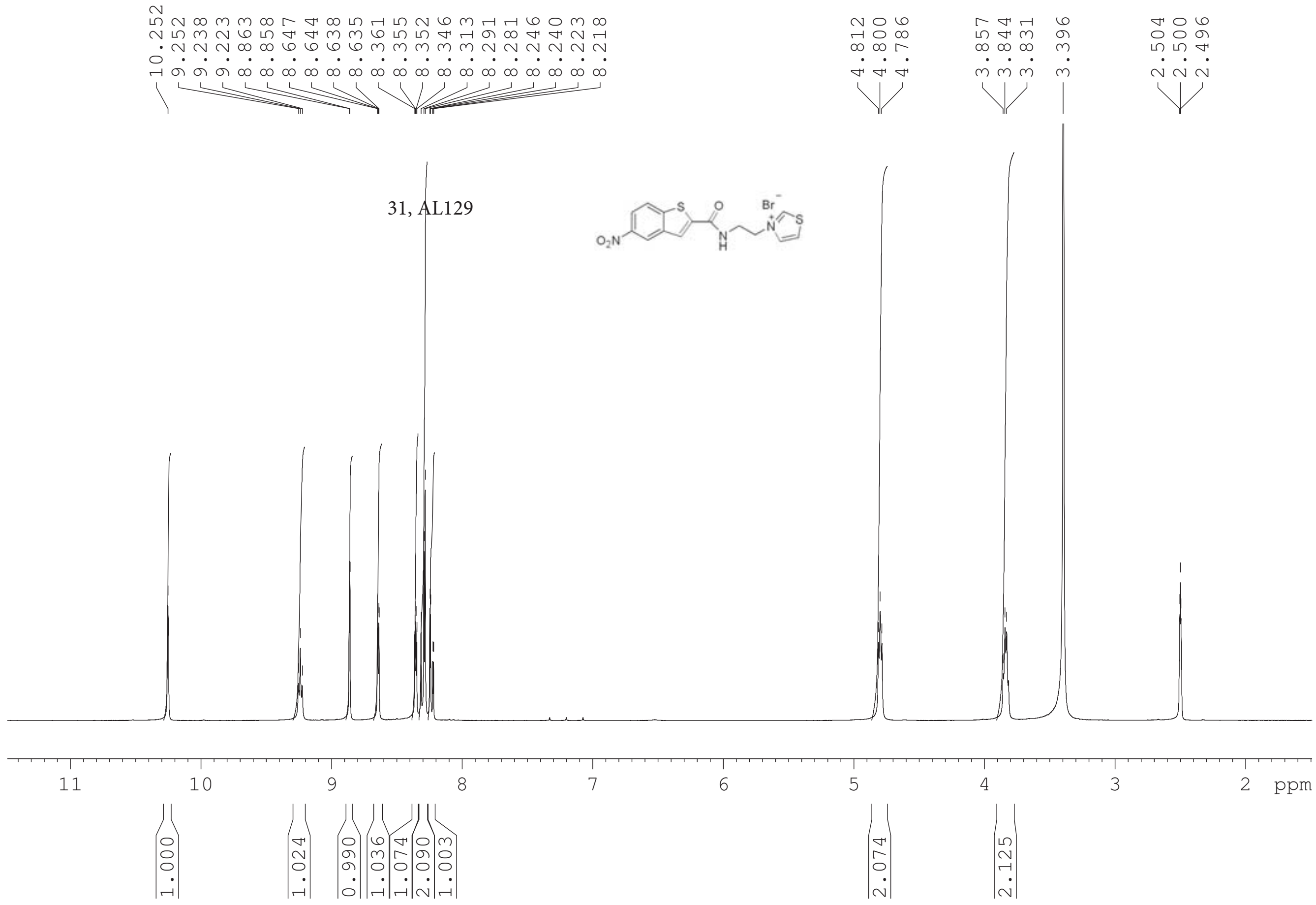
10.252
9.252
9.238
9.223
8.863
8.858
8.647
8.644
8.638
8.635
8.361
8.355
8.352
8.346
8.313
8.291
8.281
8.246
8.240
8.223
8.218
4.812
4.800
4.786
3.857
3.844
3.831
3.396
2.504
2.500
2.496
31, AL129
11 10 9 8 7 6 5 4 3 2 ppm
1.000
1.024
0.990
1.036
1.074
2.090
1.003
2.074
2.125

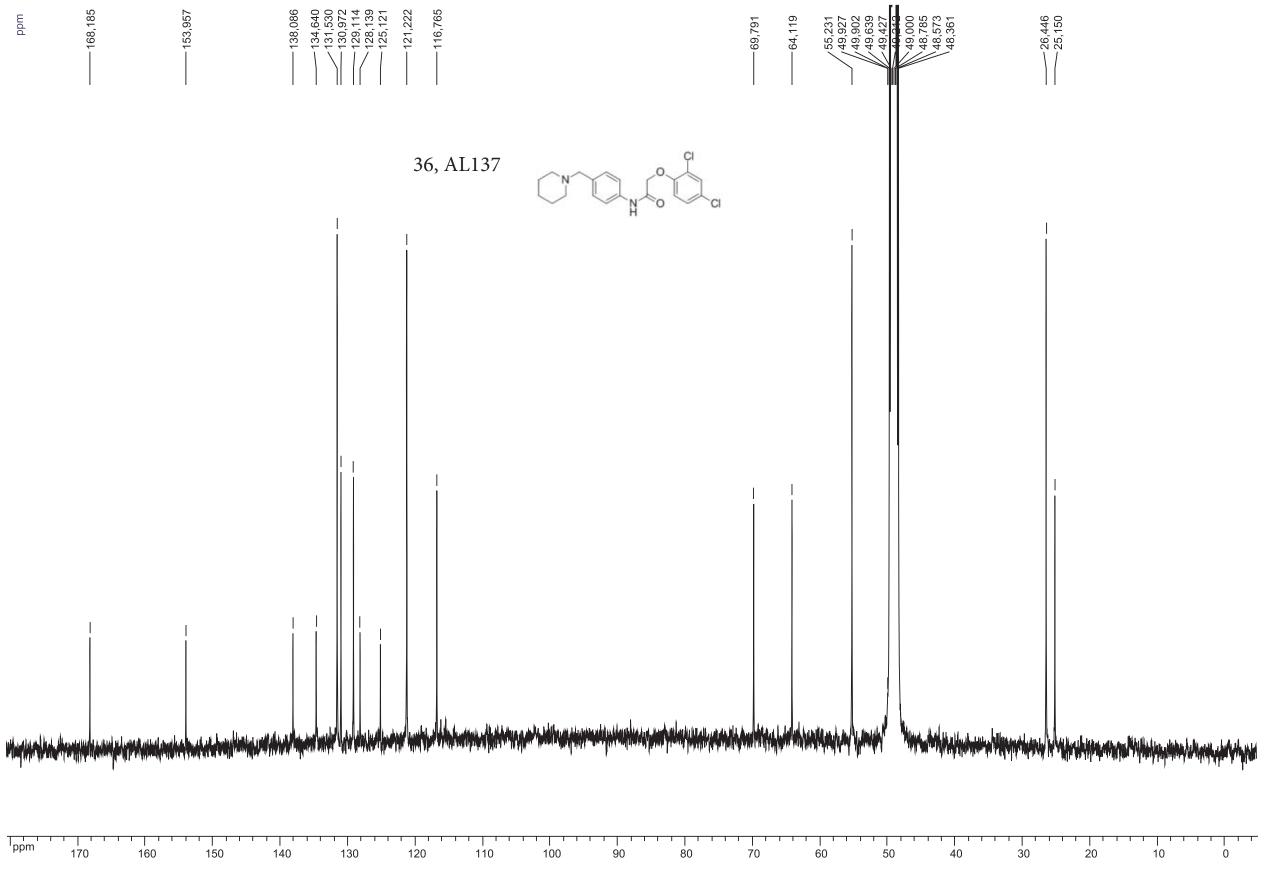
ppm
168,185
153,957
138,086
134,640
131,530
130,972
129,114
128,139
125,121
121,222
116,765
69,791
64,119
55,231
49,927
49,902
49,639
49,427
49,000
48,785
48,573
48,361
26,446
25,150
36, AL137
Cl
O
N
H
O
Cl
ppm
170
160
150
140
130
120
110
100
90
80
70
60
50
40
30
20
10
0

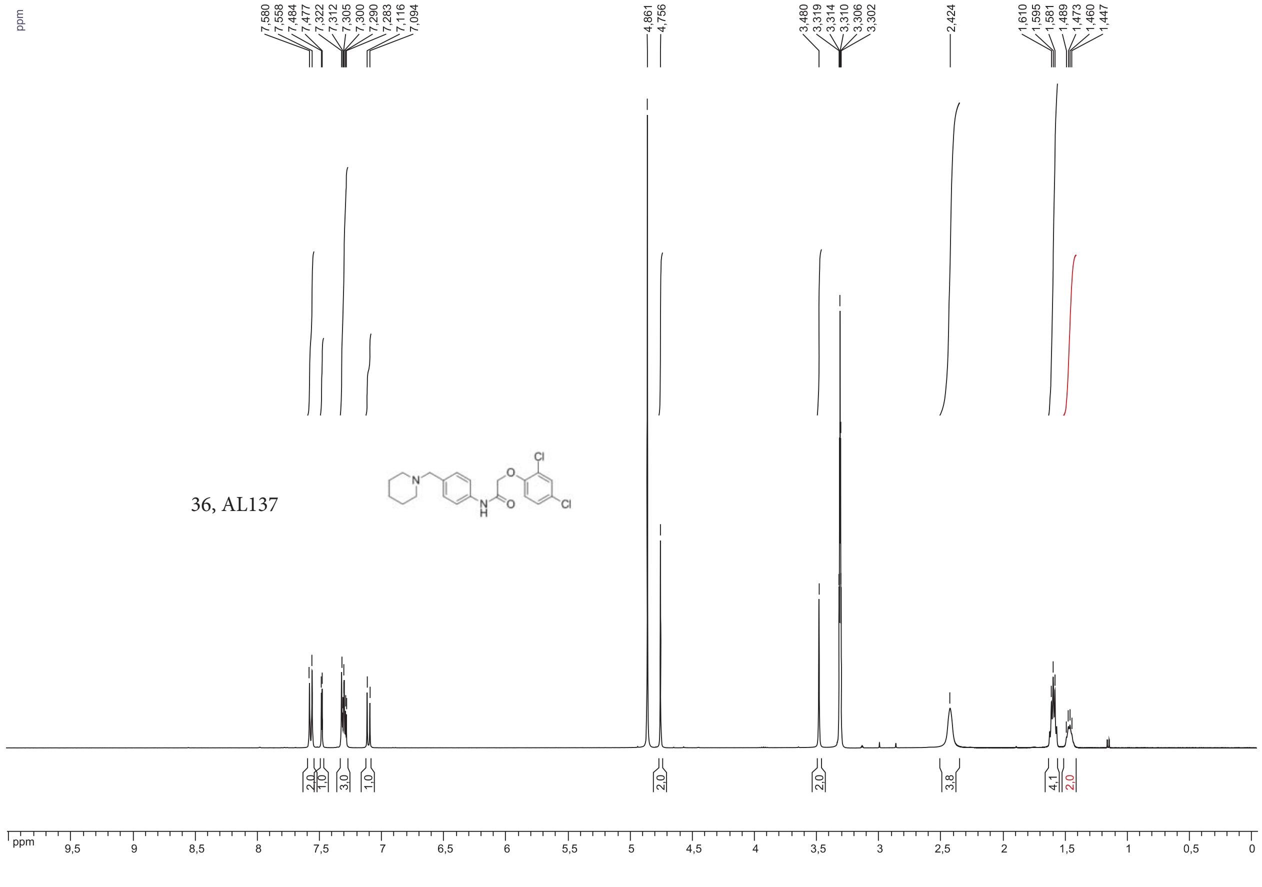
ppm
7,580
7,558
7,484
7,477
7,322
7,312
7,305
7,300
7,290
7,283
7,116
7,094
4,861
4,756
3,480
3,319
3,314
3,310
3,306
3,302
2,424
1,610
1,595
1,581
1,489
1,473
1,460
1,447
36, AL137
N
Cl
O
N
H
O
Cl
2,0
1,0
3,0
1,0
2,0
2,0
3,8
4,1
2,0
ppm
9,5
9
8,5
8
7,5
7
6,5
6
5,5
5
4,5
4
3,5
3
2,5
2
1,5
1
0,5
0

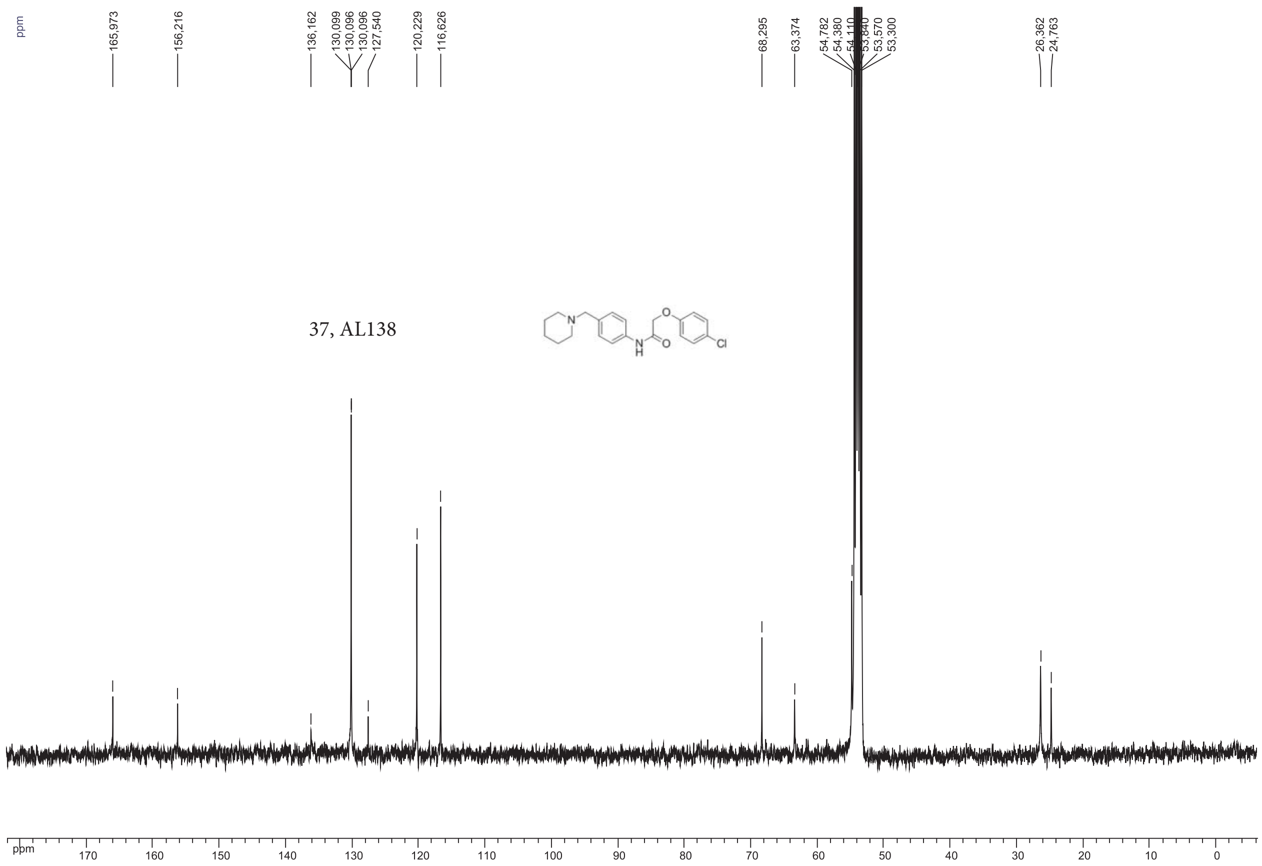
ppm
165,973
156,216
136,162
130,099
130,096
130,096
127,540
120,229
116,626
68,295
63,374
54,782
54,380
54,110
53,570
53,300
26,362
24,763
37, AL138
Cl
N
H
O
ppm
170
160
150
140
130
120
110
100
90
80
70
60
50
40
30
20
10
0

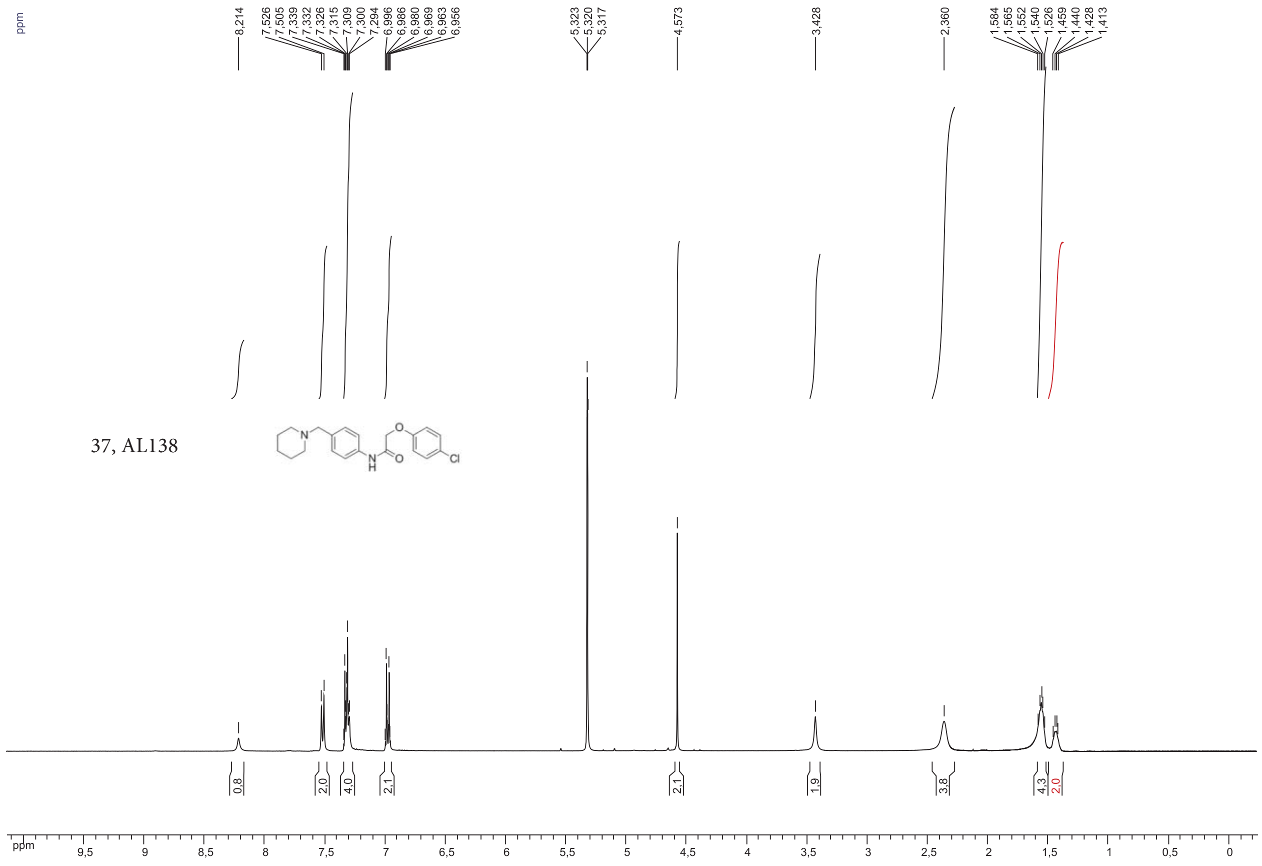
ppm
8,214
7,526
7,505
7,339
7,332
7,326
7,315
7,309
7,300
7,294
6,996
6,986
6,980
6,969
6,963
6,956
5,323
5,320
5,317
4,573
3,428
2,360
1,584
1,565
1,552
1,540
1,526
1,459
1,440
1,428
1,413
37, AL138
Cl
N
H
O
0,8
2,0
4,0
2,1
2,1
1,9
3,8
4,3
2,0
ppm
9,5
9
8,5
8
7,5
7
6,5
6
5,5
5
4,5
4
3,5
3
2,5
2
1,5
1
0,5
0

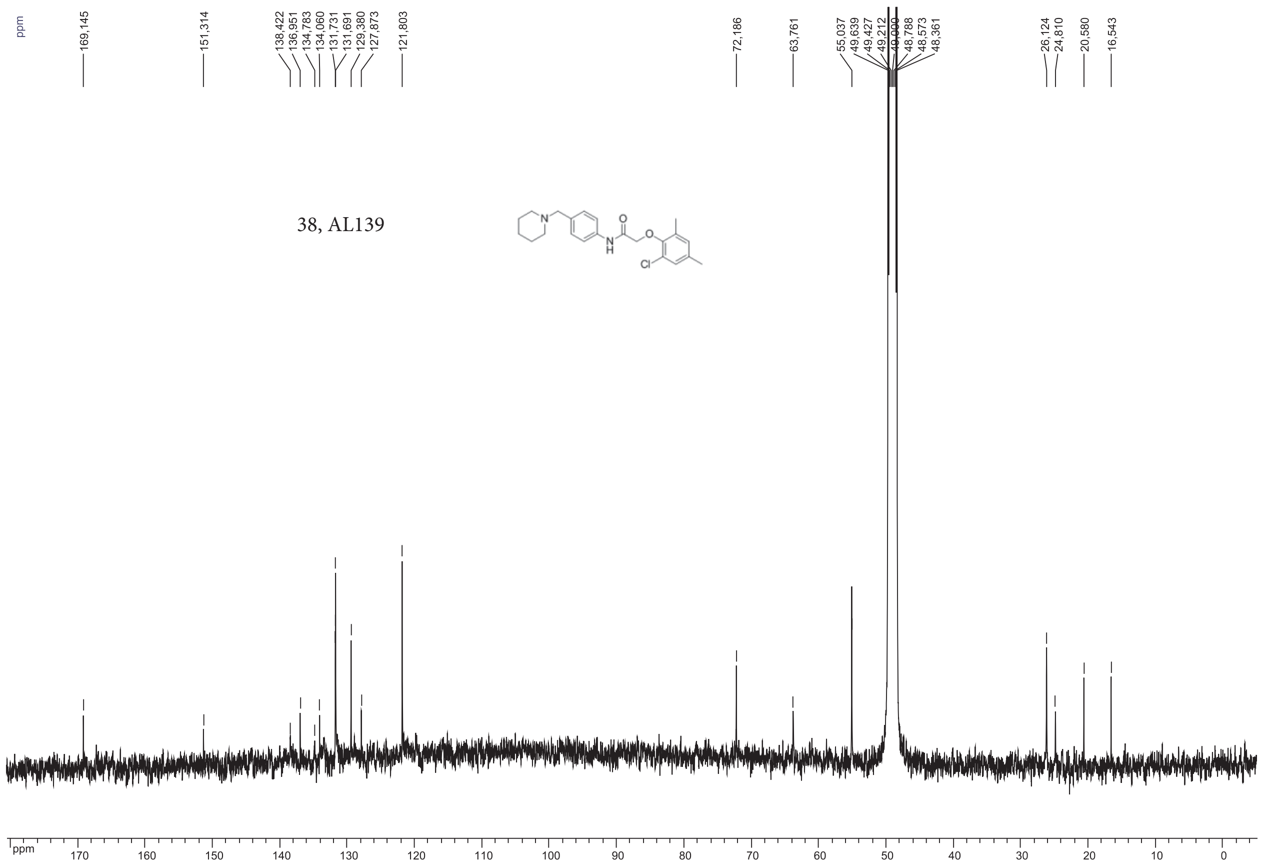

38, AL139
Cl
N
H
O
ppm
169,145
151,314
138,422
136,951
134,783
134,060
131,731
131,691
129,380
127,873
121,803
72,186
63,761
55,037
49,639
49,427
49,212
48,788
48,573
48,361
26,124
24,810
20,580
16,543

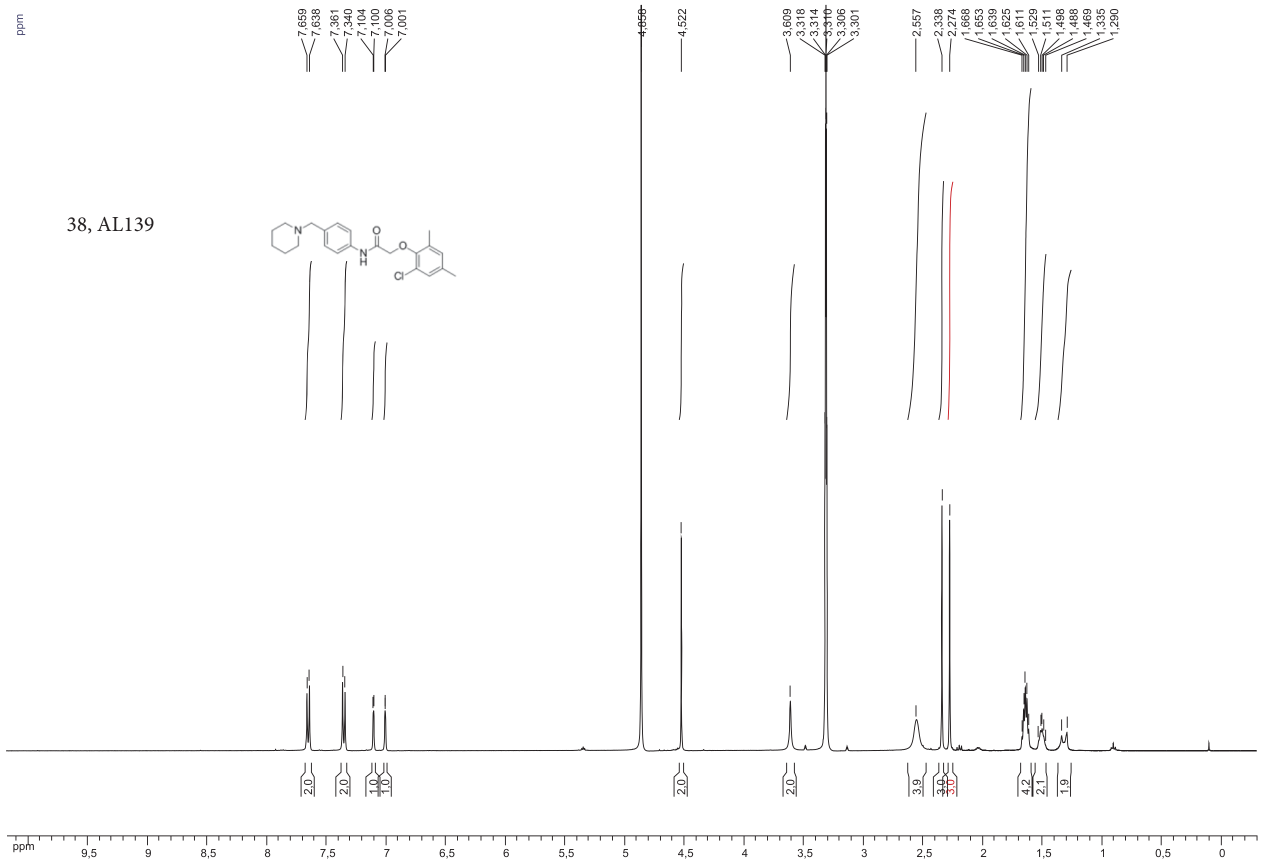

38, AL139
Cl
N
H
O
ppm
7,659
7,638
7,361
7,340
7,104
7,100
7,006
7,001
4,522
3,609
3,318
3,314
3,310
3,306
3,301
2,557
2,338
2,274
1,668
1,653
1,639
1,625
1,611
1,529
1,511
1,498
1,488
1,469
1,335
1,290
2,0
2,0
1,0
1,0
2,0
2,0
3,9
3,0
3,0
4,2
2,1
1,9

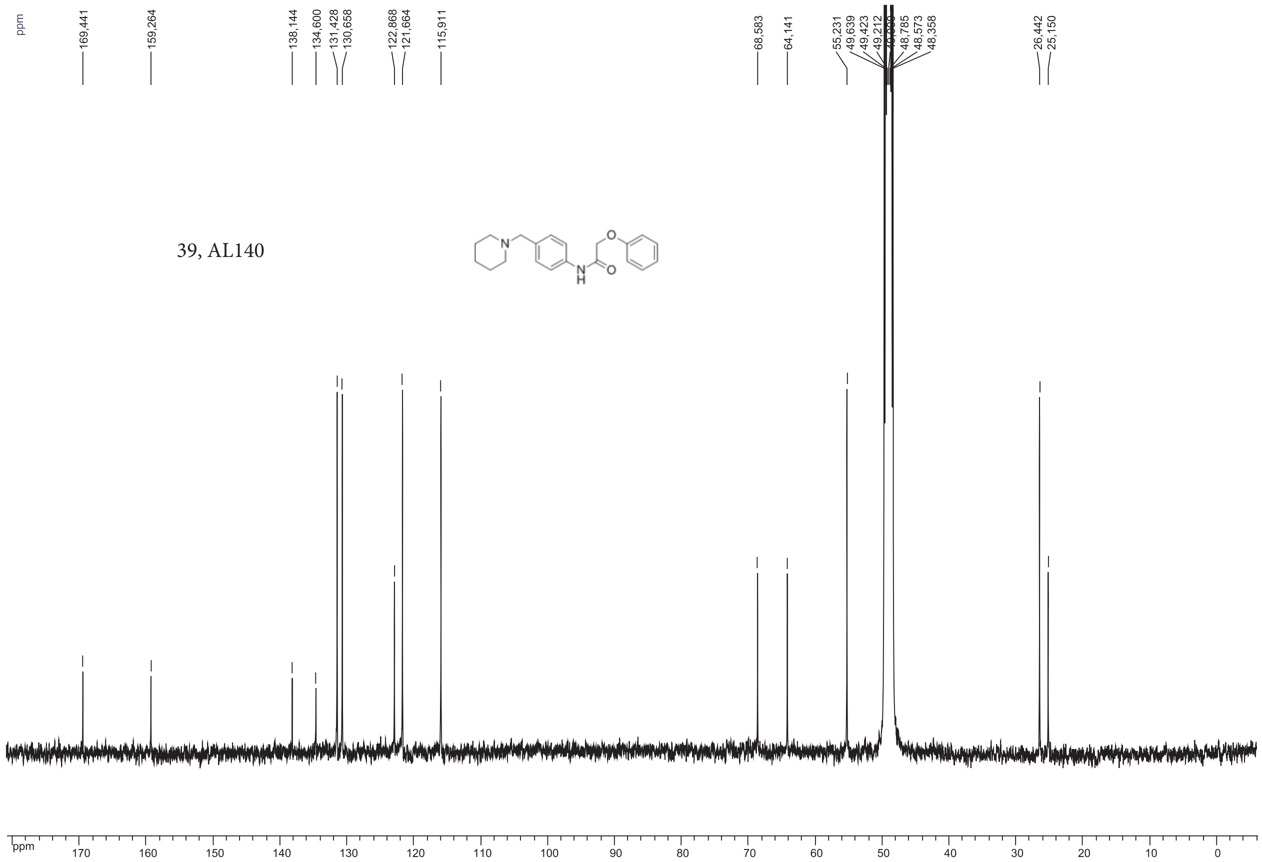
39, AL140
ppm
169,441
159,264
138,144
134,600
131,428
130,658
122,868
121,664
115,911
68,583
64,141
55,231
49,639
49,423
49,212
[illegible]
48,785
48,573
48,358
26,442
25,150
N
O
N
H
O
ppm 170 160 150 140 130 120 110 100 90 80 70 60 50 40 30 20 10 0

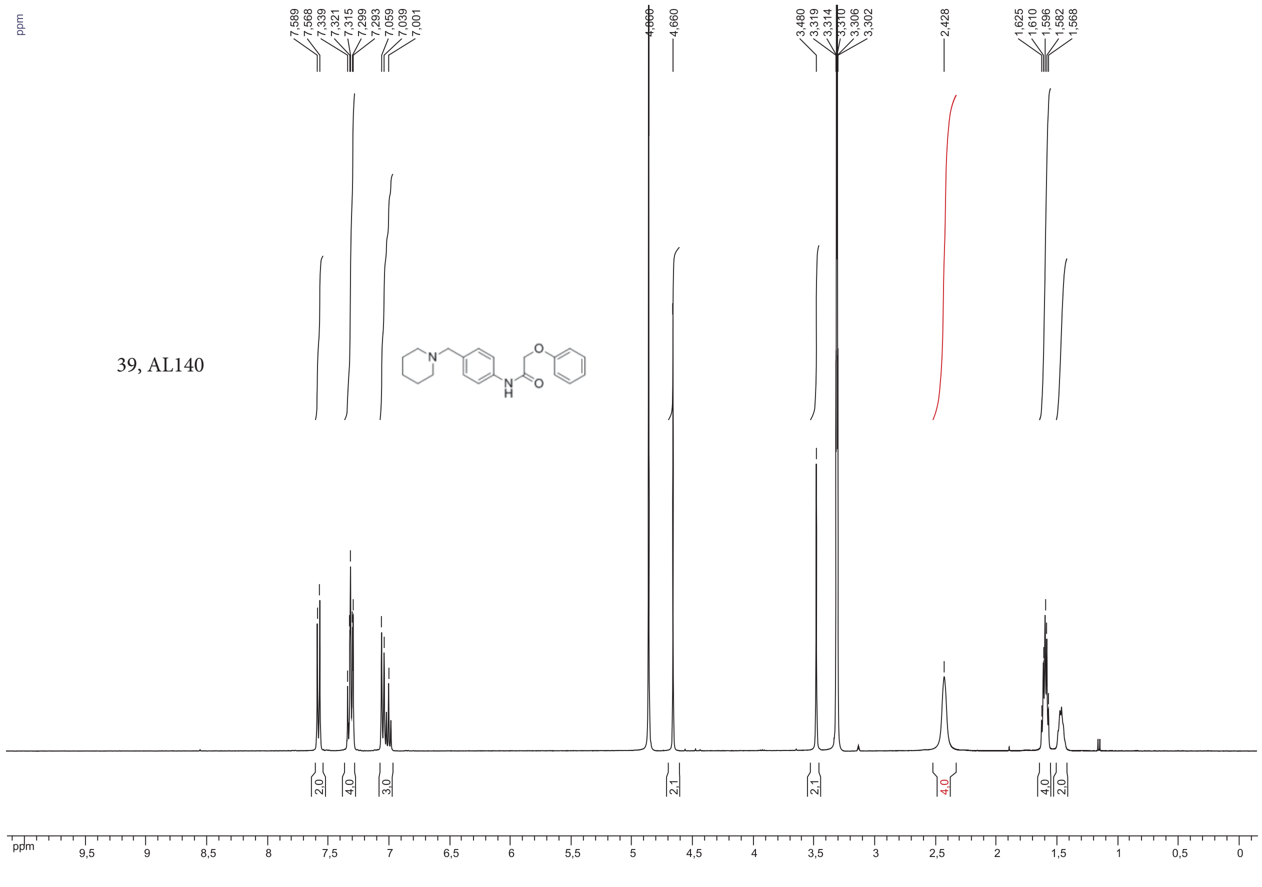
39, AL140
ppm
7,589
7,568
7,339
7,321
7,315
7,299
7,293
7,059
7,039
7,001
4,660
3,480
3,319
3,314
3,310
3,306
3,302
2,428
1,625
1,610
1,596
1,582
1,568
N
O
N
H
O
2,0
4,0
3,0
2,1
2,1
4,0
4,0
2,0
ppm 9,5 9 8,5 8 7,5 7 6,5 6 5,5 5 4,5 4 3,5 3 2,5 2 1,5 1 0,5 0

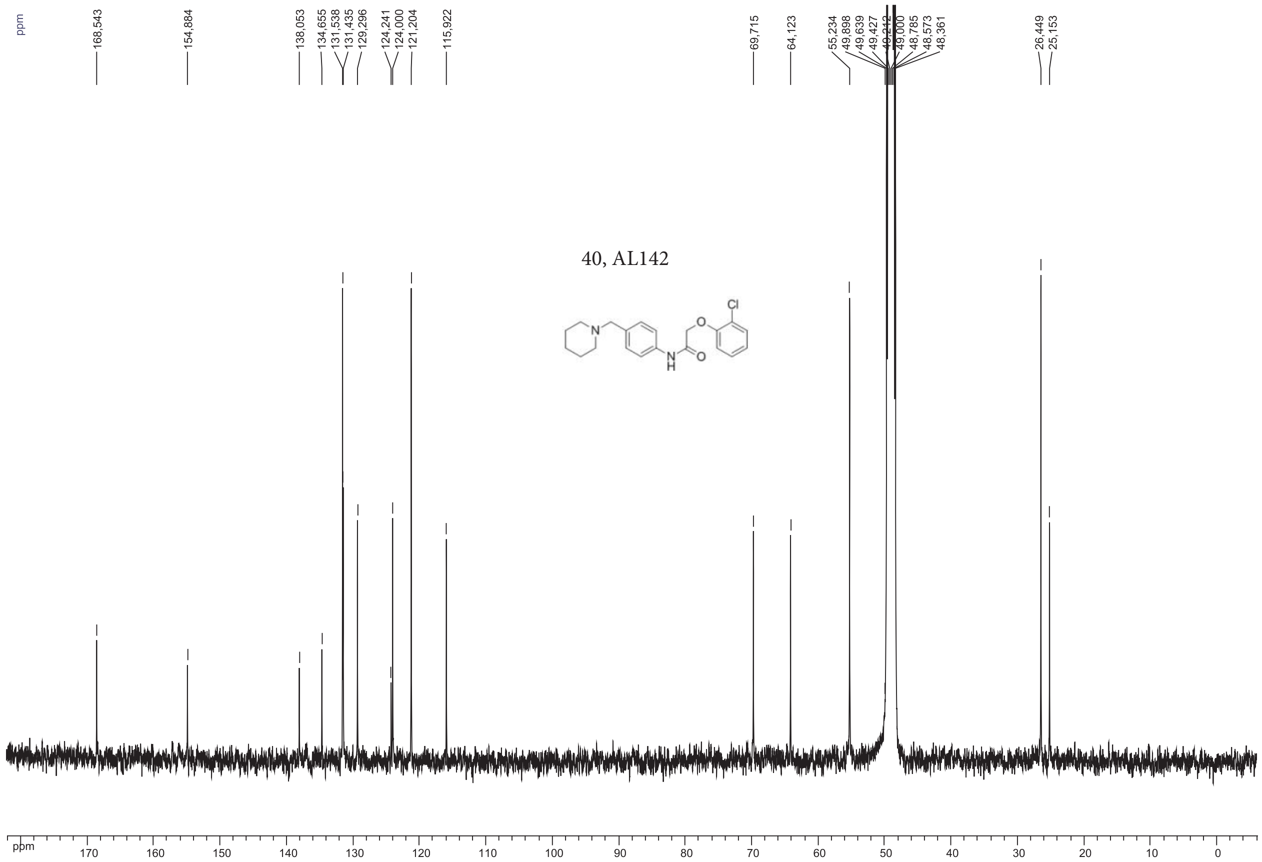
40, AL142
ppm
168,543
154,884
138,053
134,655
131,538
131,435
129,296
124,241
124,000
121,204
115,922
69,715
64,123
55,234
49,898
49,639
49,427
49,212
49,000
48,785
48,573
48,361
26,449
25,153
N
O
Cl
N
H
O
ppm 170 160 150 140 130 120 110 100 90 80 70 60 50 40 30 20 10 0

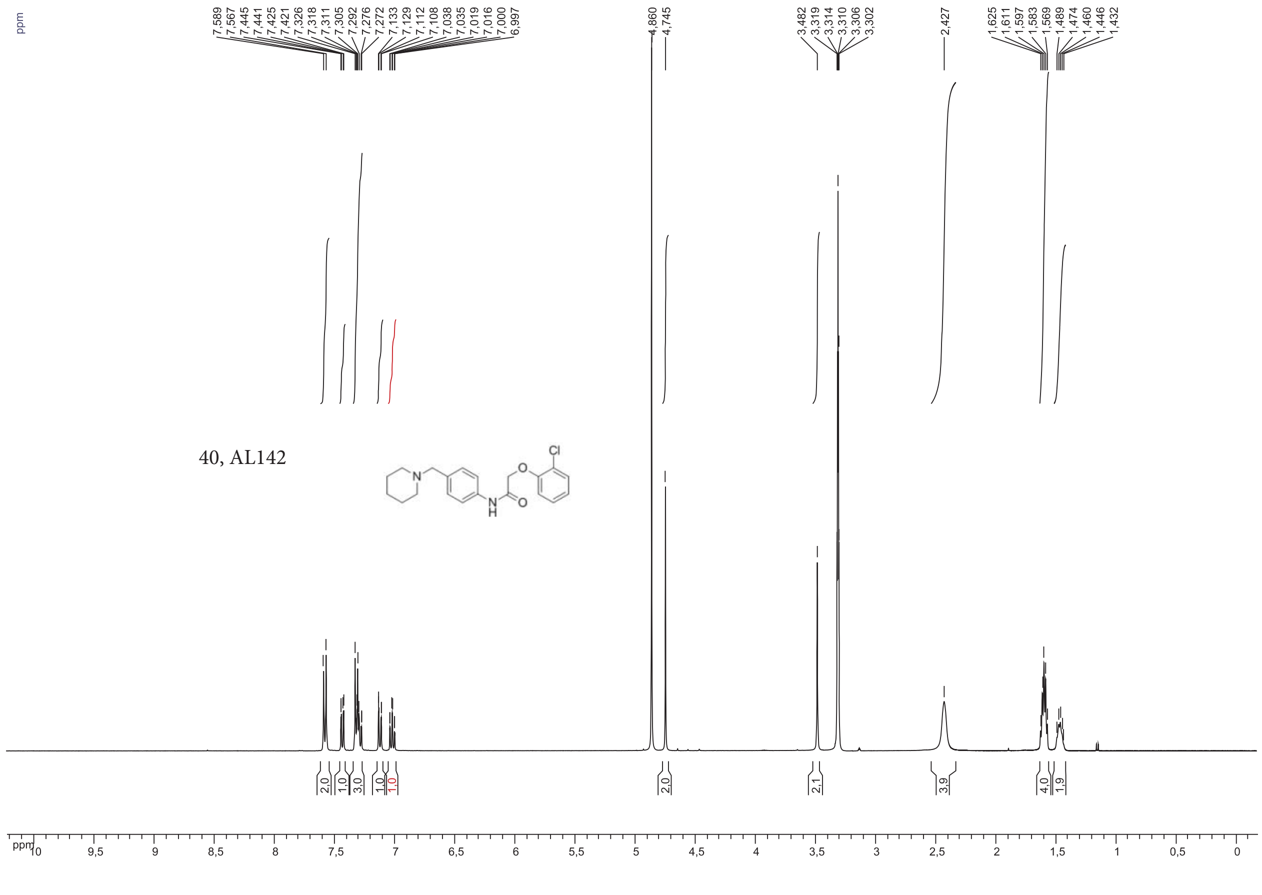
40, AL142
ppm
7,589
7,567
7,445
7,441
7,425
7,421
7,326
7,318
7,311
7,305
7,292
7,276
7,272
7,133
7,129
7,112
7,108
7,038
7,035
7,019
7,016
7,000
6,997
4,860
4,745
3,482
3,319
3,314
3,310
3,306
3,302
2,427
1,625
1,611
1,597
1,583
1,569
1,489
1,474
1,460
1,446
1,432
N
O
Cl
N
H
O
2,0
1,0
3,0
1,0
1,0
2,0
2,1
3,9
4,0
1,9
ppm 10 9,5 9 8,5 8 7,5 7 6,5 6 5,5 5 4,5 4 3,5 3 2,5 2 1,5 1 0,5 0

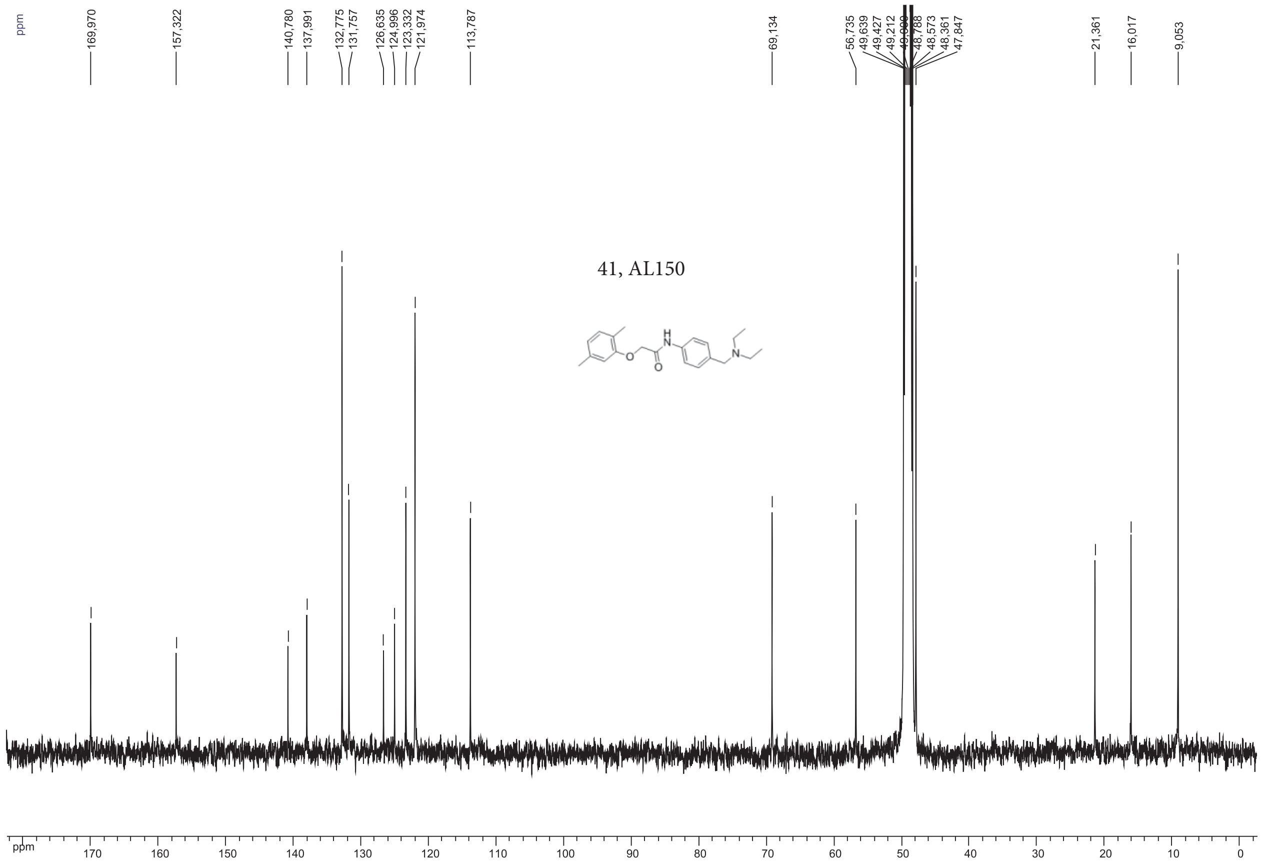
ppm
169,970
157,322
140,780
137,991
132,775
131,757
126,635
124,996
123,332
121,974
113,787
69,134
56,735
49,639
49,427
49,212
48,788
48,573
48,361
47,847
21,361
16,017
9,053
41, AL150
ppm
170
160
150
140
130
120
110
100
90
80
70
60
50
40
30
20
10
0

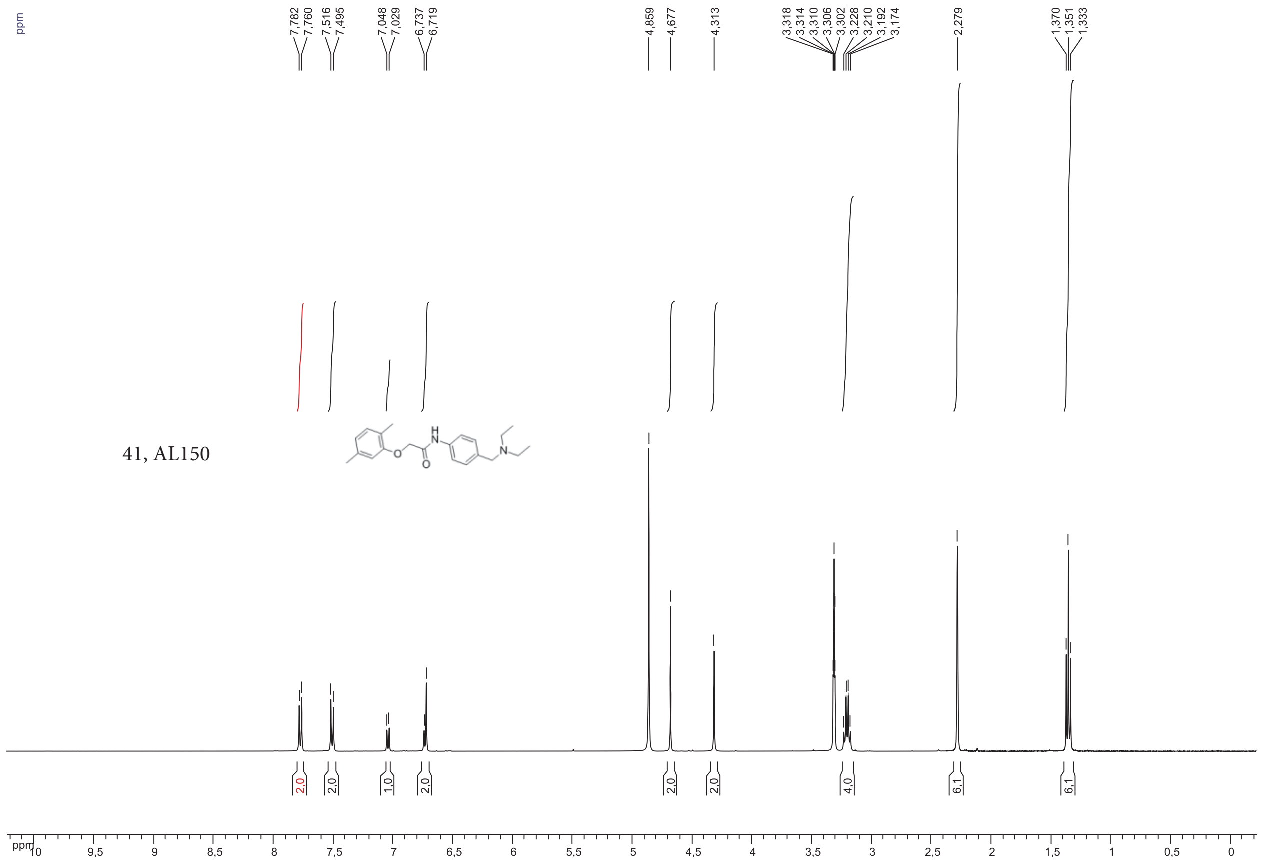
ppm
7,782
7,760
7,516
7,495
7,048
7,029
6,737
6,719
4,859
4,677
4,313
3,318
3,314
3,310
3,306
3,302
3,228
3,210
3,192
3,174
2,279
1,370
1,351
1,333
41, AL150
2,0
2,0
1,0
2,0
2,0
2,0
4,0
6,1
6,1
ppm
10
9,5
9
8,5
8
7,5
7
6,5
6
5,5
5
4,5
4
3,5
3
2,5
2
1,5
1
0,5
0

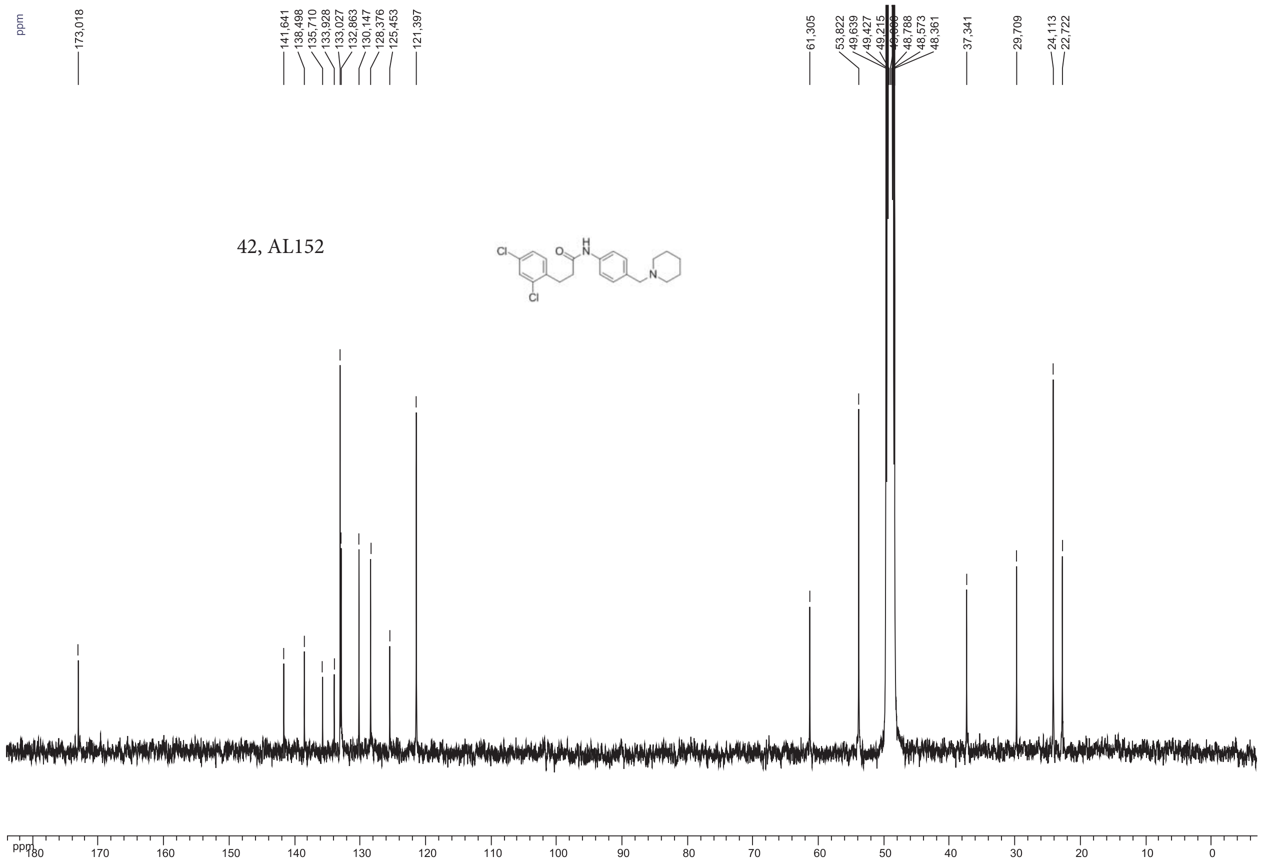
ppm
173,018
141,641
138,498
135,710
133,928
133,027
132,863
130,147
128,376
125,453
121,397
61,305
53,822
49,639
49,427
49,215
48,788
48,573
48,361
37,341
29,709
24,113
22,722
42, AL152
Cl
O
N
H
Cl
N
ppm
180
170
160
150
140
130
120
110
100
90
80
70
60
50
40
30
20
10
0

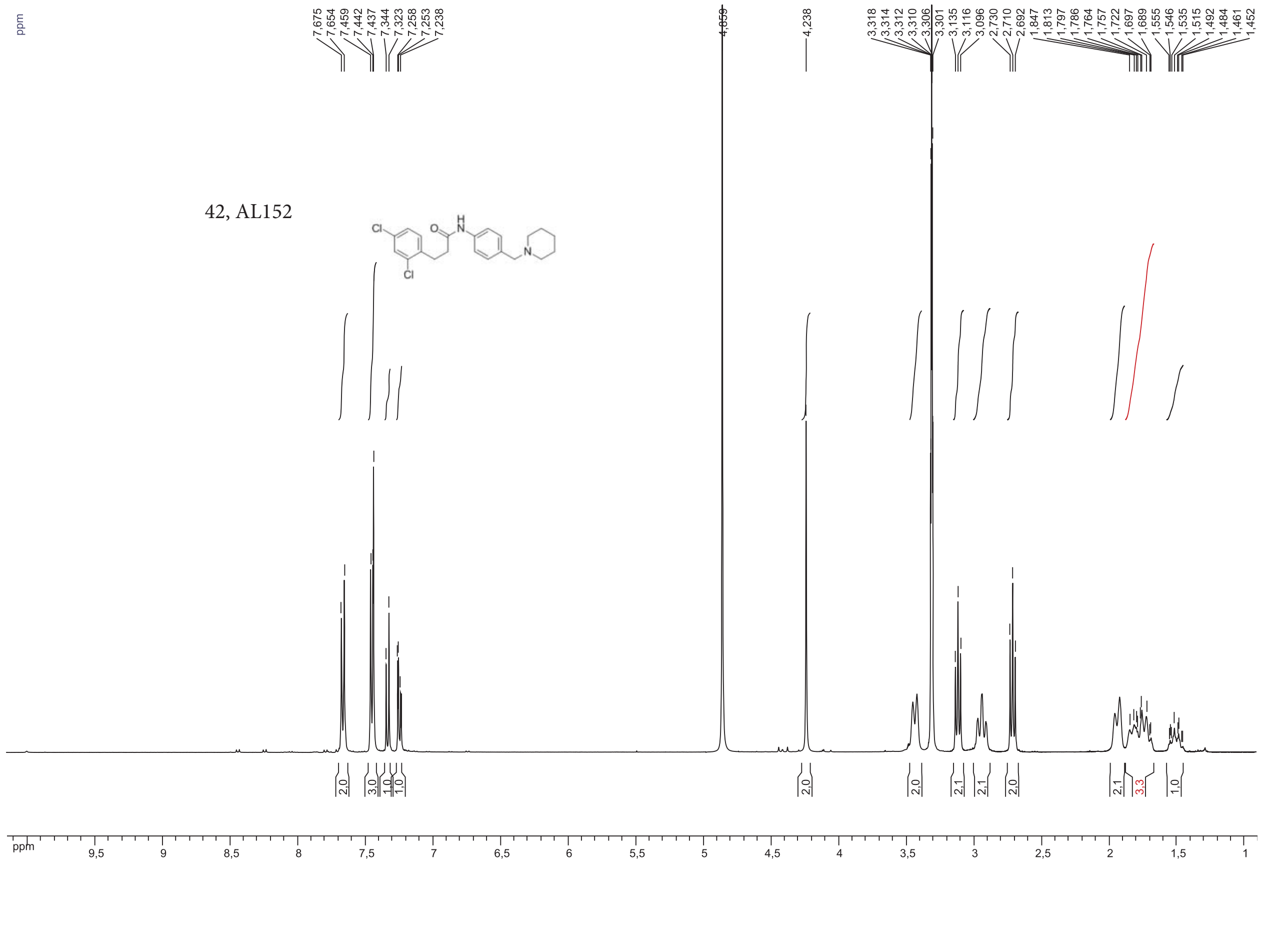
ppm
7,675
7,654
7,459
7,442
7,437
7,344
7,323
7,258
7,253
7,238
4,859
4,238
3,318
3,314
3,312
3,310
3,306
3,301
3,135
3,116
3,096
2,730
2,710
2,692
1,847
1,813
1,797
1,786
1,764
1,757
1,722
1,697
1,689
1,555
1,546
1,535
1,515
1,492
1,484
1,461
1,452
42, AL152
Cl
O
N
H
Cl
N
2,0
3,0
1,0
1,0
2,0
2,0
2,1
2,1
2,0
2,1
3,3
1,0
ppm
9,5
9
8,5
8
7,5
7
6,5
6
5,5
5
4,5
4
3,5
3
2,5
2
1,5
1

Graph

**Sample Name**	B07
**Application Name**	Pretech (Administrator)
**Method Name**	Analytical 1Grad 250mm
**Configuration Name**	Kemi Analytical ELSDNY
**Version**	9
**Data Instrument Name**	Contact
**Data Channel Name**	ELSD
**Notes**	
**Injection Number**	6

1, AL011

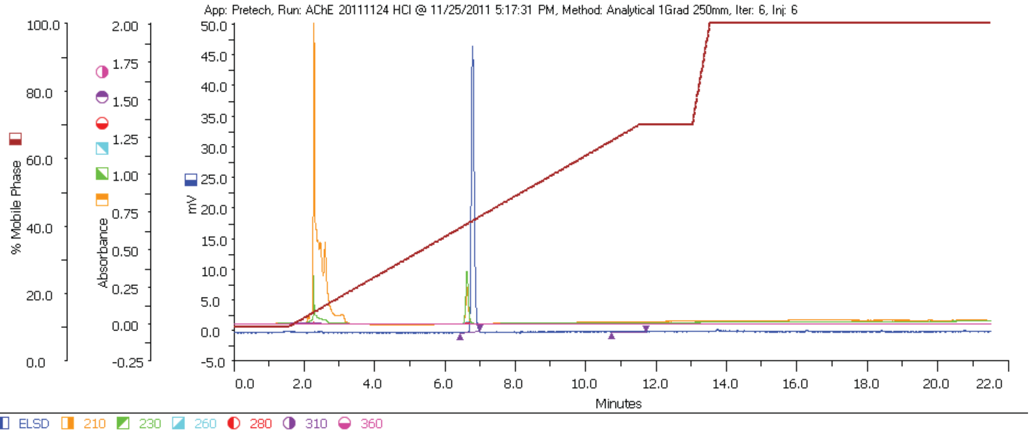

Graph

**Sample Name**	B09
**Application Name**	Pretech (Administrator)
**Method Name**	Analytical 1Grad 250mm
**Configuration Name**	Kemi Analytical ELSDNY
**Version**	9
**Data Instrument Name**	Contact
**Data Channel Name**	ELSD
**Notes**	
**Injection Number**	8

2, AL013

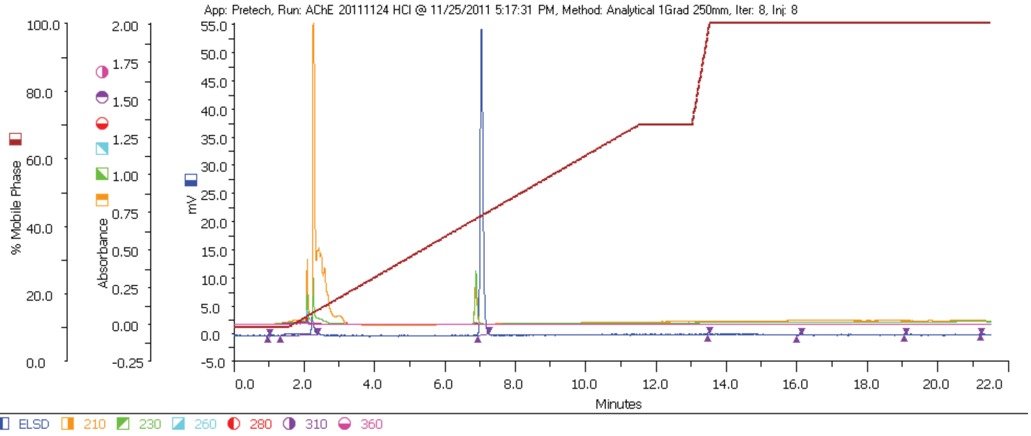

Graph

3, AL012

**Sample Name**	B08
**Application Name**	Pretech (Administrator)
**Method Name**	Analytical 1Grad 250mm
**Configuration Name**	Kemi Analytical ELSDNY
**Version**	9
**Data Instrument Name**	Contact
**Data Channel Name**	ELSD
**Notes**	
**Injection Number**	7

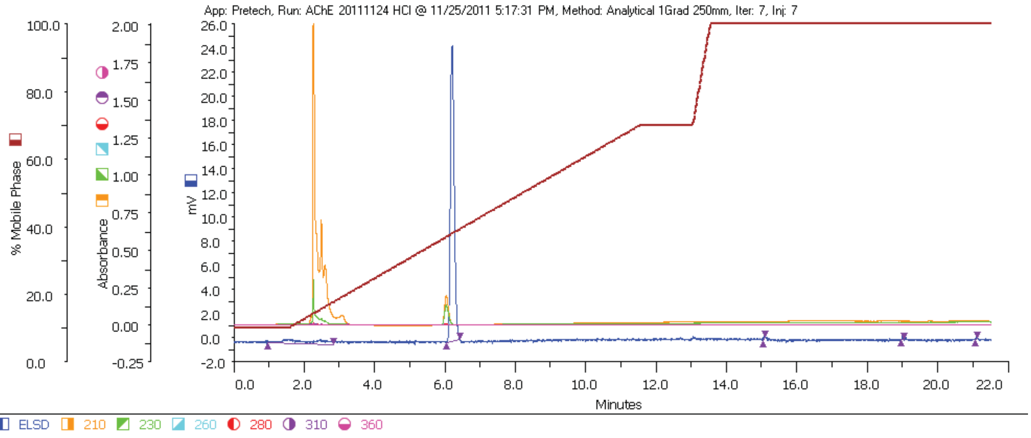

Graph

**Sample Name**	B03
**Application Name**	Pretech (Administrator)
**Method Name**	Analytical 1Grad 250mm
**Configuration Name**	Kemi Analytical ELSDNY
**Version**	9
**Data Instrument Name**	Contact
**Data Channel Name**	ELSD
**Notes**	
**Injection Number**	3

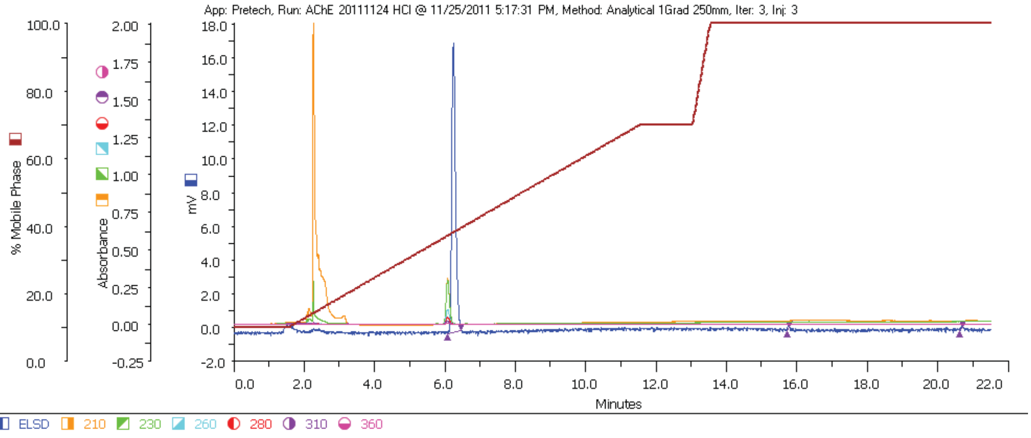

Graph

**Sample Name**	B04
**Application Name**	Pretech (Administrator)
**Method Name**	Analytical 1Grad 250mm
**Configuration Name**	Kemi Analytical ELSDNY
**Version**	9
**Data Instrument Name**	Contact
**Data Channel Name**	ELSD
**Notes**	
**Injection Number**	4

5, AL008

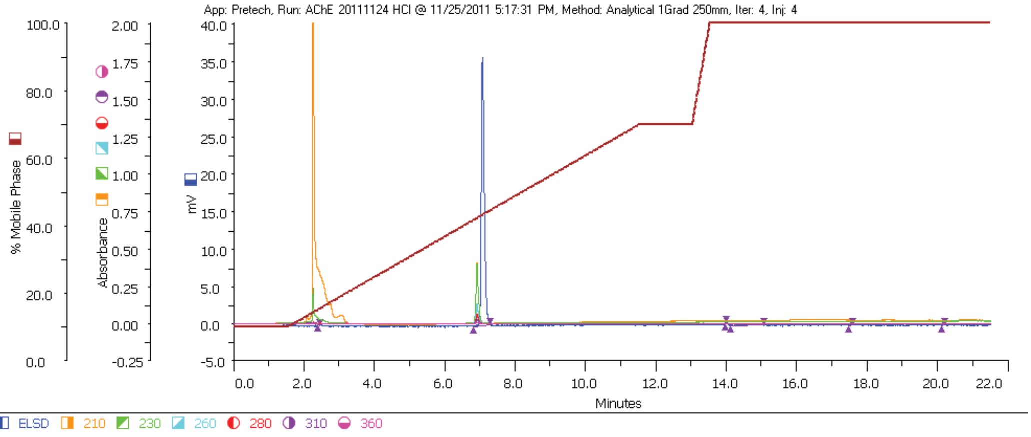

Graph

**Sample Name**	B02
**Application Name**	Pretech (Administrator)
**Method Name**	Analytical 1Grad 250mm
**Configuration Name**	Kemi Analytical ELSDNY
**Version**	9
**Data Instrument Name**	Contact
**Data Channel Name**	ELSD
**Notes**	
**Injection Number**	2

6, AL006

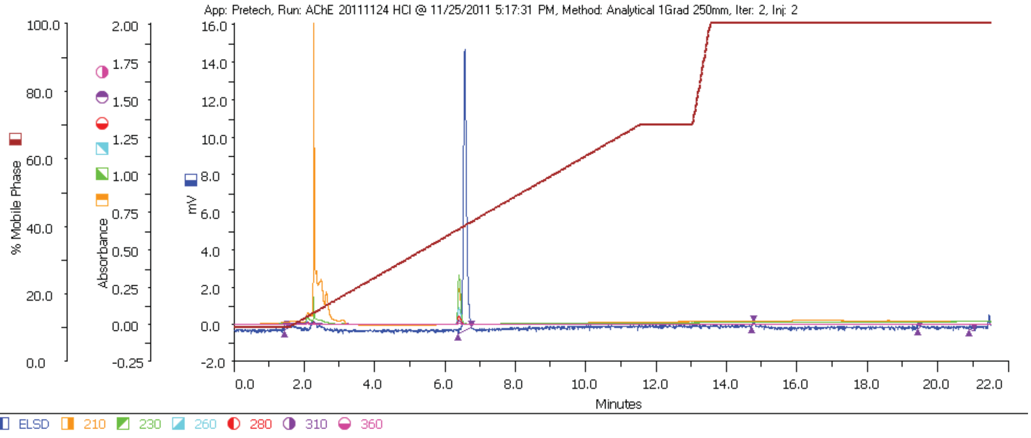

Graph

**Sample Name**	B11
**Application Name**	Pretech (Administrator)
**Method Name**	Analytical 1Grad 250mm
**Configuration Name**	Kemi Analytical ELSDNY
**Version**	9
**Data Instrument Name**	Contact
**Data Channel Name**	ELSD
**Notes**	
**Injection Number**	10

7, AL015

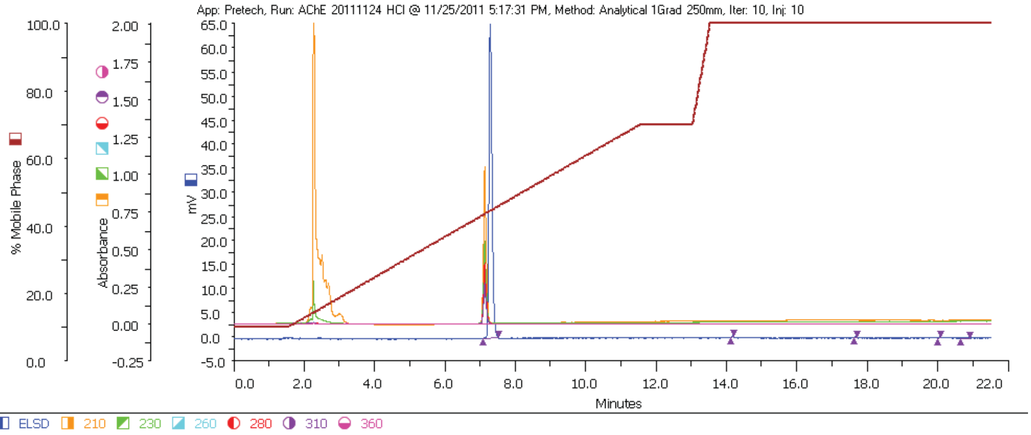

8, AL016

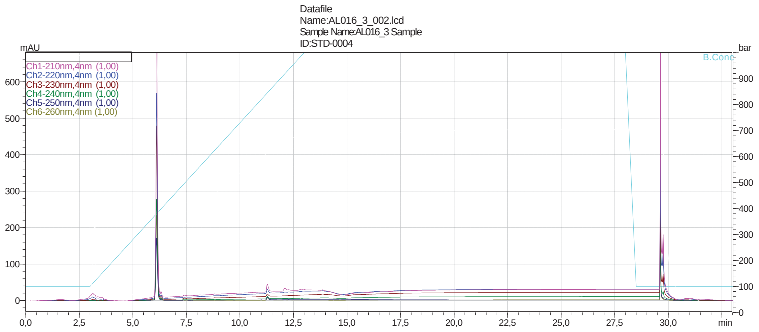

Graph

**Sample Name**	B01
**Application Name**	Pretech (Administrator)
**Method Name**	Analytical 1Grad 250mm
**Configuration Name**	Kemi Analytical ELSDNY
**Version**	9
**Data Instrument Name**	Contact
**Data Channel Name**	ELSD
**Notes**	
**Injection Number**	1

9, AL005

App: Pretech, Run: AChE 20111124 HCl @ 11/25/2011 5:17:31 PM, Method: Analytical 1Grad 250mm, Iter: 1, Inj: 1

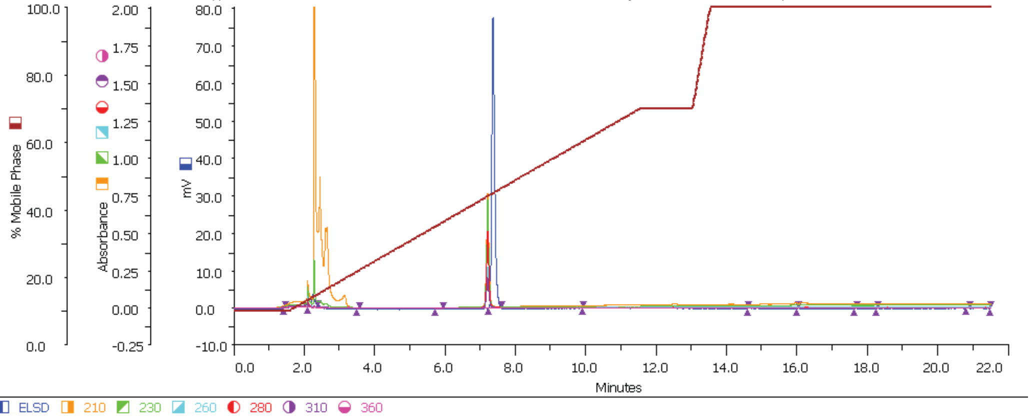

Graph

**Sample Name**	B10
**Application Name**	Pretech (Administrator)
**Method Name**	Analytical 1Grad 250mm
**Configuration Name**	Kemi Analytical ELSDNY
**Version**	9
**Data Instrument Name**	Contact
**Data Channel Name**	ELSD
**Notes**	
**Injection Number**	9

10, AL014

App: Pretech, Run: AChE 20111124 HCl @ 11/25/2011 5:17:31 PM, Method: Analytical 1Grad 250mm, Iter: 9, Inj: 9

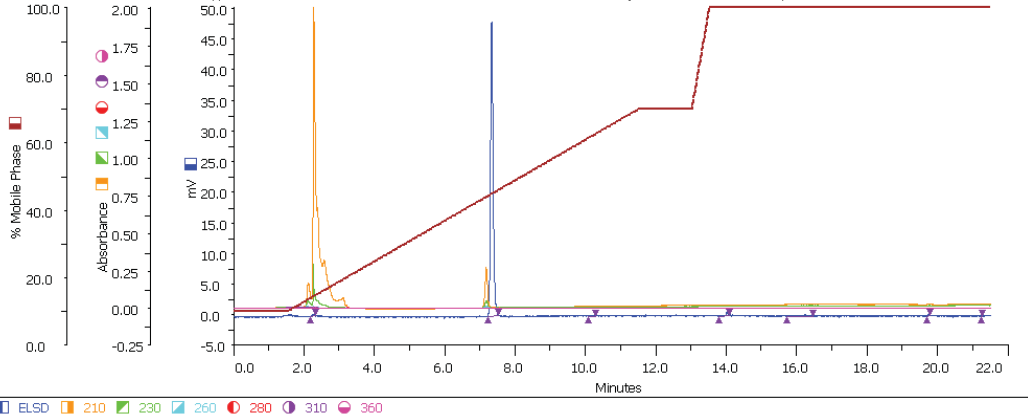

Graph

**Sample Name**	B05
**Application Name**	Pretech (Administrator)
**Method Name**	Analytical 1Grad 250mm
**Configuration Name**	Kemi Analytical ELSDNY
**Version**	9
**Data Instrument Name**	Contact
**Data Channel Name**	ELSD
**Notes**	
**Injection Number**	5

11, AL009

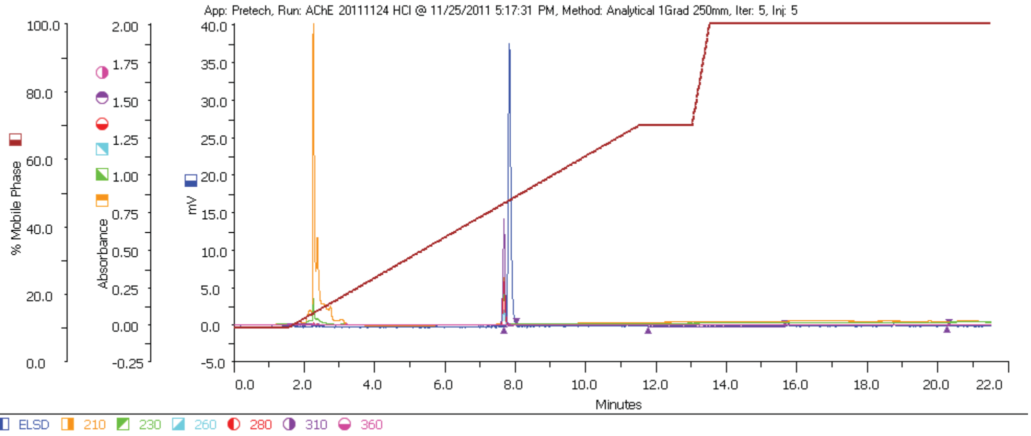

12, AL010

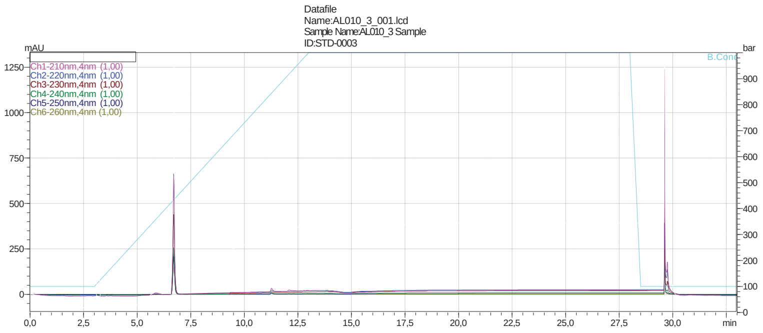

Graph

**Sample Name**	B13
**Application Name**	Pretech (Administrator)
**Method Name**	Analytical 1Grad 250mm
**Configuration Name**	Kemi Analytical ELSDNY
**Version**	9
**Data Instrument Name**	Contact
**Data Channel Name**	ELSD
**Notes**	
**Injection Number**	11

13, AL017

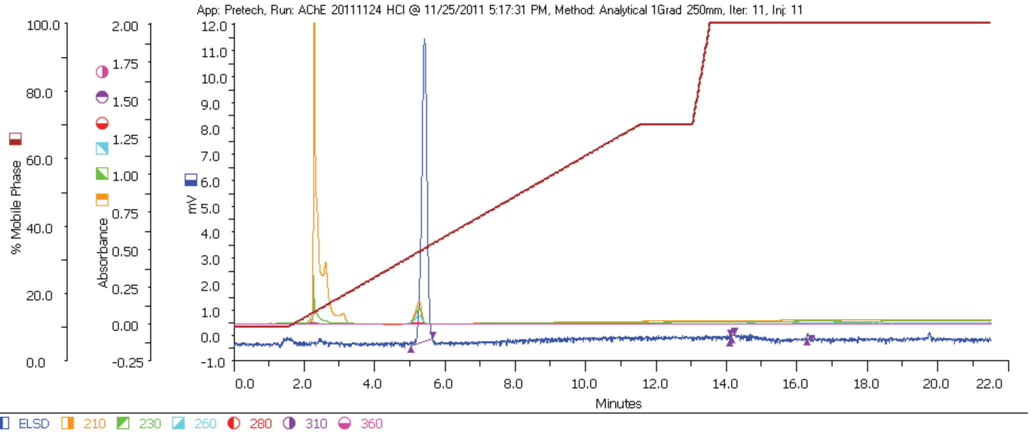

Graph

**Sample Name**	B18
**Application Name**	Pretech (Administrator)
**Method Name**	Analytical 1Grad 250mm
**Configuration Name**	Kemi Analytical ELSDNY
**Version**	9
**Data Instrument Name**	Contact
**Data Channel Name**	ELSD
**Notes**	
**Injection Number**	15

14, AL021

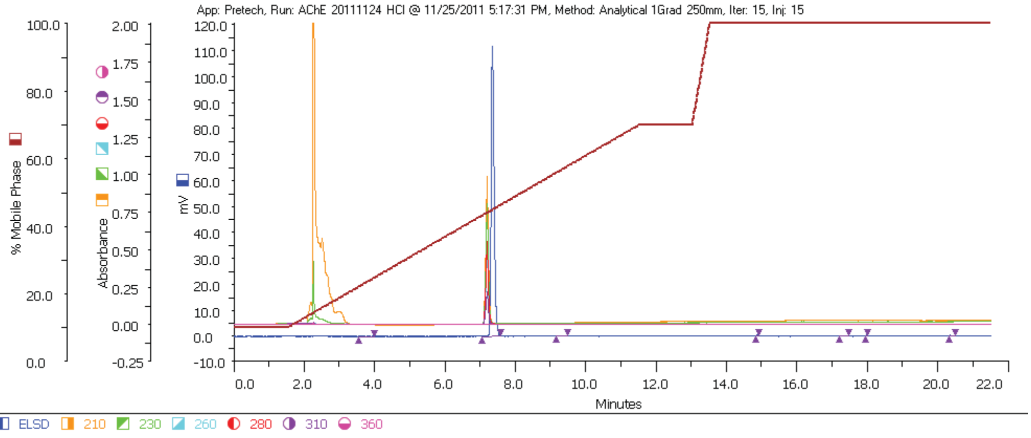

Graph

**Sample Name**	B19
**Application Name**	Pretech (Administrator)
**Method Name**	Analytical 1Grad 250mm
**Configuration Name**	Kemi Analytical ELSDNY
**Version**	9
**Data Instrument Name**	Contact
**Data Channel Name**	ELSD
**Notes**	
**Injection Number**	16

15, AL022

App: Pretech, Run: AChE 20111124 HCl @ 11/25/2011 5:17:31 PM, Method: Analytical 1Grad 250mm, Iter: 16, Inj: 16

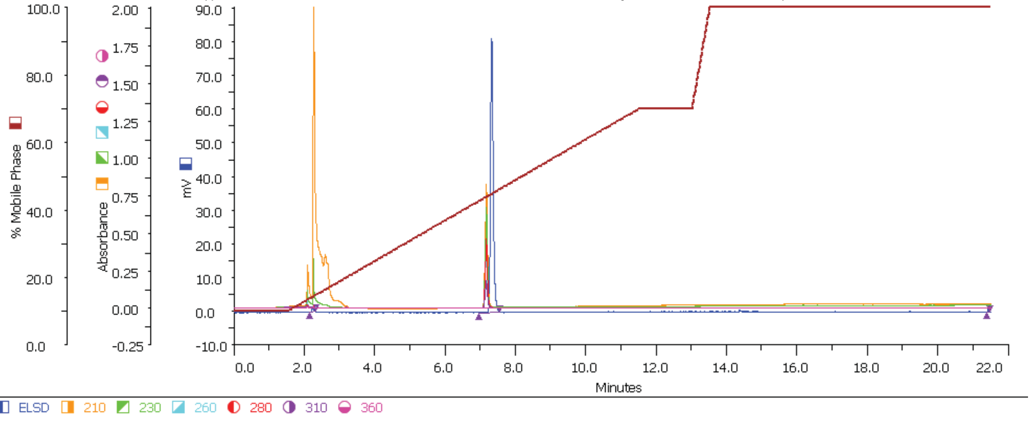

Graph

**Sample Name**	B15
**Application Name**	Pretech (Administrator)
**Method Name**	Analytical 1Grad 250mm
**Configuration Name**	Kemi Analytical ELSDNY
**Version**	9
**Data Instrument Name**	Contact
**Data Channel Name**	ELSD
**Notes**	
**Injection Number**	12

16, AL018

App: Pretech, Run: AChE 20111124 HCl @ 11/25/2011 5:17:31 PM, Method: Analytical 1Grad 250mm, Iter: 12, Inj: 12

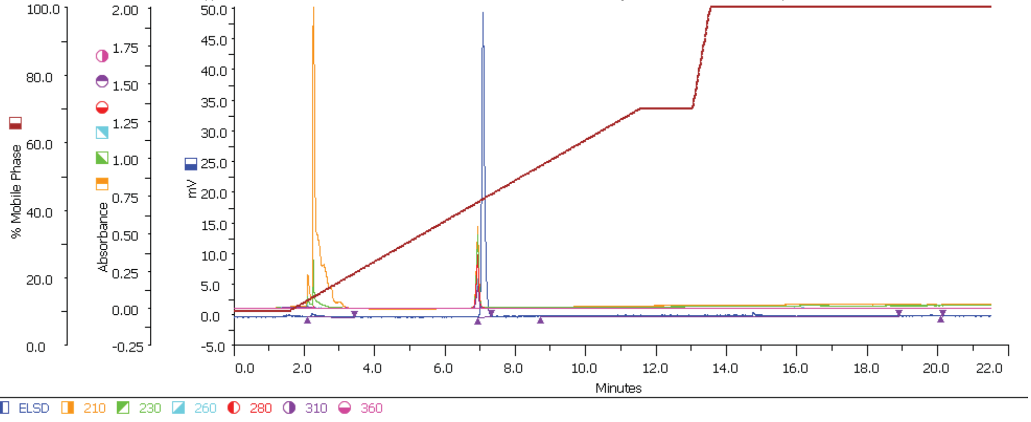

Graph

**Sample Name**	B17
**Application Name**	Pretech (Administrator)
**Method Name**	Analytical 1Grad 250mm
**Configuration Name**	Kemi Analytical ELSDNY
**Version**	9
**Data Instrument Name**	Contact
**Data Channel Name**	ELSD
**Notes**	
**Injection Number**	14

17, AL020

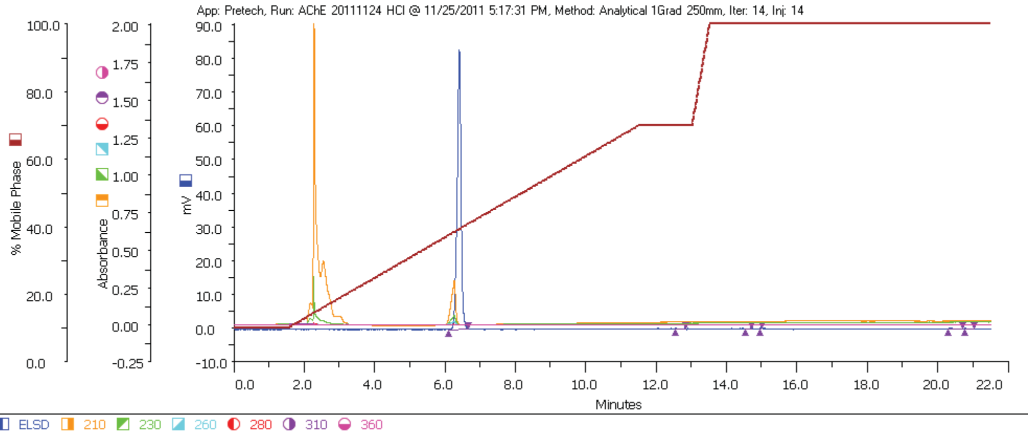

Graph

**Sample Name**	B16
**Application Name**	Pretech (Administrator)
**Method Name**	Analytical 1Grad 250mm
**Configuration Name**	Kemi Analytical ELSDNY
**Version**	9
**Data Instrument Name**	Contact
**Data Channel Name**	ELSD
**Notes**	
**Injection Number**	13

18, AL019

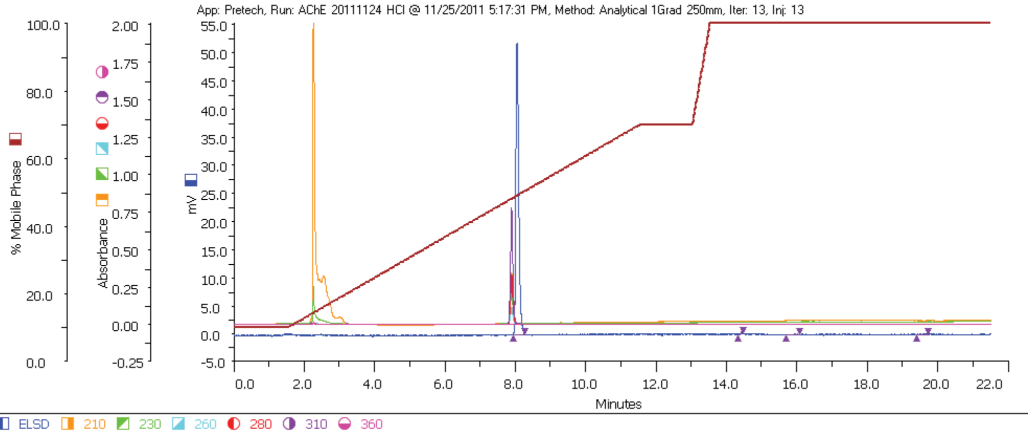

27, AL053

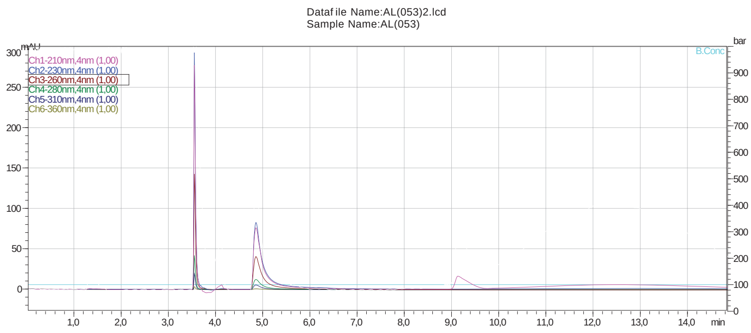

28, AL054

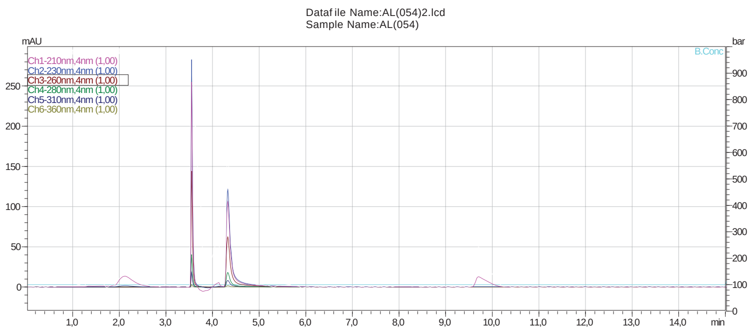

29, AL055

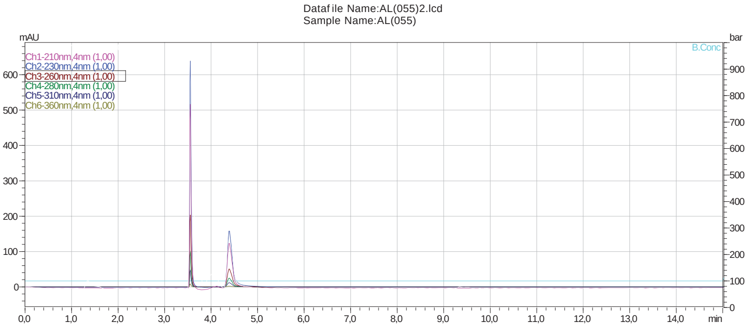

30, AL058

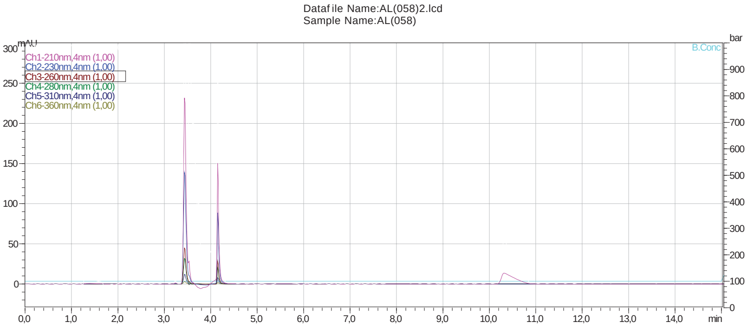

31, AL129

Dataf ile Name:AL(129)2.lcd
Sample Name:AL(129)

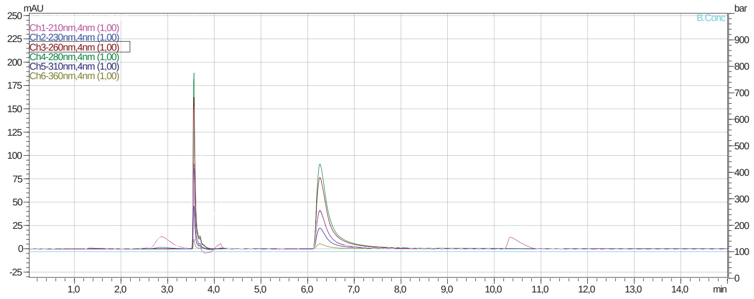

36, AL137

Dataf ile Name:AL137_002.lcd
Sample Name:AL137 Sample
ID:STD-0002

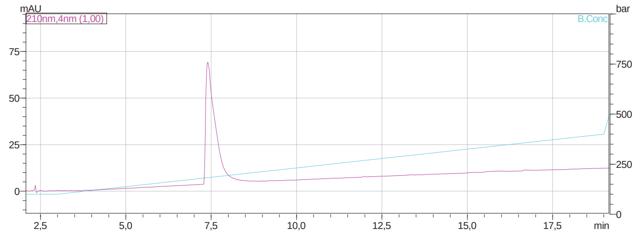

Dataf ile Name:AL137_002.lcd
Sample Name:AL137 Sample
ID:STD-0002

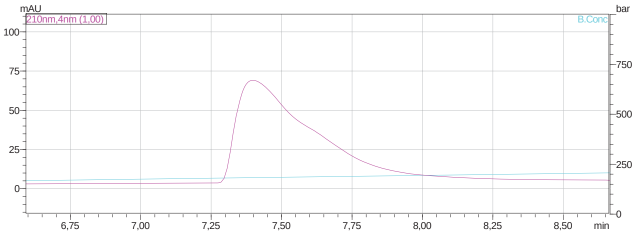

37, AL138

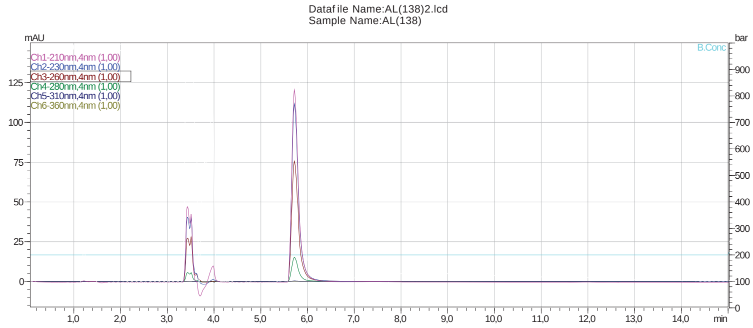

38, AL139

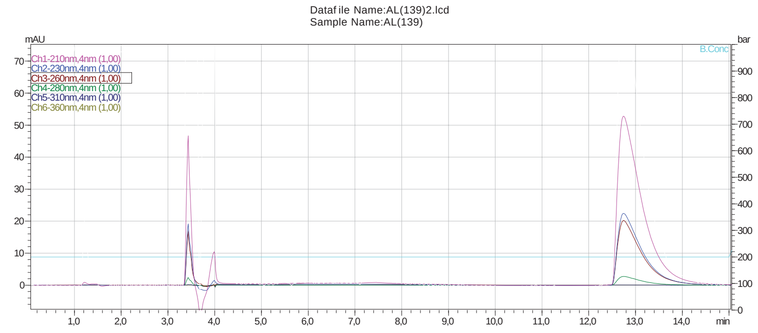

39, AL140

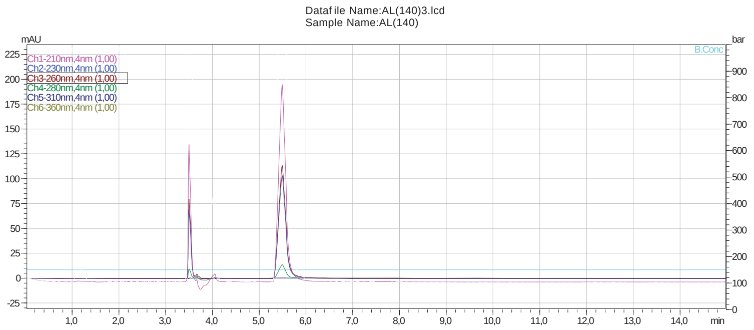

40, AL142

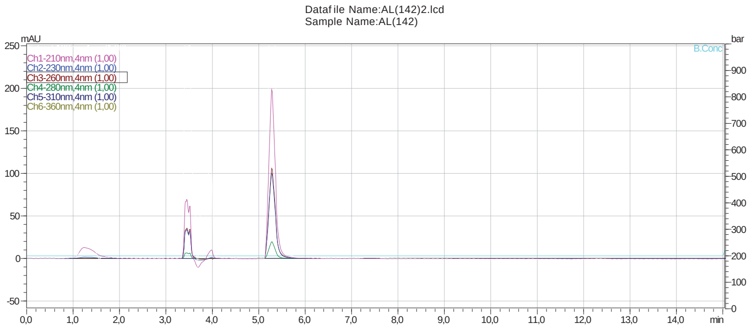

41, AL150

Dataf ile Name:AL150_009.lcd
Sample Name:AL150 Sample
ID:STD-0002

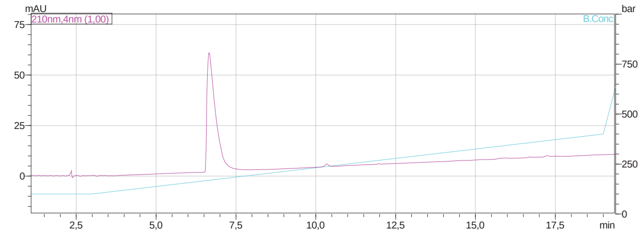

Dataf ile Name:AL150_009.lcd
Sample Name:AL150 Sample
ID:STD-0002

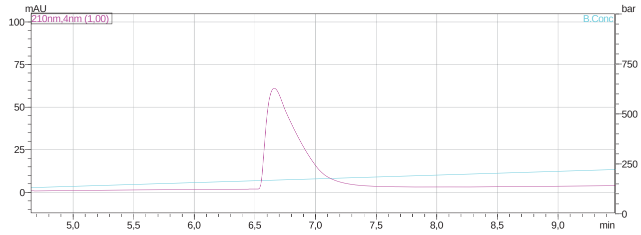

42, AL152

Dataf ile Name:AL152_011.lcd
Sample Name:AL152 Sample
ID:STD-0004

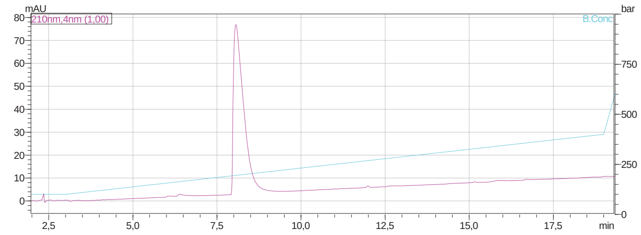

Dataf ile Name:AL152_011.lcd
Sample Name:AL152 Sample
ID:STD-0004

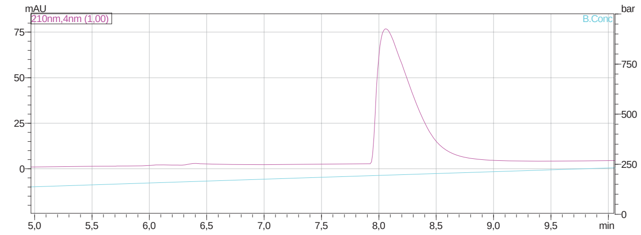

Supplement: Supplementary file 3 — Online Resource 3: NMR and HPLC plots for final compounds 1–18, 27–31, and 36–42. (PDF 4013 kb) [file 10822_2014_9808_MOESM3_ESM.pdf]
